# Academic response to improving value and reducing waste: A comprehensive framework for INcreasing QUality In patient-oriented academic clinical REsearch (INQUIRE)

**DOI:** 10.1371/journal.pmed.1002580

**Published:** 2018-06-07

**Authors:** Belinda von Niederhäusern, Gordon H. Guyatt, Matthias Briel, Christiane Pauli-Magnus

**Affiliations:** 1 Clinical Trial Unit, Department of Clinical Research, University of Basel and University Hospital Basel, Basel, Switzerland; 2 Department of Health Research Methods, Evidence, and Impact, McMaster University, Hamilton, Ontario, Canada; 3 Basel Institute for Clinical Epidemiology and Biostatistics, Department of Clinical Research, University of Basel and University Hospital Basel, Basel, Switzerland; Monash University, AUSTRALIA

## Abstract

**Background:**

Compelling evidence has demonstrated that a large proportion of investment in biomedical research is wasted; this waste is avoidable. Academic institutions have, thus far, shown limited response to recommendations for increasing value and reducing waste. We formulated an academic response by (i) achieving consensus across a wide range of stakeholder groups on a comprehensive framework for quality of patient-oriented clinical research and (ii) highlighting first successful examples of its operationalization to facilitate waste-reducing strategies at academic institutions.

**Methods and findings:**

Based on a systematic review of quality definitions, concepts, and criteria in the medical literature (systematic MEDLINE search up to February 15, 2015, with independent and in duplicate article selection) and on stakeholder websites from 13 countries (Australia, Austria, Canada, France, Germany, Italy, Japan, Norway, Spain, Sweden, Switzerland, United Kingdom, and United States), we systematically developed a comprehensive framework for the quality of clinical research. We identified websites through personal contacts with experts in clinical research or public health who also suggested, for each country, websites of the following 7 stakeholder groups: patient organizations; academic research infrastructures; governmental bodies; regulatory agencies; ethics committees; the pharmaceutical industry; and funding agencies. In addition, we searched websites of inter- or supranational bodies involved in clinical research until no further insights emerged. After consolidation of the identified definitions, concepts, and criteria of quality in a basic framework structure, we conducted 4 rounds of an adapted online Delphi process among the same 7 stakeholder groups from 16 countries. The Delphi process ultimately achieved consensus on structure and content. The framework addresses 5 study stages (concept, planning and feasibility, conduct, analysis and interpretation, and reporting and knowledge translation) and includes the following dimensions: (i) protection of patient safety and rights, (ii) relevance/patient centeredness and involvement, (iii) minimization of bias (internal validity), (iv) precision, (v) transparency/access to data, and (vi) generalizability (external validity) of study results. These dimensions interact with 2 promoters—infrastructure and sustainability through education—that include a set of factors that may enhance all listed quality dimensions. Each quality dimension contains specific questions and explanatory items that guide quality assessment at each research stage from conceptualization of the research question through reporting and knowledge translation of study results. In the last survey round, Delphi participants from 9 countries (Austria, Australia, Canada, Germany, Italy, the Netherlands, Switzerland, UK, and US) agreed on the structure, content, and wording of the research stages, quality dimensions, specific questions, and descriptive examples of the final framework. In Switzerland, INQUIRE has resulted in a roadmap that guides initiatives to increase value within the Swiss Clinical Trial Organization network and through affiliated researchers.

**Conclusions:**

We present a framework based on a consensus of different stakeholder groups guiding the practical assessment of clinical research quality at all stages of a research project. Operationalization of this common structure will support the increase of value by guiding academic institutions and researchers in developing quality enhancement initiatives, from posing the right research question to the transparent publication of results.

## Abstract 2

Belinda von Niederhäusern and colleagues present INQUIRE, a framework based on Delphi-process consensus of stakeholder groups, to guide the practical assessment of quality at all stages of a clinical research project.

## Author summary

**Why was this study done?:**

- A large amount of biomedical research is not useful and thus wasteful; academic institutions in particular have failed to take responsive measures to reports of research waste.
- The lack of a common understanding of the key principles of good clinical research and the lack of practical guidance on how to improve the current situation inhibit efforts to increase value within the academic system.

**What did the researchers do and find?:**

- We present INQUIRE (INcreasing QUality In patient-oriented academic clinical REsearch), a consensus-based framework that supports academia in developing quality enhancement initiatives and setting research agendas to increase efficiency and quality of patient-oriented research at all study stages, i.e., from conceptualization of the research question to reporting of study results and knowledge translation.
- In contrast to existing frameworks, 7 international stakeholder groups, including patient representatives, participated in the development of INQUIRE and agreed on the final product. INQUIRE is therefore based on a common understanding of factors contributing to clinical research quality.

**What do these findings mean?:**

- INQUIRE can support academic institutions and researchers to design quality enhancement initiatives for clinical research. In the future, other stakeholders may adapt INQUIRE for their own environment and specific purposes.
- In Switzerland, INQUIRE has inspired major stakeholders to agree on a national roadmap to improve clinical research, and is currently undergoing implementation in academia.

## Introduction

Clinical research should generate trustworthy evidence that informs decision-making in clinical practice, health policy, and further research [1]. Evidence on the sources and extent of waste in research has, however, highlighted problematic research question selection, poor study design and execution, and non-publication and selective reporting [2–6]. Clinical research stakeholders have expressed concerns that the current model for conducting studies is unaffordable, unsustainable, and, for the generation of new knowledge, seriously flawed [1,7–13]. Low-quality clinical research not only may result in invalid data or distorted outcomes [14], but is also unethical and compromises patients’ safety and rights. In 2014, the “Increasing Value, Reducing Waste” series in *The Lancet* [8–13] provided a compelling body of evidence for sources of waste in biomedical research, including patient-oriented clinical research. The authors made 17 recommendations and formed an alliance (the Reduce Research Waste and Reward Diligence [REWARD] campaign; https://www.thelancet.com/campaigns/efficiency/statement) to increase value, involving stakeholders that include funders, regulators, journal editors, academic institutions, and researchers.

A follow-up article in 2016 offered an overview of the initial stimulus of REWARD across stakeholder communities [2]. Although the authors noted innovation and momentum for corrective actions by some stakeholder groups, they emphasized that, overall, there has been little recognition of the series by academic institutions and very limited implementation of corrective actions.

As a major driving force of patient-oriented clinical research, academia is ideally placed to lead the movement to increase value. Academia not only receives substantial public funds [15,16] but also produces the majority of scientific publications [17]. Despite significant investments by the research community in infrastructure, training, and methodological support [18,19], the issues raised by REWARD persist. Potential reasons for slow progress and uptake may include a lack of common academic policies across a complex ecosystem of stakeholders [2].

Medical specialties and expert groups, ethics committees, regulatory bodies, funding agencies, industrial partners, and patients all have a say in academic research. Their perceptions on what constitutes “good clinical research” and what is “waste,” however, remain vague and, to the extent they are articulated at all, vary [20]. Lack of an established framework reflecting a common understanding of the pillars that frame quality of clinical research may be inhibiting efforts to increase value in academia, and across the system. Practical guidance on how to improve the current situation, however, may not become effective until consensus on these pillars exists.

Therefore, we aimed to develop a comprehensive framework for quality of patient-oriented clinical research that is based on the consensus of key major stakeholder groups—and covers key research stages. Here we present the INcreasing QUality In patient-oriented academic clinical REsearch (INQUIRE) framework, describe its development, and highlight examples of its successful operationalization for the purpose of generating more useful clinical research. INQUIRE is intended to guide academic institutions and researchers in developing quality enhancement initiatives. INQUIRE may also be useful for institutions in setting their research agendas, from posing patient-relevant research questions to the transparent publication of results. In the future, the framework and its operationalization may thus provide a possible supporting structure for the REWARD campaign to increase value and reduce waste.

## Methods

This study is reported according to the Standards for Reporting Qualitative Research (SRQR, http://www.equator-network.org/reporting-guidelines/srqr/) as described in S6 Appendix.

INQUIRE covers different types and phases of clinical research, which we here define as research conducted with patients to answer therapeutic, preventive, diagnostic, or prognostic questions, to investigate the mechanisms of diseases, or to develop new technologies for therapy, prevention, or diagnosis of diseases.

INQUIRE was developed based on 3 principles: (i) integrating empirical evidence on aspects of quality through a systematic review [20] informing a first matrix of quality dimensions, (ii) including the views of a broad range of stakeholder representatives [21–24] through iterative rounds of a modified online Delphi process [25–27], and (iii) addressing operationalization of the framework through detailed feedback of stakeholders from the Swiss academic setting.

S1 Appendix presents a detailed description of the framework development and the consensus process. In short, applying the framework method according to Gale, as detailed in [20], we consolidated the definitions, criteria, and themes of clinical research quality identified through our systematic review into a comprehensive framework matrix [28]. We first coded quality definitions, criteria, or themes into quality items (i.e., single aspects of quality) and grouped them thematically and according to stages of research. We conducted iterative consultation addressing the comprehensiveness and presentation of the framework until we reached consensus.

### Ethics statement

As the Delphi process did not include health-related data and was therefore not within the scope of the applicable Swiss Human Research Act (HRA, Art. 1), it did not require formal ethical approval, but we obtained a declaration of no obligation (Req-2017-00311) from the Ethics Committee Northwest/Central Switzerland (EKNZ).

### Delphi process

The Delphi process took place between September 2015 and June 2017. To allow for broad inclusion of perspectives, we considered the same 7 stakeholder groups to be relevant as in the preceding systematic review [20], i.e., patient organizations, academic research infrastructures, governmental bodies, regulatory agencies, ethics committees, the pharmaceutical industry, and funding agencies. Our team, with help from affiliated collaborators and by word of mouth among the related networks (e.g., European Patients’ Academy on Therapeutic Innovation [EUPATI] for patient representatives), identified potential stakeholder representatives from 16 countries. We recruited participants on the basis of awareness of quality issues related to clinical research and ability to provide feedback within a specified time window. After inviting participants through SurveyMonkey (https://www.surveymonkey.net), we conducted 2 Delphi rounds aimed at (i) identifying any additional quality items that we had not yet considered and (ii) establishing broad consensus across stakeholders on the overall framework structure. After each round, we shared with respondents a summary of the adaptations made based on their suggestions in the previous round and asked for their agreement or further suggestions for improvements on structure and content.

We conducted 2 further Delphi rounds seeking consensus on how to operationalize the framework structure, with a focus on the Swiss academic setting. We invited an additional 33 stakeholder representatives from Switzerland, particularly academics. Specifically, we invited representatives (board members and executive directors) of all 6 Swiss Clinical Trial Units (CTUs) at university hospitals and members of the executive committee and the Quality Working Group at the Swiss Clinical Trial Organization (SCTO). In order to allow for the operationalization of the framework, for these rounds we rephrased the previous “quality items” as “specific quality questions” accompanied by “descriptive examples.” First, we addressed framework structure, content, and wording of specific quality questions and corresponding examples. Second, we provided respondents with all anonymized comments, a response by the authors to each comment, and the overall agreement score on framework structure and specific quality questions. We provide the SurveyMonkey (round 1–3) and email (round 4) questionnaires for all Delphi rounds in S1 Appendix. Consensus was predefined as an agreement of 80% or higher. In each round, if survey participants had specific questions or found it difficult to understand the framework’s concepts, we offered them personalized explanations. Two patient representatives and 4 representatives of academic CTUs accepted this offer in the third Delphi round. BvN, MB, or CPM held one-on-one meetings or telephone calls (once was enough in all cases) that served to answer the survey participant’s questions regarding the overall framework structure, specific quality questions, examples, or the wording. We took notes on their suggestions, incorporated them in the adapted framework version, and fed them back to the other Delphi participants in the fourth Delphi round.

### Data analysis

We conducted qualitative analysis for open-ended questions and descriptively summarized comments and suggestions for removal, addition, or adaptation of quality dimensions and of individual quality items and key themes based on repetition of concept words. Through discussion, we iteratively adapted the framework structure and content and fed back revisions to the survey participants. We calculated agreement scores in percentages by dividing the number of participants agreeing by the total number of participants who provided an answer to the respective question.

### Patient involvement

This research was designed and conducted with the involvement of 7 stakeholder groups, including patient participants. We actively sought patients’ priorities and preferences. Patients, if required, received plain language explanations of the framework content, and provided their opinion on the structure and content of the framework in the 4 Delphi rounds. After completion of the Delphi process, all stakeholder representatives, including patients, received the final framework.

## Results

### The structure of INQUIRE

We invited 109 representatives from 7 stakeholder groups and 16 countries to participate in the Delphi process. Response rates ranged from 53.0% in round 1 to 98.1% in round 4, and from 12.5% for governmental participants to 83.3% for pharmaceutical industry participants (Tables 1 and 2). The proportion of academic participants varied from 48.3% in round 1 to 60.4% in round 4. The range of covered disciplines included—but was not limited to—clinical epidemiology and biostatistics, clinical pharmacology and toxicology, dermatology and allergology, general internal medicine, health services research and health policy, immunology, insurance medicine, nursing sciences, public health, oncology, pediatrics, patient engagement research, pharmaceutical medicine, and quality improvement research (see Table 2 legend for full list).

**Table 1:** Response rates by stakeholder group for Delphi rounds 1 and 2.

Stakeholders	Number of participants invited (thereof (Swiss)	Round 1		Round 2	
		Number of respondents (thereof Swiss)	Total response rate, percent^1^	Number of respondents (thereof Swiss)	Total response rate, percent^1^
Patient groups/representatives	20 (19)	10 (10)	50.0	7 (7)	70.0
Academia	39 (11)	28 (8)	71.8	23 (8)	82.1
Pharmaceutical industry	12 (5)	10 (5)	83.3	6 (4)	60.0
Ethics committees/IRBs	8 (5)	4 (3)	50.0	3 (2)	75.0
Governmental bodies and regulatory bodies	24 (2)	3 (1)	12.5	3 (1)	100
Funding agencies	6 (2)	3 (2)	50.0	3 (2)	100
**Number of countries**^**2**^	16	13		11	
**Total number or percent**	**109**	**58**	**53.2**	**45**	**77.6**

^1^Response rates were calculated based on the number of respondents in each round compared to the respondents in the previous round; only respondents were invited to participate in further rounds of the survey.

^2^For international organizations or companies: location of headquarters. Countries represented at end of round 1 (*n* = 13): Austria, Australia, Belgium, Canada, Switzerland, Germany, Spain, France, Italy, Japan, Netherlands, UK, US. Countries represented at end of round 2 (*n* = 11): Austria, Belgium, Canada, Switzerland, Germany, Spain, France, Italy, Netherlands, UK, US.

IRB, institutional review board.

**Table 2:** Response rates by stakeholder group for Delphi rounds 3 and 4.

Stakeholders	Round 3					Round 4	
	Number of participants invited from round 2 (thereof Swiss)^1^	Number of respondents (thereof Swiss)	Number of additional invited participants^2^ (Number of respondents)	Total Number of respondents (thereof Swiss)	Total response rate, percent (thereof Swiss)^3^	Number of respondents^4^ (thereof Swiss)	Response rate, percent (thereof Swiss)
Patient groups/representatives	7 (7)	3 (3)	0 (0)	3 (3)	42.9 (42.9)	3 (3)	100 (100)
Academic representatives^5^	23 (8)	14 (5)	28 (19)	33 (24)	64.7 (66.7)	32 (24)	97.0 (100)
National research institutions	6 (4)	5 (4)	9 (4)	9 (7)	60.0 (53.8)	9 (7)	100 (100)
Clinical investigators	4 (2)	2 (1)	5 (4)	7 (6)	77.8 (85.7)	7 (6)	100 (100)
Academic Clinical Trial Units	0 (0)	0 (0)	11 (9)	9 (9)	81.8 (81.8)	9 (9)	100 (100)
Methodological research	13 (2)	7 (2)	3 (2)	9 (2)	56.3 (40.0)	8 (2)	88.9 (100)
Pharmaceutical industry	6 (4)	4 (2)	1 (1)	5 (3)	71.4 (60.0)	5 (3)	100 (100)
Ethics committees/IRBs	3 (2)	3 (2)	1 (1)	4 (3)	100 (100)	4 (3)	100 (100)
Governmental bodies and regulatory bodies	3 (2)	3 (2)	2 (2)	5 (4)	100 (100)	5 (4)	100 (100)
Funding agencies	3 (2)	3 (2)	1 (1)	4 (3)	100 (100)	4 (3)	100 (100)
**Number of countries**	11	9	2	9^6^		9^6^	
**Total number or percent**	**45 (24)**	**30 (16)**	**33 (24)**	**54 (40)**	**69.2 (70.2)**	**53 (40)**	**98.2 (100)**

^1^All respondents from round 2 were invited to participate in round 3.

^2^Predominantly representatives of Swiss academia; *n* = 3 were non-Swiss.

^3^Response rates were calculated based on the number of participants invited from round 2 and the additional Swiss participants invited for round 3 and 4 only.

^4^Participants who responded to round 3 were invited to participate in round 4.

^5^Disciplines covered by representatives of national research institutions: clinical epidemiology, clinical pharmacology and toxicology, dermatology and allergology, general internal medicine, immunology, public health, oncology, pediatrics, patient engagement research, pharmaceutical medicine, and quality improvement research. Clinical investigators: physicians conducting research in anesthesiology, clinical pharmacology and toxicology, general internal medicine, neurology and clinical neuroscience, nursing sciences/patient-centered care, pediatrics, and public health. Academic Clinical Trial Units: managing directors or their deputies with scientific backgrounds in allergology and immunology, clinical epidemiology and biostatistics, general medicine, physiology, and pneumology. Methodological researchers cover evidence-based medicine, including specializations in evidence synthesis, clinical epidemiology and biostatistics, health services research and health policy, insurance medicine, and public health.

^6^Countries represented at the end of rounds 3 and 4 (*n* = 9): Austria, Australia, Canada, Switzerland, Germany, Italy, Netherlands, UK, US.

IRB, institutional review board.

**Fig 1:**
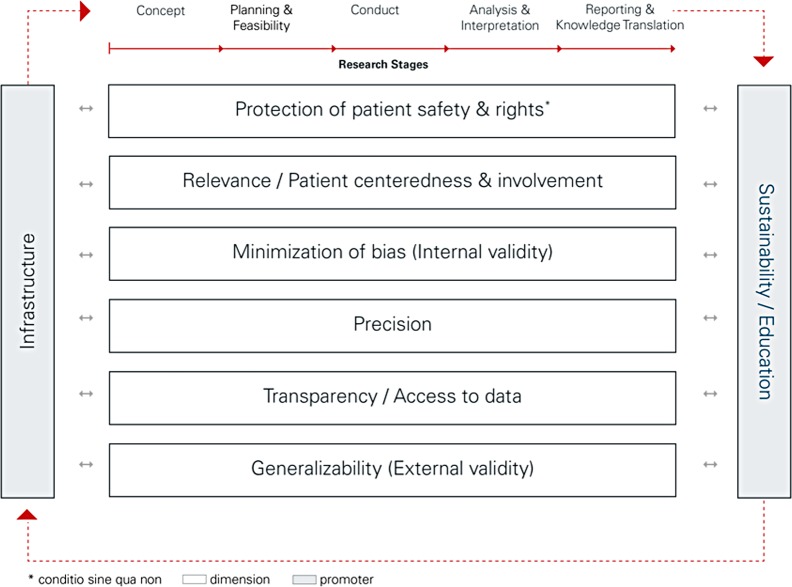
Structure of INQUIRE, a framework to increase the quality of academic clinical research.

After an agreement of 53.1% of respondents in round 1, 97.1% in round 2, and 87.0% in round 3, 100% of survey respondents from 9 countries agreed on a final framework structure (i.e., all building blocks, wording, and order) in round 4. The final agreement on content ranged from 96.2% (sustainability through education) and 98.1% (planning and feasibility) to 100% for all other stages and the other promoter (see S2 Appendix for detailed agreement rates). S3 Appendix presents all versions of the framework with track changes and the authors’ reply to comments. The final framework is structured in 3 main building blocks (Fig 1):

i) 6 quality dimensions, with a dimension being defined as an overarching concept of quality containing multiple individual quality questions (Box 1): (1) protection of patient safety and rights; (2) relevance of study question and patient centeredness and involvement; (3) minimization of bias (internal validity); (4) precision; (5) transparency and access to data; and (6) generalizability (external validity) of study results,
ii) 5 successive study stages (concept, planning and feasibility, conduct, analysis and interpretation, and reporting and knowledge translation) to which the dimensions apply, and
iii) 2 quality promoters (infrastructure and sustainability through education), with a promoter being defined as a set of factors that may enhance all quality dimensions at a research institution.

**Box 1. Description of quality dimensions and promoters**

**Quality dimensions**

- Protection of patients’ safety and rights represents the cornerstone of clinical research. It assures that researchers, at all times, respect and protect participants’ safety, rights, and well-being.
- Research relevance and patient centeredness and involvement reflects the extent to which the research question is scientifically and societally beneficial (i.e., leads to improved decision-making in healthcare) and involves patient values and preferences.
- Minimization of bias—or internal validity—reflects the extent to which the research minimizes systematic error (bias) through selecting an appropriate study design and prespecifying analyses.
- Precision reflects the extent to which the research minimizes random error (i.e., sufficiently narrow confidence intervals) and to what extent authors present precision to facilitate researcher users’ understanding.
- Transparency and access to data reflects the extent to which study planning, conduct, data collection, and presentation of results are transparent and accessible for the scientific community and the public. This dimension includes the registration of the study in a publicly accessible database, publication of the full study protocol, publication of the study results—independent of their effect size or direction—and explicitly encouraging access to the full patient-level dataset (data sharing).
- Generalizability—or external validity—reflects the extent to which study results are applicable and generalizable to the wider patient population in real-life circumstances.

**Quality promoters**

- The first quality promoter, an established research infrastructure, includes well-trained personnel and functional facilities on-site.
- The second quality promoter, sustainability through education, supports sustainability of a developed infrastructure through effective involvement and hands-on training of young and senior investigators as well as competent mentoring and early career development and continuous education of study personnel to secure a productive clinical research environment in the long term.

S2 Appendix presents a detailed description of the dimensions and promoters and empirical evidence supporting their importance.

Box 1 provides a brief description of each dimension and promoter.

The framework captures a comprehensive generic set of quality dimensions and 77 specific quality questions accompanied by descriptive examples that can be adapted to different study designs, indications, or treatments. Quality dimensions are not represented hierarchically, and those using the framework may, depending on the setting, apply weights to items or dimensions.

### Operationalization of the framework

#### Quality by research stage

Applying the framework in the academic clinical research context requires guidance on its operationalization. In contrast to the existing quality assessment tools or checklists [20], the INQUIRE framework includes key research stages from conceptualization to reporting and knowledge translation, and 2 supporting quality promoters. During Delphi rounds 1 and 2, participants commented on the characterization of study stages and ultimately achieved consensus. The first study stage, conceptualization, starts with a clinical knowledge gap and ends with a clearly defined research question and appropriate study design. During the second stage, planning and feasibility, the investigators develop a protocol based on their research question and assess the feasibility of their undertaking. This stage ends with approval of the protocol by regulatory bodies (if such approval is necessary). The third stage, study conduct, starts with the first patient recruited and ends with last patient visit. During this stage, the investigators conduct the study according to the protocol. In stage 4, analysis and interpretation, the investigators process and interpret the study data. The last stage, reporting and knowledge translation, covers key activities after analysis of the data, i.e., publication, dissemination, uptake in clinical practice, guidelines and knowledge translation, and archiving of study results.

#### Specific quality questions and descriptive examples

For each quality dimension and research stage or promoter, we developed specific quality questions on which the Delphi participants commented, and on which—following revision—they ultimately agreed (Table 3; see Table A in S2 Appendix for agreement scores and S3 Appendix for all versions of the framework). The specific quality questions provide a detailed description of the dimensions’ content and illustrate what quality aspects should be addressed and answered in each study stage. Such specific questions are explicitly designed to be broadly applicable to different study designs. In addition, “descriptive examples” complement the specific questions and provide guidance on how to operationalize the framework for a particular study type or setting, referencing already existing checklists and tools. In particular, consideration of patients’ priorities and preferences such as access to participation in research, reporting and accessibility of study results to participants, and access to care during and after a study are specifically reflected for each study stage as specific questions and descriptive examples.

**Table 3:** INQUIRE’s specific quality questions (and descriptive examples for Stage I) by quality dimension and research stage, and promoters.

**Dimension or promoter**	Specific question*	**Examples**
***Study stage I*: *Concept*** ***Milestone*: *Research question including study type defined and viable***		
**Protection of patient safety and rights**	Can the research question be addressed in the given setting?	Based on a rough resource assessment, and potentially available study participants, is it feasible to answer the research question?
		Based on a rough budget estimate, is it feasible to answer the research question with a specified study type?
	Does the study consider equity appropriately?	Are participants selected so that • vulnerable individuals are neither targeted for risky research nor withheld from research relevant to these populations? •socially powerful individuals are not favored for potentially beneficial research?
	Is the research design adequate for the stage of an investigated technology to ensure patient safety?	Are sufficient data on toxicity/teratogenicity of an intervention available from animal studies or phase I studies?
	Do the (assumed) short- and long-term benefits of the study outweigh potential risks associated with the study (consistent with clinical equipoise)?	
**Relevance/patient centeredness and involvement**	Is significant potential add-on value to existing evidence (systematic review) specified, taking into consideration burden of disease and anticipated benefit of treatment?	Are uncertainties in existing evidence identified and discussed in a systematic review?
		Does research •expand or challenge current knowledge? •open additional areas for new research activity? •justify replication of existing evidence, if applicable?
	Are patient representatives/advocates and their needs and values involved in the development of the research question?	
	Are outcome measures patient-relevant?	Are outcomes patient-relevant according to COMET [29], including quality of life, if applicable, and with judicious use of surrogate endpoints?
**Minimization of bias (internal validity)**	Is the selected study type/design appropriate to minimize bias?	Is the study randomized or, if not, appropriately controlled for confounding?
	Are potential sources of bias anticipated, evaluating the magnitude and the likely direction?	
	Are outcome measures well-defined, prespecified, valid, reliable, and measured at appropriate times?	Are outcomes •well-defined (up-front)? •valid (measure what they intend to measure)? •reliable (stable and consistent when repeatedly measured)? •sensitive to important change? •measured at appropriate times? •standardized across studies (core outcome sets, if applicable)?
**Precision**	Has an estimate of the required sample size been made (for feasibility purposes, see “Protection of patient safety and rights”)?	
**Transparency/access to data**	Is the research question clearly specified (e.g., in a synopsis)?	Is each component of P(I/E)(C)O [29] as applicable to study design clearly defined, i.e.: •patient population to be recruited/investigated in the study, •intervention to be assessed, •exposure to be assessed, •diagnostic test to be assessed, •control intervention as comparator, and •outcomes to be measured?
**Generalizability (external validity)**	Are planned study participants representative of patients who would use the drug/intervention/diagnostic test in a real-life setting?	Are unnecessary restrictions through inclusion/exclusion criteria avoided (to facilitate rapid accrual, broader generalization, pragmatic study conduct)?
		Is the control group adequate given current evidence and clinical practice (e.g., “standard of care” rather than “no treatment”)?
***Study stage II*: *Planning and feasibility*** ***Milestone*: *Protocol developed and approved by regulatory bodies***		
**Dimension or promoter**	Specific question*	
**Protection of patient safety and rights**	Do the potential short- and long-term benefits of the study outweigh study burden (due to study visits, intervention, procedures, etc.)?	
	Are patients’ safety and rights protected through the study’s adherence to applicable national and international regulations and laws?	
	Has feasibility been checked thoughtfully based on existing evidence (systematic review)?	
	Is collection, documentation, and reporting of adverse events/serious adverse events/suspected unexpected serious adverse reactions according to the applicable regulations planned and specified in the protocol?	
	Are mechanisms established (e.g., through data monitoring committees) that allow early study termination when required and prevent early study termination for inadequate reasons?	
**Relevance/patient centeredness and involvement**	Has knowledge transfer/use been considered (e.g., plans to take account of results in clinical guidelines)?	
**Minimization of bias (internal validity)**	Is statistical analysis prespecified (using outcomes as defined in concept stage)?	
	Is study monitoring (adapted to risk of study, if applicable) planned and documented in a monitoring plan, including by a data monitoring committee?	
	Is data management planned and documented in a data management plan?	
	Has minimization of bias been planned for in the study design, taking account of the research question?	
**Precision**	Are expected treatment effects and event rates in intervention and control groups realistic and estimated based on empirical evidence?	
	Are recruitment procedures and recruitment monitoring planned to ensure sufficient sample size?	
**Transparency/access to data**	Does the protocol accord with established standards (e.g., SPIRIT [30] or other applicable guidelines depending on study design)?	
	Has the study been registered with a publicly accessible study database (e.g., ClinicalTrials.gov)?	
	Is there a dissemination plan for sharing study information, including the protocol, summary results, and participant-level data?	
**Generalizability (external validity)**	Are study procedures/observations in line with routine practice in the given setting?	
***Study stage III*: *Conduct*** ***Milestone*: *Last patient last visit***		
**Protection of patient safety and rights**	Is respect for and consideration of patient rights, well-being, and dignity guaranteed throughout conduct of study?	
	Is patient safety guaranteed throughout conduct of study?	
	Is the study being conducted according to protocol?	
	Is there monitoring of compliance of participants and study staff with the protocol?	
	Are patients’ safety and rights protected through the study’s adherence to applicable national and international regulations and laws?	
**Relevance/patient centeredness and involvement**	Are there any measures in place to assure study participants’ involvement, cooperation, and feedback throughout conduct of study (e.g., incentives or phone calls)?	
**Minimization of bias (internal validity)**	Are data systematically collected as prespecified in the protocol?	
	Is monitoring being conducted according to the prespecified monitoring plan?	
**Precision**	Is enrollment of study participants monitored?	
	Is variability of study procedures and measurement error minimized, e.g., by using centralized monitoring strategies?	
**Transparency/access to data**	Is study conduct transparent to all involved parties?	
**Generalizability (external validity)**	Are numbers of participants through different stages of a study documented (patient flow), including reasons for leaving the study prematurely (if voluntarily provided by patients)?	
***Study stage IV*: *Analysis and interpretation*** ***Milestone*: *Study data analyzed and interpreted***		
**Protection of patient safety and rights**	Does data sharing adhere to appropriate data protection policies?	
**Relevance/patient centeredness and involvement**	Are data analyzed to maximize the use of results by different stakeholders?	
**Minimization of bias (internal validity)**	Are the data analyzed as prespecified in the protocol/statistical analysis plan?	
	Has there been statistical adjustment using key confounding variables in the analysis (e.g., multivariable analysis), if applicable?	
	Does the analysis follow an adequate strategy to deal with participants in whom treatment or follow-up was not in accordance with study protocol?	
	Have results been interpreted with least possible “spin” (e.g., without intentionally implying greater or lesser effects than have actually been shown by the data)?	
**Precision**	Is the uncertainty of results considered in the analysis?	
**Transparency/access to data**	Is the analysis code clearly documented, and is the analysis process reproducible?	
	Are deviations from the statistical analysis plan or protocol adequately documented and reported?	
**Generalizability (external validity)**	Does the interpretation put the results adequately into context of clinical practice/public health?	
***Study stage V*: *Reporting and knowledge translation*** ***Milestone*: *Study archived and published***		
**Protection of patient safety and rights**	Is study completion/termination communicated to appropriate parties and documented in registries?	
	Are study participants informed about the outcome/main findings of the study in plain language (including treatment allocation of participant, if applicable)?	
	Do study participants get access to products/interventions after study, if applicable?	
**Relevance/patient centeredness and involvement**	Do authors critically reflect on research findings (results as well as challenges or mistakes during study conduct) and the implications for future research?	
	Is the study easily available to decision-/policy-/guideline-makers?	
	Are study patients/patient representatives involved in reporting the study?	
**Minimization of bias (internal validity)**	Are all outcomes and important study characteristics reported, as prespecified in the protocol (outcome reporting bias prevented)?	
**Precision**	Are absolute and relative treatment effects reported, accompanied by confidence intervals?	
	Is the analysis set of participants clearly specified?	
**Transparency/access to data**	Is dissemination of data and study results maximized?	
	Are reporting guidelines followed, to facilitate critical appraisal and reproducibility?	
	Are selective reporting, “spin,” and plagiarism avoided, and conflicts of interest declared?	
	Is knowledge transfer and exchange fostered?	
	Are study records and datasets kept and archived for at least the legally required period of time?	
**Generalizability (external validity)**	Is potential impact on clinical practice/public health outlined in publicly accessible research reports (e.g., journal publication)?	
	Are characteristics of included participants clearly reported?	
	Are the results of prespecified subgroup analyses, if applicable, reported to help assess the importance of key participant characteristics (e.g., disease severity, age, or gender)?	
***Quality promoters***		
**Sustainability/education**	Are doctoral students, junior researchers, clinicians, or patient advocates actively involved in all stages of a clinical study and reliably supervised/mentored by senior researchers, and are their specific contributions acknowledged appropriately?	
**Infrastructure**	Is a quality management system including standard operating procedures in place?	
	Are well-trained, experienced, and dedicated principal investigators and study staff present?	
	Are expert epidemiologists/methodologists, statisticians, professional data managers, and/or a logistical support unit involved early on?	
	Are adequate human, material, and equipment resources available for study conduct?	
	Are adequate facilities ensuring data security and privacy in place (including competent and effective IT support to facilitate solutions tailored to specific challenges of individual studies or agreement templates for doctoral students with respect to data privacy and confidentiality)?	
	Is inter-/multidisciplinary collaboration and involvement in study planning and conduct fostered?	
	Are all institutions involved in the study covered by compulsory liability insurance?	
	Is an overview of the existing research infrastructure available and accessible to any researchers with a study idea?	

*S4 Appendix and https://dkf.unibas.ch/inquire present a full list of specific quality questions accompanied by descriptive examples.

Box 2 provides an example of how INQUIRE may be applied in practice. S4 Appendix provides the full list of specific quality questions and descriptive examples by research stage and promoter.

**Box 2. Applying INQUIRE in practice: Specific quality questions and descriptive examples**

Specific quality questions support the INQUIRE user to cover all relevant quality aspects by dimension and research stage. Descriptive examples provide more detailed information on what the specific question entails. Assuming, for example, that we are currently planning a study, we take the “Planning and Feasibility” section of the framework and look at the specific quality questions, as well as the descriptive examples, as provided in Table 3 and S4 Appendix. One specific question in the “Protection of patient safety and rights” dimension, for example, is “Has feasibility been checked thoughtfully based on existing evidence?” As a user, we may now assess this specific question for our particular study. The descriptive examples provide more guidance on the question’s content:

- Are valid and robust preclinical data present (if applicable)?
- Have crucial feasibility aspects (e.g., recruitment) been piloted?
- Are recruitment assumptions realistic in a specified timeframe (e.g., empirical data from electronic health records or from pilot study present)?
- Have national/international study registries been checked for studies that could interfere with the planned study?
- Do anticipated study costs (preparation, conduct, analysis, dissemination) match with available budget?
- Are study cost data related to planning, conduct, analysis, and dissemination planned to be collected (if applicable)?

Descriptive examples do not constitute a complete list but rather provide guidance on how to interpret the specific quality question.

#### First applications in the Swiss academic setting

**Fig 2:**
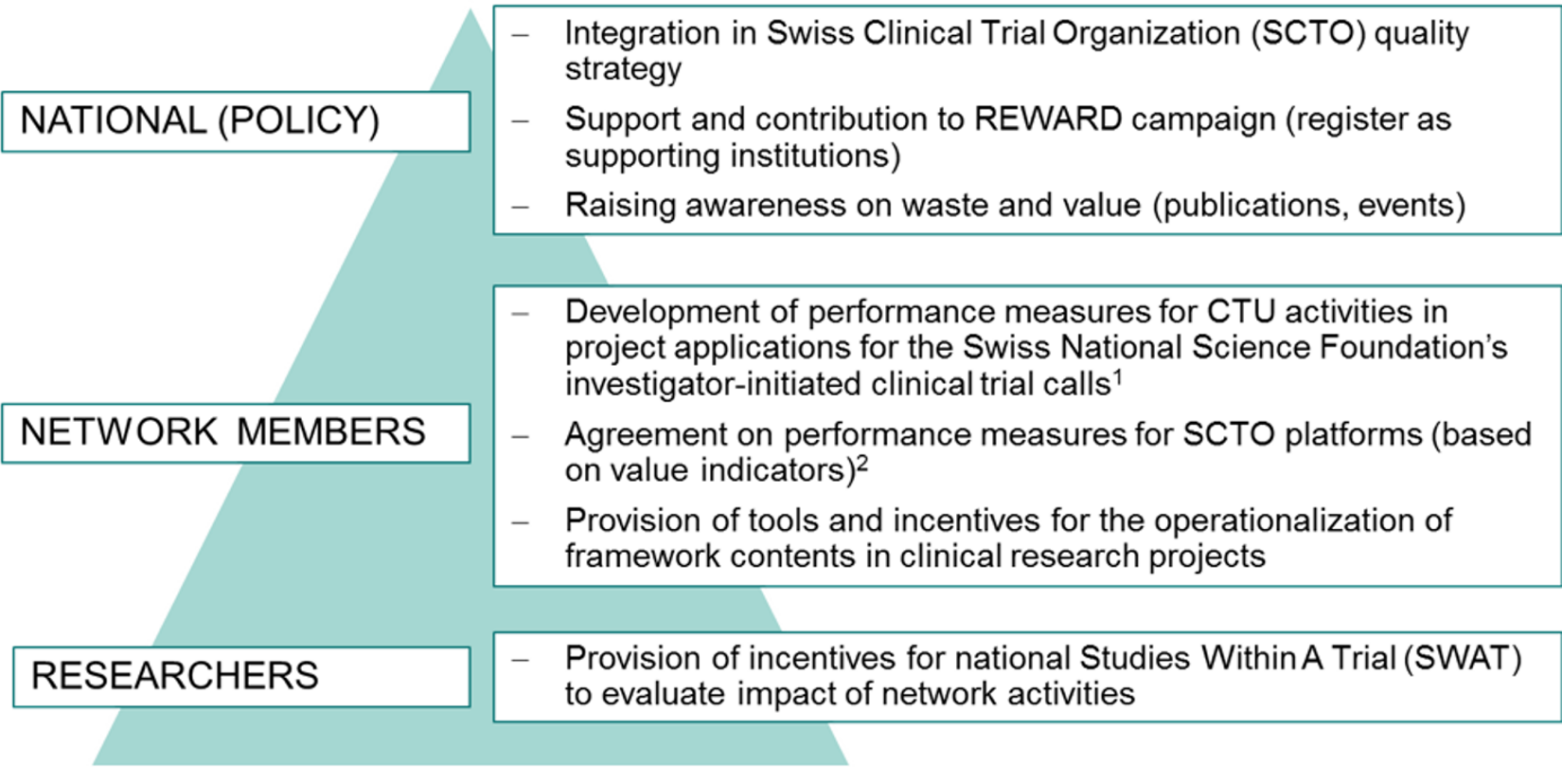
SCTO roadmap on how to increase value in Swiss academic clinical research. ^1^The CTU Network and SCTO actively support the design, planning, and conduct of investigator-initiated studies that apply for the Swiss National Science Foundation’s call for investigator-initiated clinical trials (http://www.snf.ch/en/funding/programmes/iict/Pages/default.aspx). The Swiss National Science Foundation has earmarked CHF 10 million for the 2017 funding period. Performance measures would allow assessing the SCTO network’s impact on the success of supported studies. ^2^SCTO platforms are excellence clusters for specific support units, e.g., data management, statistics, training, and education, located at different CTUs. CTU, Clinical Trial Unit; REWARD, Reduce Research Waste and Reward Diligence.

The development of the INQUIRE framework inspired several Delphi participants, members of the SCTO, representing the network of CTUs in Switzerland, and other stakeholders to convene a symposium, “Adding Value in Clinical Research: What’s Been Achieved and How Do We Manage New Challenges?,” in June 2017 (https://www.scto.ch/de/event-calendar/symposium/symposium-2017.html) and a stakeholder event, “From Stumbling Blocks into Stepping Stones: Creating Greater Value and Efficiency in Clinical Research,” in January 2018 (https://www.scto.ch/en/event-calendar/forum-clinical-research/forum-2018.html). Along with reflections on international initiatives to improve value by 2 authors of *The Lancet* series and the UK National Institute for Health Research (NIHR), the participants discussed the current situation in Switzerland and the INQUIRE framework. In response, the steering board of the SCTO included INQUIRE into its strategy, is in the process of developing a roadmap, and is taking action on 3 different levels: nationally, through adaptation of its quality policies according to the framework’s dimensions; locally, through the establishment of performance measures and incentives for its network members (CTUs) based on the framework; and individually, through incentivizing academic researchers to evaluate these network activities in Studies Within A Trial—so called SWATs (Fig 2). A description of the INQUIRE framework development as well as tools supporting users to apply the framework in practice are available at https://dkf.unibas.ch/inquire.

At the local level, multiple initiatives are currently ongoing to test the framework’s real-world potential. Box A in S5 Appendix describes selected examples from the University Hospital Basel. All initiatives aim at evaluating the framework’s usefulness through a continuous process of evaluation and, if necessary, refinement.

## Discussion

### Summary

This study represents the first effort to our knowledge involving key stakeholder groups across geographic regions to formulate an academic response to the challenges of avoiding waste and increasing value in clinical research. We offer INQUIRE, a comprehensive, consensus-based framework of clinical research quality composed of 6 quality dimensions and 2 promoters that span the entire lifecycle of a clinical study, from conceptualization of the research question to reporting and knowledge translation of study results. With INQUIRE, high-quality clinical research is defined as relevant, patient-centered, minimal in bias, precise, transparent, accessible, and generalizable while protecting patient safety and rights. Ideally, this research is promoted by and feeds into research infrastructure and education, creating—over the long term—a productive and sustainable clinical research environment.

We designed the framework to increase value through operationalization of its content. Seventy-seven specific quality questions can guide academic institutions and researchers in developing quality enhancement initiatives that they have, thus far, failed to implement. Mapped to 5 study stages, INQUIRE guides academic institutions and the affiliated research force on how to practically (i) set the right research priorities, (ii) use robust research design, conduct, and analysis, (iii) efficiently assess feasibility and manage research, (iv) make information on research methods and findings accessible, and (v) produce complete, unbiased, and usable research reports. The framework and its operationalization thus provide a possible supporting structure for the REWARD campaign (https://www.thelancet.com/campaigns/efficiency/statement) to increase value and reduce waste. In Switzerland, the framework inspired stakeholders to convene a relevant symposium followed by a stakeholder event, resulting in a roadmap on how to use the INQUIRE framework to guide initiatives to increase value within the SCTO network and through affiliated researchers.

### Strengths and limitations

The strength of this study is that our methods achieved a consensus among key stakeholder groups on a prominent, but complex and heretofore ill-defined concept. This work began with an extensive systematic search of existing definitions, approaches, and measurements of clinical research quality across cultures and stakeholder groups that revealed a lack of a common concept. We therefore developed this framework based on empirical evidence and considered the interests of a wide variety of stakeholders. Our response and agreement rates during the final consensus-finding rounds were very high. The resulting guidance is to support academic institutions, researchers, and other stakeholders in the holistic assessment of study quality.

Our study has several limitations. In the Delphi process, participation varied across stakeholder groups, and, therefore, some stakeholder opinions may be underrepresented. For example, patients are underrepresented because we had difficulties identifying patient representatives to comment on later, more complex versions of the framework. Furthermore, all patient representatives who completed all 4 Delphi rounds came from Switzerland. Publishers have not explicitly been considered as a stakeholder group in the process so far but will definitely be included in future validations and adaptations of the framework. In addition, it is possible that some specific disciplines were underrepresented in the process (e.g., community representation), potentially leading to discipline-specific gaps in the framework’s content. We limited stakeholders to those who were willing to reply in English, German, French, or Italian.

While this framework is widely applicable to diverse study designs, its comprehensiveness, with 77 specific questions, might prove challenging. It is possible, however, for institutions to focus on particular parts, or questions, from the framework, and for investigators to focus on sections particularly relevant to their clinical research stage. For example, investigators planning a study may specifically use the quality questions contained within the planning and feasibility section of the framework. At the very least, however, awareness of each of the domains should lead to some consideration of the attendant issues. Similarly, some questions may be applicable to only specific situations (e.g., statistical adjustment for minimizing confounding). Where this is the case, we phrase these questions in the framework with “if applicable.”

Certain criteria or sections of the quality promoters may be difficult to attain in resource-constrained settings and countries. We plan, in collaboration with researchers from the Swiss Public and Tropical Health Institute (https://www.swisstph.ch/en/), to consider what (if any) adaptation of the INQUIRE framework is necessary for lower income settings.

Finally, some important items that would complement the framework were only mentioned during the peer-review process (e.g., compensation for harm, responsiveness to post-publication requests, and timely correction of errors). Due to the Delphi nature of this study, these items will be considered in future validations and adaptations of the framework.

### Next steps and implications

The rapid uptake of the framework in Switzerland has been driven by the broad consensus across Swiss participants of key clinical research stakeholder groups including patients, academia, ethics committees, regulatory and governmental bodies, funding agencies, and the pharmaceutical industry. The development of INQUIRE not only increased stakeholders’ awareness of the “waste issue,” it also represents the conditio sine qua non for any joint future efforts to sustainably increase value. Following up on the national symposium in June and the SCTO roadmap (Fig 2), we will, in 2018, conduct a 1-day strategy workshop engaging a diverse group of Swiss stakeholder representatives and policy-makers on how to drive the framework’s implementation at a national scale.

Due to its broad acceptance across international stakeholders and its theoretical underpinning, the framework may further serve as a common structure for other stakeholder groups. We broadly envision 2 categories of operationalization: First, the framework may act as standard criteria—or a common language—for what constitutes high-quality academic clinical research, thereby increasing awareness and stimulating discussions on joint solutions for increasing value and reducing waste across academia and other stakeholder groups. Second, investigators may operationalize the framework at the study level by using the specific questions in Table 3 and S4 Appendix as a guiding checklist. Methodologists, funding agencies, and others may use INQUIRE to assess whether a study fulfills quality questions at one, multiple, or all study stages, ensuring that the key goals of the REWARD campaign are met. The specific questions may be adapted to a particular setting and presented in various formats (e.g., electronic applications). In line with the 2017 Cochrane–REWARD prizewinner for reducing waste in research—the NIHR’s Adding Value in Research program (Box 3)—the implementation of INQUIRE should be monitored at all stages and undergo continuous evaluation. Boxes B and C in S5 Appendix describe additional scenarios at different organizational and institutional levels in which the framework may be applied.

**Box 3. NIHR’s Adding Value in Research (AViR) program**

“The AViR programme aims to ensure that research funded by the NIHR addresses questions that are relevant to clinicians, patients, and the public; uses appropriate design and methods; is delivered efficiently; results in accessible full publication; and produces unbiased and useable reports. It therefore matches the key goals of the REWARD campaign in tackling waste at every stage of research. Examples of activities promoted by the programme include requiring funding applications for primary research to reference systematic reviews showing what is already known on a topic; ensuring NIHR-funded research is fully reported; and involving patients not only on funding committees, but also in monitoring trials.” (https://www.nihr.ac.uk/about-us/our-purpose/principles/adding-value-in-research.htm; cited 2017 Oct 30).

## Conclusions

The INQUIRE framework defines clinical research quality based on the consensus of key international stakeholder groups. The framework represents a well-developed academic answer to the lack of a common definition of research quality, and the challenge of research waste. As a common structure for operationalizing the assessment of quality of clinical research at academic institutions, INQUIRE will facilitate implementation of waste reduction and value increasing initiatives. To take the field forward, we encourage the research community and interested stakeholder groups to apply the framework, generate evidence on its utility, and transparently and openly share their approaches.

## Supporting information

S1 Appendix Extended methods. (PDF)

S2 Appendix Extended results. (DOCX)

S3 Appendix All versions of the INQUIRE framework. (DOCX)

S4 Appendix Full INQUIRE framework, including specific quality questions and descriptive examples. (DOCX)

S5 Appendix Potential applications of the INQUIRE framework at different levels and by stakeholder groups. (DOCX)

S6 Appendix Standards for Reporting Qualitative Research (SRQR) checklist. (DOCX)

## Abbreviations

CTU: Clinical Trial Unit
INQUIRE: INcreasing QUality In patient-oriented academic clinical Research
NIHR: UK National Institute for Health Research
REWARD: Reduce Research Waste and Reward Diligence
SCTO: Swiss Clinical Trial Organization
