# Supplementary material for: Academic response to improving value and reducing waste: A comprehensive framework for INcreasing QUality In patient-oriented academic clinical REsearch (INQUIRE)
Source: PLoS Med. 2018 Jun 7;15(6):e1002580. doi: 10.1371/journal.pmed.1002580 (PMC5991651; doi:10.1371/journal.pmed.1002580)
Supplement: S1 Appendix — (PDF) [file pmed.1002580.s001.pdf]

S1 Appendix. Extended methods.

Methods

Defining the framework structure

In a previous systematic review[1], we had identified a range of quality definitions, criteria, and themes across different stakeholder groups, as well as a range of quality assessment tools and checklists. These definitions, criteria and themes as well as the derived tools and checklist were consolidated into a comprehensive framework matrix applying the framework method according to Gale [2]. This included the following two- step procedure:

First, we derived the following three structural building blocks for an initial framework (Figure 1):

a) quality **dimensions**, with a dimension being defined as an overarching concept of quality containing multiple individual quality questions,
b) successive **study stages** to which the dimensions apply, with a stage being defined as a well-defined period within the continuum of a study, and
c) quality **promoters**, with a promotor being defined as set of factors that may enhance all listed quality dimensions at a research institution.

	**Research stage 1**	**Research stage 2**	**Research stage …**
**Quality dimension 1**	- Item 1<br>- Item 2<br>- Item 3	…	
**Quality dimension 2**	…	…	
**Quality dimension …**			

Figure 1. Initial framework matrix

Second, quality definitions, criteria, or themes acquired through our systematic search were first coded into quality items (i.e. single aspects of quality) and then thematically grouped into overarching quality dimensions. We identified a total of six quality dimensions and five successive temporal research stages, resulting in a 5x6 matrix. Two groups of items, those belonging to infrastructural aspects and the sustainability aspect of educating junior researchers, did not fit within one dimension or temporal stage and were included as quality promoters. We subjected this initial framework to iterative consultation about comprehensiveness and subsequent editing by the authors and affiliated interested academics until we reached internal consensus.

Delphi process

We subjected the framework to a modified online Delphi process consisting of three successive stages:

i) Identification and invitation of stakeholder representatives
ii) Delphi-rounds 1 and 2: Identification of any additional quality item that we had not yet considered, and establishing broad consensus across stakeholders on the overall framework structure
iii) Delphi rounds 3 and 4: Seeking agreement on a more refined framework including specific quality questions and descriptive examples, with a focus on operationalization in the Swiss academic setting

i) Identification of stakeholder representatives

To allow for broad inclusion of perspectives, we considered the same seven stakeholder groups to be relevant as in the systematic review[1]: (1) patient organizations and representatives, (2) academic national research institutions/initiatives, clinical investigators, academic clinical trial units, methodological researchers (3) national and supranational governmental bodies, (4) regulatory agencies, (5) ethics committees, (6) the pharmaceutical industry and contract research organizations, and (7) funding agencies.

Our team, with help from affiliated collaborators, and by word of mouth among the related networks (e.g. European Patient Academy on Therapeutic Innovation (EUPATI) for patient representatives) identified potential stakeholder representatives from 16 countries. We recruited participants on the basis of awareness of quality issues related to clinical research and ability to provide feedback within a specified time window.

ii) Delphi-rounds 1 and 2

In round one and two, 109 survey participants from 16 countries were invited through the survey software SurveyMonkey© (www.surveymonkey.net) to provide their comments on the overall suitability and the comprehensiveness of the proposed framework structure, and the individual items to be included. These two Delphi-rounds aimed at (i) identifying any additional quality item that we had not yet considered, and (ii) establishing broad consensus across stakeholders on the overall framework structure. Consensus was pre-defined as an agreement of 80% or higher. Only stakeholders who responded in round one were invited to participate in the following round. After each round, we shared with respondents a summary of the adaptations made based on their suggestions in the previous round and asked for their agreement or further improvements and suggestions on structure and content. Of the 109 invited, 58 (53%) participants provided suggestions or comments in the first round; and 45/109 completed both rounds (see main manuscript, Table 1). In each round, we sent two reminders via SurveyMonkey©.

iii) Delphi-rounds 3 and 4

Seeking consensus on how to operationalize the framework structure in the Swiss academic setting, for Delphi rounds three and four, we invited additional 33 stakeholder representatives from Switzerland, particularly academics (see main manuscript, Table 2). In particular, we invited representatives (board members and executive directors) of all six Swiss Clinical Trial Units at University hospitals and members of the executive committee and the Quality Working Group at the Swiss Clinical Trial Organization. For this round, the previous “quality items” were rephrased as “main quality question” accompanied by descriptive examples in order to allow operationalization of the framework. We asked for the agreement (yes/no) on the adapted framework structure, content, and wording of main quality questions and corresponding examples and allowed for free text comments on the suitability, the comprehensiveness, and the completeness of dimensions and items for each research stage. In round four, we additionally provided respondents with all anonymized comments, a response by the authors to each comment, and the overall agreement score on framework structure and main quality questions. In round four, “main” quality questions were rephrased as “specific” quality questions. Participants were again asked for their agreement on structure and content of the framework and were allowed to suggest specific adaptations to the framework using a shareable, but anonymized, googledocs.com (https://docs.google.com) format. Final adaptations to the framework were made by the authors through iterative discussion and shared with the Delphi participants. After round four, an agreement of over 80% was reached for the structure as well as the main quality questions in each research stage (see main manuscript, Table 2). In each round, we sent minimum two email reminders.

References

1. von Niederhäusern B, Schandelmaier S, Mi Bonde M, et al. Towards the development of a comprehensive framework: Qualitative systematic survey of definitions of clinical research quality. *PLoS ONE* 2017; **12**(7): e0180635.
2. Gale NK, Heath G, Cameron E, Rashid S, Redwood S. Using the framework method for the analysis of qualitative data in multi-disciplinary health research. *BMC Med Res Methodol* 2013; **13**: 117.

SurveyMonkey© Questionnaires (Round 1-3) and Email Questionnaire (Round 4)

A Framework for Quality of Clinical Research

Welcome to our survey on the quality of clinical research - Round 1

Thank you for your interest in participating in our survey.

We have developed an initial quality framework for clinical research based on extensive internet search, a systematic literature review, as well as existing quality assessment tools.

Your involvement would be to answer three rounds of survey questions of which each round should only take you 10-20 minutes.

1) For the first round, we ask you to complete a short survey regarding the overall structure and suitability of the framework.

2) For the second round, we will present you with the adapted framework from round 1 and ask you to complete a survey regarding the relative importance of the dimensions within the framework.

3) For the third round, we will present the edited list of dimensions and ask you to repeat your assessment to find final expert consensus.

A Framework for Quality of Clinical Research

Stakeholder affiliation

1. Please chose which stakeholder group you mainly represent in this survey

- Patient group / representative
- Academic research / Initiatives
- Pharmaceutical Industry / CROs
- Ethics committee / IRB
- Governmental body / Jurisdiction
- Regulatory body / agency / HTA
- Funding Agency

A Framework for Quality of Clinical Research

We will now show you, step by step, the structure of our framework. At the end of this page, you will be asked to give your opinion on the suitability of its individual components.

Step 1:
For high-quality research, infrastructure needs to be in place and feasibility of research needs to be checked before study conduct.

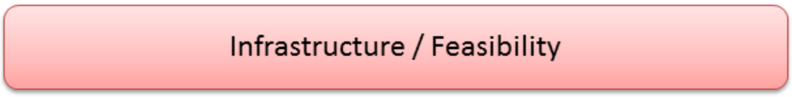

Step 2:
Quality of research needs to be ensured across all study phases (planning, conduct, dissemination).

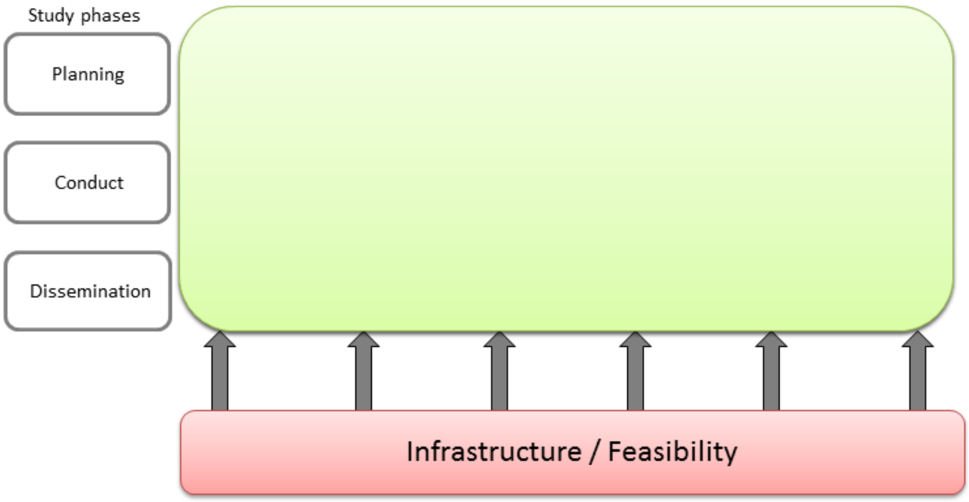

Step 3:

6 distinct quality dimensions across all study phases are crucial to ensure that clinical research is:

- absent of bias/ high in internal validity
- high in precision
- high in external validity
- innovative & relevant
- ethical and safe for study participants
- transparent & data is accessible

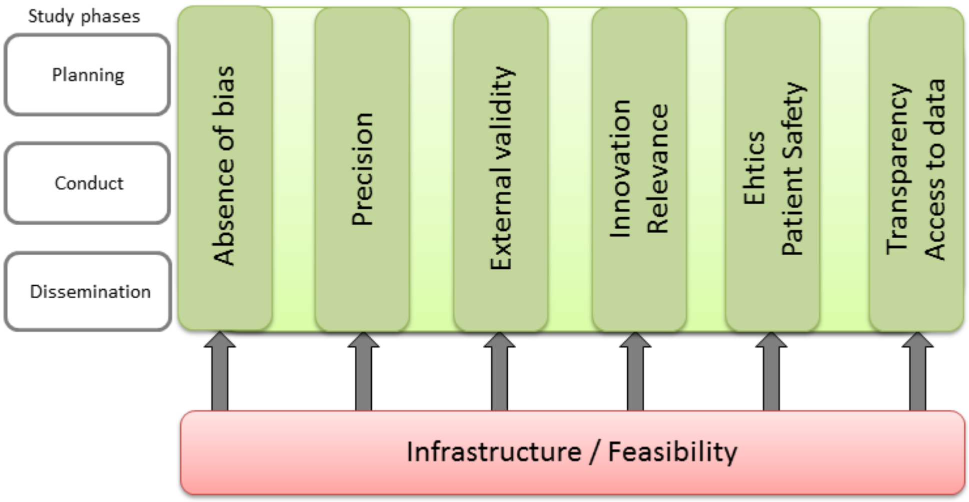

Step 4:

If all 6 dimensions are of high quality, a clinical study may serve to educate junior/fellow researchers and have a sustainable positive impact on the overall clinical research environment.

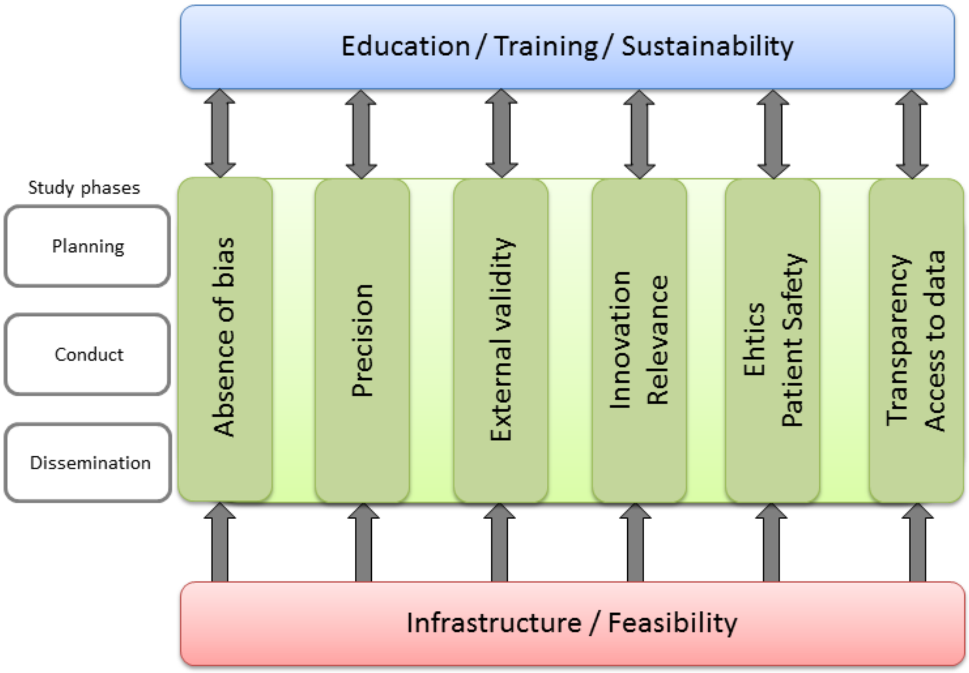

2. In your opinion, does the overall framework encompass the crucial aspects of quality in clinical research?

- ◯ Yes
- ◯ No (please specify)

3. Do you agree on the 8 quality dimensions (infrastructure/feasibility; absence of bias; precision; external validity; innovation/relevance; ethics/patient safety; transparency/access to data; education/training/sustainability)?

○ Yes

○ No (please specify)

4. Do you agree on the 3 study phases (planning, conduct, dissemination)?

○ Yes

○ No (please specify)

5. Do you have any other comments, questions, or concerns?

Thank You and See You Soon!

Thank you very much for participating in the first part of our survey. Your answers are of high value to us.

In the next survey round, we will ask you to rate the relevance of the quality dimensions.

A Framework for Quality of Clinical Research

Welcome to our survey on the quality of clinical research - Round 2

Thank you for your interest in further participating in our survey!

1) For the first round, we asked you to complete a short survey regarding the overall structure and suitability of the framework.

2) For the second round, we now present you with the adapted framework based on round 1 and the corresponding quality items. At the end, we ask you to rank the relative importance of the dimensions within the framework.

3) For the third round, we will present the pre-final version of the framework including quality items and ask you to repeat your assessment to find final expert consensus.

Stakeholder affiliation

1. Please choose which stakeholder group you mainly represent in this survey

- ◯ Patient group / representative
- ◯ Academic research / Initiatives
- ◯ Pharmaceutical Industry / CROs
- ◯ Ethics committee / IRB
- ◯ Governmental body / Jurisdiction
- ◯ Regulatory body / agency / HTA
- ◯ Funding Agency

In round 2, we will:

1. Show you the overall structure of the framework which has been adapted based on the results of round 1.

2. Present you with a list of representative quality items for each quality dimension. These items serve to illustrate the content and scope of an individual quality dimension but are not exhaustive.

3. Ask you to rate the subjective relative importance of the 5 quality dimensions.

Quality framework structure

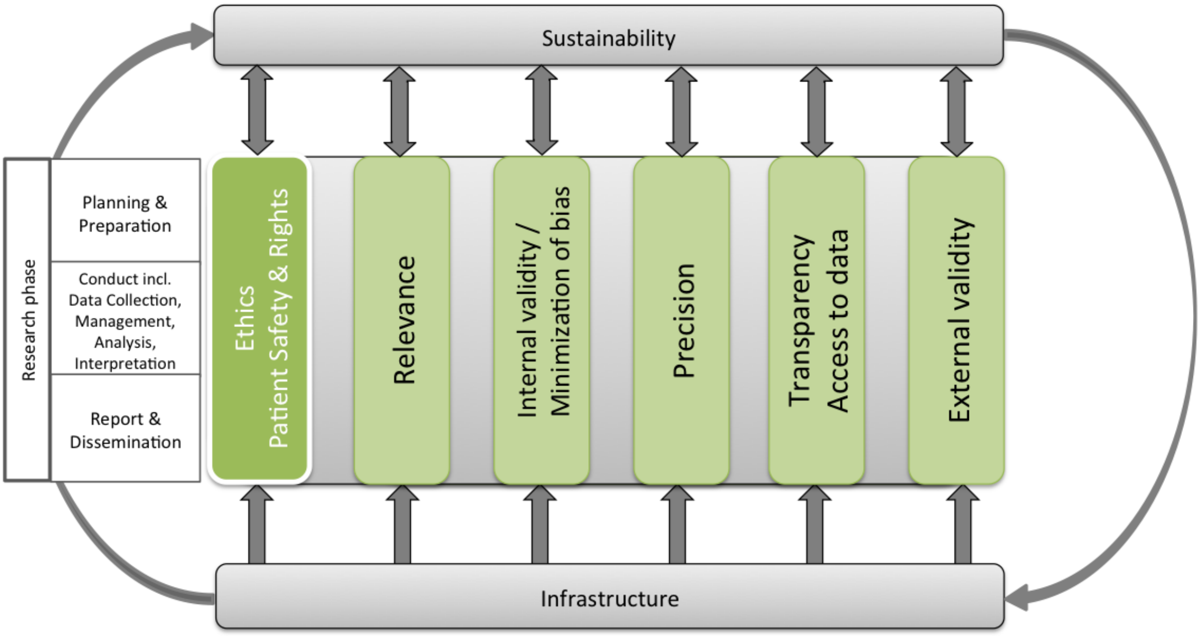

Based on the highly-valued comments from the first survey round, we made the following major adaptions to the quality framework:

- We emphasize that the ethical conduct and protection of patients' safety and rights dimension is the cornerstone of research and non-negotiable/-gradable by spatially separating it from the other 5 quality dimensions that are gradable. We further placed «ethics/patient safety & rights» at the very beginning of our quality dimensions to highlight its importance.
- Several experts stated that clinical research does not necessarily need to be «innovative» in order to be of relevance (e.g. importance of valuable replication of study results). We therefore removed «innovation» and renamed the dimension as «relevance».
- «Absence of bias» as a quality dimension has been changed to «minimization of bias».
- The framework terminology did not seem to be self-explaining. We clarify that there are 6 quality dimensions embedded in/surrounded by and interacting with a research environment that a) consists of an established infrastructure including well-trained personnel and functional facilities, and b) uses ongoing clinical research efficiently for training purposes of young investigators and other study personnel in order to ensure sustainability of an effective infrastructure.
- Several experts mentioned the importance of an analysis phase. We now explicitly mention data analysis within our conduct phase. For quality items relating to the analysis phase, please take a look at the detailed item list below.

Each dimension (column) includes individual items that are sorted by categories reflecting different phases of clinical research. You find these items listed on the next page.

2. Does the adapted structure of the framework make sense to you?

◯ Yes

◯ No

I would like to comment

Quality items

We now present you with a list of representative quality items for each quality dimension. These items serve to illustrate the content and scope of an individual quality dimension but are not exhaustive.

If you would like to comment on these items or make suggestions for items we should add, you can use the comment fields below.

You can also complete the survey without commenting on the quality items.

Ethics / Patient safety & rights: Assurance that patient/participants' safety, rights, and well-being are respected and protected at all times (non-negotiable, conditio sine qua non)

	Quality Dimension
**Study phase**	**Ethics / Patient safety & rights**
**Planning & Preparation**	▪ Adherence to regulations/laws (local, national, international) and guidelines (e.g. GCP, GMP)<br>▪ Thoughtful checking of feasibility (i.e. through pilot study)<br>▪ Approval by ethics committee including informed consent & regulatory agency (as appropriate)<br>▪ No selection towards minorities<br>▪ Independently replicated preclinical data present<br>▪ Clinically meaningful control group (e.g. clinically relevant intervention rather than "no treatment" or "placebo")<br>▪ Beta-testing of procedures and dry-runs of anticipated protocol events
**Conduct incl. Data Collection, Management, Analysis, Interpretation**	▪ Respect for and consideration of patient rights, well-being, dignity & safety throughout conduct of study<br>▪ Informed consent<br>▪ Adequate measurement and reporting of side effects, AEs, SAEs, etc.<br>▪ Protection of subject privacy & confidentiality (during & after trial)
**Report & Dissemination**	▪ Products/interventions made available to subjects after trial (access to treatment, if applicable)<br>▪ Explicit reporting of approval from an IRB/EC<br>▪ Adherence to all regulatory reporting timelines<br>▪ Declaration of conflict of interest (integrity)<br>▪ Inform subjects about trial results/treatment arm

3. Do you have any comments or additions to the above quality items?

Relevance: Reflects the extent to which the research (question) is scientifically or societally beneficial (i.e. leads to improved decision-making in health care)

	Quality Dimension
**Study phase**	**Relevance**
**Planning & Preparation**	▪ Add-on value to already existing evidence (i.e. expands or challenges current knowledge, opens additional areas for new research activity)<br>▪ Therapeutic outcome measures/endpoints: clinical (not surrogate), well-defined, pre-specified, valid, reliable, sensitive to important change and measured at appropriate times to enable comprehensive assessment of benefits and harms)<br>▪ Quality of life measured<br>▪ Use of innovative/original methods<br>▪ Assessment of cost – benefit of study
**Conduct incl. Data Collection, Management, Analysis, Interpretation**	▪ Collection of cost data (cost-effectiveness)<br>▪ Conclusive inference about clinically meaningful treatment effect possible
**Report & Dissemination**	▪ Citation indeces/citations in clinical guideline<br>▪ Critical reflection on research findings to guide the directions of future research<br>▪ Reporting of challenges and mistakes to improve future research

4. Do you have any comments or additions to the above quality items?

Internal validity / Minimization of bias: Reflects the extent to which systematic error (bias) is minimized

	Quality Dimension
**Study phase**	**Internal validity / Minimization of bias**
**Planning & Preparation**	▪ Minimization of selection bias (e.g. randomization including allocation concealment)<br>▪ Minimization of performance and detection bias (e.g. - blinding of patients, care-givers, and outcome assessors, endpoint judgements by endpoint committee)<br>▪ Careful planning for unblinding procedures, both intentional & unintentional<br>▪ Minimization of attrition bias (e.g. minimizing losses to follow-up)<br>▪ Careful planning of data collection (e.g. considering all relevant confounders)
**Conduct incl. Data Collection, Management, Analysis, Interpretation**	▪ Accurate data collection and pre-specified analysis<br>▪ Pre-specified subgroup analyses<br>▪ Risk-based monitoring approach<br>▪ Formal techniques to monitor compliance<br>▪ Data analysis using standard, generally accepted software<br>▪ Minimization of confounding and selection bias (e.g. multivariable analysis, intention to treat principle)<br>▪ Avoid conflicts of interest
**Report & Dissemination**	▪ Reporting of all patient-relevant outcomes as planned (no selective reporting)

5. Do you have any comments or additions to the above quality items?

Precision: Reflects the extent to which random error is minimized (i.e. sufficiently narrow confidence intervals are achieved to confirm or reject clinical hypothesis)

	Quality Dimension
**Study phase**	**Precision**
**Planning & Preparation**	▪ Precise estimation of number of eligible patients, consent rate (e.g. through pilot study)<br>▪ Precise estimation of treatment effect and event rate in control group (e.g. comprehensive consideration of previous evidence through systematic review and meta-analysis)<br>▪ Accurate sample size
**Conduct incl. Data Collection, Management, Analysis, Interpretation**	▪ Precise and reliable outcome measurements<br>▪ Enrollment monitoring and adaptions if needed<br>▪ Systematic data recording & collection<br>▪ Formal techniques to monitor/assess patient compliance
**Report & Dissemination**	▪ Reporting of results with confidence intervals on an absolute and relative scale

6. Do you have any comments or additions to the above quality items?

Transparency / Access to data: Reflects the extent to which study planning, conduct, data and results are transparent to and accessible for the scientific community/public

	Quality Dimension
Study phase	Transparency / Access to data
Planning & Preparation	▪ Publication of protocol<br>▪ Registration in publicly accessible database/registry (making objectives and methods transparent early on)<br>▪ Protocol design & description in accordance with SPIRIT<br>▪ Peer-review of protocol (e.g. for funding/grants)<br>▪ A plan for dealing with “partial success”
Conduct incl. Data Collection, Management, Analysis, Interpretation	▪ Compliance with protocol, otherwise amendments<br>▪ Compliance with guidelines<br>▪ Detailed methods disclosed to enable reproducibility<br>▪ Conduct of internal audits and truthful reporting<br>▪ External and independent DSMB (e.g. for interim analyses)
Report & Dissemination	▪ Maximising dissemination through open access<br>▪ No bias towards results of study<br>▪ Independent and national/international peer review<br>▪ Avoiding “spin” in report and interpretation of results<br>▪ Record-keeping<br>▪ Adherence to reporting guidelines(e.g. CONSORT, STROBE, PRISMA) to facilitate critical appraisal and reproducibility

7. Do you have any comments or additions to the above quality items?

External validity: Reflects the extent to which study results are applicable and generalisable to the wider patient population in a real-world setting

	Quality Dimension
Study phase	External validity
Planning & Preparation	▪ Incorporation of patient preferences/rationale in design (min. burden, max. benefit)<br>▪ Wider/ less restrictive eligibility criteria/inclusion & exclusion criteria (for rapid accrual, broader generalization, pragmatic study)<br>▪ Incorporation of patient advocates in the design and recruitment<br>▪ Subjects representative of patients who would use the drug/intervention
Conduct incl. Data Collection, Management, Analysis, Interpretation	▪ Patient follow-up close to clinical practice<br>▪ Study protocol/procedures well adapted to routine clinical practice
Report & Dissemination	▪ Impact on guideline recommendations<br>▪ Impact on clinical practice/future decisions<br>▪ Report proportion of patients who declined randomization<br>▪ Clear reporting of inclusion and exclusion criteria and characteristics of included patients<br>▪ Detailed methods disclosed to enable reproducibility

8. Do you have any comments or additions to the above quality items?

Infrastructure & Sustainability items

Below you find the items belonging to the research environment in which the quality dimensions are embedded. You do not need to comment in order to complete the survey.

Infrastructure: Includes well-trained personnel and functional facilities

Infrastructure

- Established infrastructure:
- Qualified (GCP mandatory), experienced, and committed study personnel and investigator (proven through past research)
- Involvement of expert statisticians and/or clinical trial units, professional data management
- Inter-/Multidisciplinary collaboration and involvement in clinical trial planning and process
- Good cooperation & communication between involved staff, sponsor, contractors, and site
- Ongoing GCP, protocol, and SOP training for all key clinical staff
- QMS & SOPs in place and followed (e.g. regular audits, personnel roles and responsibilities, training, policies & procedures, QA, document management, record retention, and reporting, corrective and preventive action)
- Competent and effective IT support
- Qualified and experienced personnel in charge of a robust budget plan and securement of funding

9. Do you have any comments or additions to the above items?

Sustainability: Efficient use of ongoing clinical research for training purposes of young investigators and other study personnel in order to ensure sustainability of an effective infrastructure

Sustainability

- Involvement of doctoral students/junior researchers under supervision of senior researchers in study design, planning, protocol
- Hands-on experience of doctoral students/junior researcher/young clinicians in study conduct (e.g. data collection, management, analysis
- Involvement in scientific writing & presentations (e.g. publications, conferences, symposia etc.)
- Community & provider education and outreach; facilitation of two-way communication with diverse populations and community groups
- Knowledge transfer & exchange
- Publicly available doctoral/master theses

10. Do you have any comments or additions to the above items?

Rank relative importance of quality dimensions

We now ask you to rank the 5 quality dimensions (ethics / patient safety & rights excluded, as non-negotiable) based on your subjective judgement according to their relative importance in clinical research.

You can rank the dimensions from 1-5 or apply the same ranks to two or more dimensions at the same time, e.g.:

Relevance = 1, internal validity=1
External validity = 2
Transparency / Access to data = 3, Precision = 3

11. Please rank the relative importance of the 5 quality dimensions according to your subjective judgement from 1 (most important) to 5 (least important):

	1	2	3	4	5
Relevance	○	○	○	○	○
Internal validity / Minimization of bias	○	○	○	○	○
Precision	○	○	○	○	○
Transparency / Access to data	○	○	○	○	○
External validity	○	○	○	○	○

12. Do you have any other comments, questions, or concerns?

Thank You and See You Soon!

Thank you very much for participating in the second part of our survey. Your answers are of high value to us.

In the next and final survey round, we will ask you to assess the final framework in order to find final consensus.

A Framework for Quality of Clinical Research

Background & Aim

Welcome and thank you for your interest in participating in our survey!

Context

Based on the Lancet Series "Increasing Value, Reducing Waste" in 2014, we have been working towards consensus on a comprehensive framework for the quality of clinical research across stakeholder groups. This will allow setting the ground for a standardized assessment of quality and therefore support the discussion on how to "increase value" internationally.

You will be presented with a framework that has been adapted over two rounds of feedback from different stakeholders around the globe (>40 experts). It is now structured according to the five research stages in a clinical study (i.e. concept, planning, conduct, analysis, and dissemination) and contains quality items for six quality dimensions (ethics, relevance/patient centeredness, minimization of bias, precision, transparency/access to data, and generalizability), which allows for its operationalization (e.g. as a quality checklist).

Aim of this survey:

We ask you to assess the validity (i.e. suitability) of the structure and content of the framework in order to find expert consensus.

Next step:

Using the Delphi method, we will provide each of you with the anonymized comments made by your colleagues and the overall agreement regarding content and structure in a last final round following this survey.

Deadline:

Thank you for completing our survey by **February 28, 2017.**

Questions?

We try to keep the information here as crisp as possible for you. If you would like to learn more about the background and methodology of this project, please do not hesitate to contact Belinda von Niederhäusern belinda.vonniederhausern@usb.ch

Stakeholder affiliation

1. Please choose which stakeholder group you mainly represent in this survey

- ◯ Patient group / representative
- ◯ Academic research / Initiatives
- ◯ Pharmaceutical Industry / CROs
- ◯ Ethics committee / IRB
- ◯ Governmental body / Jurisdiction
- ◯ Regulatory body / agency / HTA
- ◯ Funding Agency

Survey structure

In this round, we will ask for your opinion on:

1. The adapted overall structure of the framework

2. The adapted content of the framework, i.e. quality questions and examples per study stage and quality dimension

1. Quality framework structure

The quality framework consists of a total of six quality dimensions (ethics, relevance/patient centeredness, minimization of bias, precision, transparency/access to data, and generalisability) that are embedded in and interacting with a research environment that

1. consists of an established infrastructure including well-trained personnel and functional facilities, and
2. uses ongoing clinical research efficiently for training purposes of young investigators and other study personnel in order to ensure sustainability of an effective infrastructure.

Importantly, all quality dimensions span the entire research continuum (i.e. all research stages). The first quality dimension, "Ethics", is non-negotiable. Based on the comments from the last round, we made the following major adaptions to the quality framework:

- We changed research "phases" to research "stages" in order to avoid confusion with the terminology for study phases I-IV
- We added two distinct temporal research stages: 1) Concept and 2) Analysis & Interpretation
- We added "Patient Centeredness" to the "Relevance" dimension
- We extended "Sustainability" to "Sustainability/Education"

Framework for Quality of Clinical Research: Structure

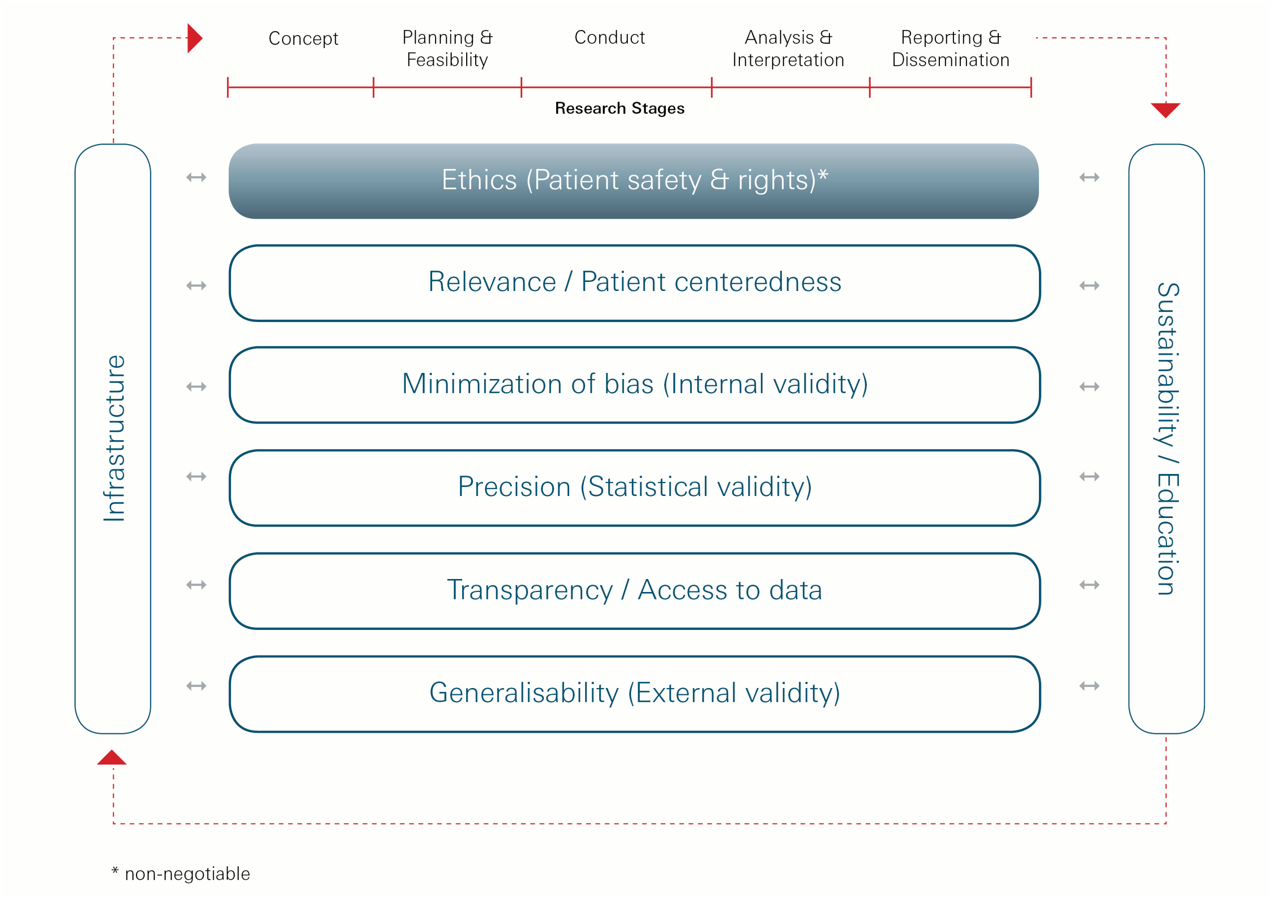

*** 2. Do you agree with the overall framework structure? Do you think it makes sense?**

- ◯ Yes
- ◯ No

3. Do you have any comments or suggestions on the structure of the framework? (particularly relevant if you do not agree with the proposed structure)

2. Framework Content

We will now present you with the content of the framework, i.e. a list of representative quality items for each quality dimensions, sorted by research stage I-V (concept, planning & feasibility, conduct, analysis & interpretation, and reporting & dissemination), "infrastructure" and "sustainability/education".

We divided the quality items into more general "main questions" and detailed "examples" clarifying the content of these questions. These items serve to illustrate the content and scope of an individual quality dimension but are not exhaustive.

We will now ask you whether you do or do not agree on these quality items. If you do not agree, you may add comments and suggestions for changes in the comment field.

2. Framework Content - Study Stage I

Study Stage I: Concept
Milestone: Research question including study type defined and viable

Dimension	Main question	Examples
Ethics (Patient rights & safety)	Can the research question be answered in the given setting?	Based on a rough estimate of required sample size, are enough potential study participants/ patients available in the given setting to answer the research question?
		Based on a rough budget estimate, is it feasible to answer the research question with a specified study type?
	Does study consider equity appropriately?	Are subjects selected so that :
		stigmatized and vulnerable individuals are not targeted for risky research?
		socially powerful individuals are not favored for potentially beneficial research?
Relevance / Patient centeredness	Is significant add-on value to already existing evidence given, taking into consideration burden of disease and anticipated benefit of treatment?	Are uncertainties in existing evidence identified and discussed in a systematic review?
		Does research:
		Expand or challenge current knowledge?
		Open additional areas for new research activity?
		Justify replication of existing evidence, if applicable?
	Are patient representatives/ advocates and their values and preferences involved in the development of the research question?	
	Are outcome measures patient-relevant, well-defined, pre-specified, valid, reliable and measured at appropriate times?	Are outcomes:
		patient-relevant (judicious use of surrogate endpoints)?
		well-defined (upfront)?
		valid (measure what they intend to measure)?
		reliable(stable and consistent when repeatedly measured) ?
		sensitive to important change?
		measured at appropriate times?
Minimization of bias (internal validity)	Is the selected study type/design appropriate to minimize bias?	Is the study randomized or, if not sensible, appropriately controlled for confounding?
Precision (statistical validity)	N/A	
Transparency / Access to data	Is the research question clearly specified (including applicable PICO elements)?	Is each component of PICO clearly defined i.e. :
		Patient population to be recruited in the study
		Intervention to be assessed,
		Control intervention as comparator,
		Outcomes to be measured?
Generalizability (external validity)	Are planned study participants representative of patients who would use the drug/intervention/diagnostic test in a real-life setting?	Are unnecessary restrictions through inclusion/ exclusion criteria avoided (to facilitate rapid accrual, broader generalization, pragmatic study conduct)?
		Is the control group adequate given current evidence and clinical practice (e.g. “standard of care” rather than “no treatment”)?

*** 4. Do you agree on the <u>main quality questions</u> (in grey)?**

- ◯ Yes
- ◯ No

5. If <u>no</u>, what are your suggestions for improvement of the above table?

Study Stage II: Planning & Feasibility
Milestone: Protocol developed and approved by regulatory bodies

Dimension	Main question	Examples
**Ethics (Patient safety & rights)**	Does study adhere to applicable national and international regulations and laws?	Are study documents (e.g. protocol, patient information etc.) written in accordance with applicable national (and international, if applicable) regulations/laws?
		Are informed consent documents written in lay language and easily understandable for study participants?
		Has approval been obtained from ethics committee?
		Has approval been obtained from regulatory agency (if applicable)?
	Has feasibility been checked thoughtfully based on existing evidence?	Is valid and robust preclinical data present (if applicable)?
		Has a pilot study been considered?
		Are recruitment assumptions realistic (e.g. empirical data from electronic health records or from pilot study present)?
		Have national/ international study registries been checked for studies that could interfere with the planned study?
		Do anticipated study costs (preparation, conduct, analysis, dissemination) match with available budget?
		Is study cost data related to planning, conduct, analysis, and dissemination planned to be collected (if applicable)?
	Is collection, documentation, and reporting of Adverse Events / Serious Adverse Events according to the applicable regulations planned and specified in the protocol?	
**Relevance / Patient centeredness**	Is knowledge transfer/use (e.g. plans for inclusion of results in clinical guidelines) planned?	Are relevant guideline groups identified and contact established?
		Are patient representatives involved in protocol development?
	Is statistical analysis pre-specified?	Are outcomes, datasets, subgroups, handling of missing data, etc., pre-specified?
	Is trial monitoring considered and documented in a monitoring plan?	
	Is data management planned and documented in a data management plan?	
	Is minimization of bias planned for according to the research question and study design?	Exemplary items according to study type:
		Please also refer to Cochrane Risk of Bias tool for RCTs [1] for full list of items.
		Is randomization adequate and concealed?

	Randomized Controlled Trials	Are (known) prognostic factors distributed equally (i.e. are groups prognostically balanced at the start of the trial)?
		Is blinding of patients and/or care-givers adequate?
		Are concomitant interventions documented?
		Is blinding of outcome assessors adequate?
		Are plans to minimize losses to follow up present?
		Are plans to analyze study participants in groups as randomized present?
**Minimization of bias (internal validity)**	Observational studies (incl. cohort studies)	Please also refer to ROBINS-I tool [2] for full list of items.
		Is collection of data carefully planned, i.e. are all relevant confounders considered and measured?
		Are all study participants selected or recruited from the same or similar populations (incl. the same time period)?
		Do the study participants represent the cases originated in the community? (e.g. due to issues with health-care access)
		Are inclusion and exclusion criteria pre-specified and applied uniformly to all study participants?
		Are plans to minimize losses to follow-up present?
		Is timeframe sufficient so that one can reasonably expect to see an association between exposure and outcome if it existed?
		For exposures that can vary in amount or level, does the study examine different levels of the exposure as related to the outcome (e.g. categories, or exposure measured as continuous variable)?
		Is exposure measured more than once over time?
	Diagnostic accuracy studies	Please also refer to QUADAS-2 Risk of Bias tool [3] for full list of items.
		Is there an independent, blind comparison between index test and an appropriate gold standard of diagnosis?
		Is the diagnostic test evaluated in a representative, and ideally full spectrum of study participants/ patients (like those in whom it would be used in practice, spectrum ranging from mild to severe, and early to late cases of target disorder)?
		Is a reference standard applied regardless of the index test results (ideally both index test and reference standard should be carried out on all study participants/ patients)?
		If no, is it planned to follow up study participants/ patients for an appropriate period of time (dependent on disease in question) to see if they are truly negative?
**Precision (statistical validity)**	Are expected treatment effects and event rates in intervention and control groups realistic and estimated based on empirical evidence?	Is number of eligible study participants/ patients precisely estimated?
		Is consent rate precisely estimated?
		Are treatment effects and/or event rates estimated in both intervention and control groups?
		If yes, are they based on evidence such as systematic literature reviews, meta-analysis?
		Is rationale for non-inferiority / equivalence design provided (if applicable)?
		Is rationale for maximum clinically acceptable difference (equivalence margins) provided (if applicable)?
		Is sample size realistically estimated and clearly described (incl. assumed treatment effects, referen-

	Is sample size clearly justified to measure expected impact?	ces for estimates, power, alpha error, and expected losses to follow-up)?
		Is rationale for sample size given if not derived statistically?
	Are recruitment procedures and recruitment monitoring planned?	
**Transparency / Access to data**	Is the protocol in accordance with SPIRIT-guideline?	Please also refer to the SPIRIT tool [4] for full list of items.
		Is protocol peer-reviewed?
		Is full trial protocol accessible and published?
		Is study registered in publicly accessible database / registry?
		Does protocol state a plan on how to deal with study publication in case target sample size could not be achieved/study had to be discontinued prematurely?
**Generalizability (external validity)**	Are study procedures well adapted to routine clinical practice?	Is standard of care/current practice clearly defined?
		Are realistic interventions applied which are carried out by physicians in everyday practice?
		Is patient-follow up close to clinical practice?

[1] Higgins JP, Altman DG, Gotzsche PC, Juni P, Moher D, Oxman AD, et al. The Cochrane Collaboration's tool for assessing risk of bias in randomised trials. Bmj. 2011;343:d5928.
[2] Sterne JA, Hernan MA, Reeves BC, Savovic J, Berkman ND, Viswanathan M, et al. ROBINS-I: a tool for assessing risk of bias in non-randomised studies of interventions. Bmj. 2016;355:i4919.
[3] Whiting PF, Rutjes AW, Westwood ME, Mallett S, Deeks JJ, Reitsma JB, et al. QUADAS-2: a revised tool for the quality assessment of diagnostic accuracy studies. Ann Intern Med. 2011;155:529-36.
[4] Chan AW, Tetzlaff JM, Gotzsche PC, Altman DG, Mann H, Berlin JA, et al. SPIRIT 2013 explanation and elaboration: guidance for protocols of clinical trials. Bmj. 2013;346:e7586.

* 6. Do you agree on the main quality questions (in grey)?

- ◯ Yes
- ◯ No

7. If no, what are your suggestions for improvement of the above table?

[ ]

Study Stage III: Conduct
Milestone: Last patient last visit

Dimension	Main question	Examples
**Ethics (Patient safety & rights)**	Is respect for and consideration of patient rights, well-being, dignity & safety throughout conduct of study guaranteed?	Are study participants respected at all times, i.e.:
		Is withdrawal from study at any time explicitly permitted?
		Are study participants informed of newly discovered risks?
		Are study participants informed about purpose of research, its procedures and potential risks, benefits and alternatives, so that they can make a voluntary decision?
		Are side effects / AEs/ SAEs, SUSARs etc. monitored and reported to the ethics committee within required timeframes?
		Is study participants' privacy and confidentiality ensured during (and after) trial, e.g. through appropriate coding?
**Relevance / Patient centeredness**	Are there any measures in place to assure study participants' participation and cooperation throughout conduct of study (e.g. incentives, phone calls, etc.)?	
**Minimization of bias (internal validity)**	Is data systematically collected as pre-specified in protocol?	Is data collected as pre-specified in the protocol?
		Are losses to follow-up minimized?
		Are protocol deviations documented, and reported to the respective institutions?
		Are changes in study procedures amended in the protocol?
	Is attrition bias minimized?	Do the reasons for dropping out have an impact on the assessment of compliance, effectiveness or safety?
		Are missing data documented by individual outcomes?
	Is performance bias minimized?	Apart from the allocated treatment, are study groups treated equally (e.g. no additional treatments or tests)?
		If applicable, are study participants and clinicians kept "blind" to which treatment was being received?
	Is monitoring conducted according to the pre-specified monitoring plan?	
**Precision (statistical validity)**	Is enrollment of study participants monitored?	Are formal techniques in place to monitor recruitment centrally and at participating sites?
		Are measures in place to allow timely reaction in case recruitment deviates from expectations?
	Are any formal techniques to monitor/assess protocol compliance of participants and study staff in place?	

	place?	
**Transparency / Access to data**	Is trial conduct transparent to all involved parties?	Are protocol amendments disseminated to appropria-te parties within reporting timelines?
		Are internal or external audits planned, conducted and reported?
		Is an external and independent Data Monitoring Committee present, or reason provided, why it is not needed?
**Generalizability (exter-nal validity)**	Are numbers of participants through different stages of a study documented (patient flow) including reasons for leaving the study before its end?	Is proportion of study participants who declined ran-domization documented?
		Are the reasons for participants leaving the study before its scheduled end documented?

* 8. Do you agree on the main quality questions (in grey)?

- ◯ Yes
- ◯ No

9. If no, what are your suggestions for improvement of the above table?

[ ]

2. Framework Content - Study Stage IV

Study Stage IV: Analysis & Interpretation
Milestone: Study data analyzed and interpreted

Dimension	Main question	Examples
**Ethics (Patient safety & rights)**	N/A	
**Relevance / Patient centeredness**	Is an inference about clinically meaningful treatment effects possible?	
**Minimization of bias (internal validity)**	Is the data analyzed as pre-specified in the protocol?	Is data analyzed as pre-specified in protocol?
		Are post-hoc analyses clearly labelled as such or as exploratory analyses?
		Is data analysis performed using standard, generally accepted software?
		Are data assumptions checked (e.g. normal distribution) as appropriate for planned statistical tests/ modelling?
	Are key confounding variables adjusted for in the analysis (e.g. multivariable analysis)?	
	Is the intention-to-treat principle followed (i.e. study participants were analyzed in groups as randomized) in case of a superiority hypothesis?	
	Are both a per-protocol and an analysis following the intention-to-treat principle conducted in case of a non-inferiority hypothesis?	
	Are results interpreted without "spin"?	
**Precision (statistical validity)**	Is the uncertainty of results through missing outcome data considered in the analysis e.g. through reasonable sensitivity analyses?	
**Transparency / Access to data**	Is the analysis code clearly documented and the analysis process reproducible?	
**Generalizability (external validity)**	N/A	

*** 10. Do you agree on the main quality questions (in grey)?**

- ◯ Yes
- ◯ No

11. If no, what are your suggestions for improvement of the above table?

2. Framework Content - Study Stage V

Study Stage V: Reporting & Dissemination
Milestone: Study archived and published

Dimension	Main question	Examples
**Ethics (Patient safety & rights)**	Was study completion/termination communicated to appropriate parties and documented in registries?	Was study completion/termination reported to ethics committee/regulatory bodies?
		Was study completion/termination appropriately documented in national/international registry?
**Relevance / Patient centeredness**	Did authors critically reflect on research findings (results as well as challenges or mistakes during study conduct) and the implications for future research?	
	Is the study easily available to decision/policy/guideline makers?	Has the study been cited in a clinical guideline?
	Were study participants involved in the reporting of the study?	Were study participants informed about outcome of the study?
		Had patient representatives been involved in reporting of the study, e.g. in writing of lay term summaries?
	Did study participants get access to products/interventions after trial?	
**Minimization of bias (internal validity)**	Were all outcomes and important trial characteristics reported as pre-specified in the protocol (outcome reporting bias prevented)?	Were all patient-relevant outcomes reported as pre-specified in the protocol?
		Were important modifications to the protocol (e.g. premature discontinuation) reported (if applicable)?
**Precision (statistical validity)**	Were absolute and relative treatment effects reported accompanied by confidence intervals?	
	Was the analysis set of participants clearly specified?	Were the actual numbers of recruited, randomized (if applicable), followed-up, and analyzed participants reported for each outcome and for each treatment group (if applicable)?
	Was dissemination of data and study results maximized?	Was dissemination maximized through open access?
		Was anonymized individual participant-level data made available (data sharing)?
		Were study results posted in trial registries?
		Did publication in journals include full protocol and statistical analysis plan?
		Was dissemination maximized through use of alternative media other than medical journals?
		Were resulting doctoral/master theses made publicly available (if applicable)?
	Were reporting guidelines followed to facilitate critical appraisal and reproducibility?	Was reference made to reporting guidelines such as CONSORT (Randomised trials) [1], STROBE (Observational studies) [2], STARD (Diagnostic studies) [3], or PRISMA (Systematic reviews) [4] depending on the respective study design.

		Were detailed methods disclosed in publications (to enable reproducibility)?
**Transparency / Access to data**	Were selective reporting, spin, plagiarism and self-plagiarism avoided and conflicts of interest declared?	Was selective reporting of study results avoided?
		Was plagiarism and self-plagiarism avoided?
		Were the study results independently peer reviewed?
		Was spin avoided in reporting of results?
		Were conflicts of interest declared?
	Was knowledge transfer & exchange fostered?	Was knowledge transfer & exchange fostered through e.g.:
		Community and provider education and outreach
		Community and provider education and outreach
		Knowledge transfer & exchange among clinical research groups
	Were records kept and archived?	
**Generalizability (external validity)**	Did results impact clinical practice?	Did results impact guideline recommendations?
	Were characteristics of included patients clearly reported?	Were characteristics of included patients clearly reported?
		We characteristics of included patients clearly reported?

[1] Altman DG, Schulz KF, Moher D, Egger M, Davidoff F, Elbourne D, et al. The revised CONSORT statement for reporting randomized trials: explanation and elaboration. Ann Intern Med. 2001;134:663-94.
[2] Vandenbroucke JP, von Elm E, Altman DG, Gotzsche PC, Mulrow CD, Pocock SJ, et al. Strengthening the Reporting of Observational Studies in Epidemiology (STROBE): explanation and elaboration. Int J Surg. 2014;12:1500-24.
[3] Cohen JF, Korevaar DA, Altman DG, Bruns DE, Gatsonis CA, Hooft L, et al. STARD 2015 guidelines for reporting diagnostic accuracy studies: explanation and elaboration. BMJ Open. 2016;6:e012799.
[4] Liberati A, Altman DG, Tetzlaff J, Mulrow C, Gotzsche PC, Ioannidis JP, et al. The PRISMA statement for reporting systematic reviews and meta-analyses of studies that evaluate health care interventions: explanation and elaboration. Ann Intern Med. 2009;151:W65-94.

* 12. Do you agree on the <u>main quality questions</u> (in grey)?

○ Yes

○ No

13. If <u>no</u>, what are your suggestions for improvement of the above table?

[ ]

2. Framework Content - Infrastructure

Infrastructure

Is a Quality Management System incl. Standard Operating Procedures (SOPs) in place?	Is all staff continuously trained in applicable SOPs?
	Are there measures in place to control, whether the existing Quality Management System is followed? (i.e. internal audits)
Is a critical mass of well-trained and experienced principal investigators and study staff present?	Has the principal investigator and/or staff been involved in clinical studies before?
Is data systematically collected as pre-specified in protocol?	Is all staff continuously trained in GCP and protocol-related activities?
	Is training (e.g. GCP) of each participating investigator and staff member clearly documented?
	Are roles and responsibilities of each participating investigator and staff member clearly documented?
	Are all involved stakeholders well and adequately informed about study procedures and changes?
Are expert epidemiologists/ methodologists, statisticians, professional data managers, and/or a logistical support unit involved early-on?	Are epidemiologists/methodological specialists involved in development of protocol?
	Are statisticians involved in development of protocol?
	Are data managers involved in the development of the data management plan and the setup of the data management system?
	Is a logistical support unit involved in study planning and/or conduct, e.g. through regulatory affairs experts, study nurses, or project managers?
Are adequate human, material, and equipment resources available for study conduct?	Is dispense, transport, and storage of investigational medicinal product, if applicable, planned?
	Is availability of study-specific materials, hardware, and facilities planned and secured?
	Is a transparent study budget available and approved by experienced personnel, including costs for experts mentioned above?
	Is funding secured through acquisition of competitive money or through collaboration with e.g. industry partners?
Are adequate facilities ensuring data security and privacy in place (incl. competent and effective IT support to facilitate solutions tailored to specific challenges of individual studies)?	Is an electronic database incl. audit trail in place?
	Is patient data coded?
	Is IT support present at site?
Is inter-/multidisciplinary collaboration and involvement in clinical trial planning and conduct fostered?	Have all relevant stakeholders been involved in protocol development and conduct? (e.g. investigators at other trial sites, etc.)
	Is communication between involved staff, sponsor, contractors, and site fostered?
Is it ensured that all studies which are subject to compulsory insurance have insurance at all applicable institutions?	

*** 14. Do you agree on the <u>main quality questions</u> (in grey)?**

- ◯ Yes
- ◯ No

15. If <u>no</u>, what are your suggestions for improvement of the above quality table?

2. Framework Content - Sustainability/Education

Sustainability / Education

Are doctoral students, junior researchers, or young clinicians actively involved in all stages of a clinical study, and reliably supervised/mentored by senior researchers?	Are doctoral students, junior researchers, or young clinicians actively involved in study design, planning, conduct, analysis, interpretation and dissemination of results (e.g. publications, conference presentations)?
	Are doctoral students, junior researchers, or young clinicians actively supervised by senior researchers at all stages of a clinical study?
	Are doctoral students, junior researchers, or young clinicians mentored as to career options in clinical research?
	Are training options and courses in health research methodology available for principal investigators and staff?
	Are doctoral students, junior researchers, or young clinicians mentored to improve awareness about value of clinical research to patients and society as a whole?
	Are processes continuously adapted and improved to changes, developments, issues, and conditions during research continuum (quality by design)?

*** 16. Do you agree on the main quality questions (in grey)?**

- ◯ Yes
- ◯ No

17. If no, what are your suggestions for improvement of the above table?

Thank You and See You Soon!

Thank you very much for participating in the third part of our survey. Your answers are of high value to us.

In the next and final survey round, we will provide you with the results of this round and the comments made by your colleagues.

18. Do you have any final comments or questions?

E-Mail, Delphi Round 4

Dear Prof. X

You recently participated in our Delphi survey on a framework for the quality of clinical research. Thank you very much!

In order to complete this Delphi process, we would like to get your final opinion on the revised framework. The aim of this last survey round is to maximize consensus across stakeholders.

We have received comments and suggestions from over 50 international experts from seven stakeholder groups. Agreement on the individual research stages is presented below, together with your personal agreement:

**Framework section**	**Overall agreement (yes) (n, %)**	**Your agreement, last round**	**Your agreement, this round**
Overall structure	47/54 (87.0)	Yes	
Stage I: Conceptualization	40/52 (76.9)	Yes	
Stage II: Planning & Feasibility	39/51 (76.5)	Yes	
Stage III: Conduct	43/51 (84.3)	No	
Stage IV: Analysis & Interpretation	43/51 (84.3)	Yes	
Stage V: Reporting & Dissemination	41/51 (80.4)	No	
Quality promoter: Infrastructure	45/51 (88.2)	No	
Quality promoter: Education	44/51 (86.3)	Yes	

Attached, you find the revised framework based on the very constructive and valuable comments we received. We present the changes made to individual dimensions, main questions, or items, in track changes and referred to corresponding comments from you and other experts.

At the end of each document, you find your and all other anonymized comments made by the survey participants, and our replies to those. You can easily identify your comments with your **Participant ID: XX**

We need your final opinion:
In order to complete this Delphi process, please reply to this email using the empty column above "**Your agreement, this round".** Please indicate whether you agree (yes) or do not agree (no) with the revised framework. If you do not agree on a part of the framework, please additionally use the following link to (anonymously) add specific suggestions to the framework:
https://docs.google.com/document/d/1Vk5AkF7FVffeancdCeSjxRAn_EVF_JAVQJ2gpTlqhdo/edit?usp=sharing

Please reply until June 1, 2017.

If you have any questions, please do not hesitate to contact us.

Thank you very much in advance for your time and consideration.
