# Supplementary material for: Academic response to improving value and reducing waste: A comprehensive framework for INcreasing QUality In patient-oriented academic clinical REsearch (INQUIRE)
Source: PLoS Med. 2018 Jun 7;15(6):e1002580. doi: 10.1371/journal.pmed.1002580 (PMC5991651; doi:10.1371/journal.pmed.1002580)
Supplement: S2 Appendix — (DOCX) [file pmed.1002580.s002.docx]

**S2 Appendix**

**Results**

Table A. Agreement scores of Delphi participants on framework structure and content. Only includes those participants who gave an answer to the respective question.

|  | Delphi round 3, n (%) | Delphi round 4, n (%) |
| --- | --- | --- |
| Overall framework structure^1^ | Total : 47/54 (87.0)  Swiss only: 36/40 (90.0) | Total: 53/53 (100)  Swiss only: 40/40 (100) |
| Stage I: Conceptualization, specific questions | Total: 40/52 (76.9)  Swiss only: 30/40 (75.0) | Total: 53/53 (100)  Swiss only: 40/40 (100) |
| Stage II: Planning and feasibility, specific questions | Total: 39/51 (76.5)  Swiss only: 30/39 (76.9) | Total: 52/53 (98.1)  Swiss only: 39/40 (97.5) |
| Stage III: Conduct, specific questions | Total: 43/51 (84.3)  Swiss only: 32/37 (86.5) | Total: 53/53 (100)  Swiss only: 40/40 (100) |
| Stage IV: Analysis and Interpretation, specific questions | Total: 43/51 (84.3)  Swiss only: 32/37 (86.5) | Total: 53/53 (100)  Swiss only: 40/40 (100) |
| Stage V: Reporting and Knowledge Translation, specific questions | Total: 41/ 51 (80.4)  Swiss only: 31/37 (83.8) | Total: 53/53 (100)  Swiss only: 40/40 (100) |
| Quality promoter: Infrastructure | Total: 45/51 (88.2)  Swiss only: 34/37 (91.9) | Total: 53/53 (100)  Swiss only: 40/40 (100) |
| Quality promoter: Education and sustainability | Total: 44/51 (86.3)  Swiss only: 35/37 (94.6) | Total: 51/53 (96.2)  Swiss only: 38/40 (95.0) |

^1^Agreement score on overall framework structure in round 1: 26/49 (53.1%); in round 2: 33/34 (97.1%).

**Detailed description of quality dimensions**

**Protection of patients’ safety and rights**

The first quality dimension, *protection of patients’ safety and rights*, represents the cornerstone of research and is therefore a conditio-sine-qua-non dimension in our framework. It assures that participants’ safety, rights, and well-being are respected and protected at all times.

Participants should be informed about the research and provide their voluntary consent, but also have the opportunity to withdraw.^1-3^ Within the context of standard clinical practice and the research protocol, potential benefits to individuals and the society must outweigh the risks^4,5^ and there should be clinical equipoise- the absence of a consensus regarding the comparative merits of the interventions to be tested.^1,6^ During and after the conduct of the study, participants’ rights, safety, and privacy must be protected at all times. Further, study participants should be selected in a fair and equitable manner.^1^ Moreover, the research protocol must be practically feasible. For example, research that could not possibly enroll sufficient participants cannot generate valid scientific knowledge and is thus unethical.^1,4^ Feasibility assessments prior to study start are crucial in order to avoid waste in financial and human resources – and to justify exposing participants to burdens or risks - leading to the many clinical trials that are terminated prematurely.^7-15^

**Relevance, patient centeredness and involvement**

The second quality dimension, *research relevance and patient centeredness and involvement* reflects the extent to which the research question is scientifically and societally beneficial (i.e. leads to improved decision-making in health care) and involves patient values and preferences at all stages.

Relevant research should build on what is already known, preceded by systematic reviews.^16,17^ Further, it should address a question leading to clinically relevant information gain^18,19^ avoiding subjective approaches that may be unduly influenced by special interest groups.^20^ Institutions should reward rigorous replication of previous work in order to battle the low rate of confirmation.^21^ Ideally, this is incorporated upfront in designing the research agenda in a given field in order to avoid multiple necessary replications or redundant meta-analyses combining them.^19,22^ Further, relevant research is patient centered and should be aligned with patient priorities, the utilities patients assign to different problems and outcomes, and how acceptable they find interventions over the period for which they are indicated.^7,23,24^ As suggested by initiatives such as the Patient-Centered Outcomes Research Institute (PCORI)^25^, the James Lind Alliance (www.lindalliance.org), or the INVOLVE Initiative in the UK (www.invo.org.uk), patient values and preferences should be fostered through patient (representative) engagement during all stages of a clinical study. For example, through close collaboration with patient organizations at all stages, adaptations of inclusion and exclusion criteria where necessary, or appropriate dissemination of lay language summaries of study outcomes.

**Minimization of bias (Internal validity)**

The third quality dimension, *minimization of bias – or internal validity*, reflects the extent to which systematic error (bias) is minimized, i.e. through selecting an appropriate study design and pre-specifying analyses.

Minimizing bias and thereby maximizing internal validity is dependent on the chosen study design and has been described to be difficult to avoid.^26^ Established tools such as the Cochrane Risk of Bias tool for RCTs^27^, ROBINS-I for observational research^28^ or QUADAS-2 for diagnostic accuracy studies^29^ provide guidance on how to plan and conduct studies with minimal bias. An effective solution to mitigate self-deception, for example, is blinding, which is applicable to some research contexts. Chosen outcomes should be pre-specified, valid, reliable, measured at appropriate times, and comparable across similar trials.^30-32^ Data collection should then be conducted in accordance with the procedures pre-specified in the protocol. In general, the adoption of appropriate statistical methods^33^, standardized definitions and analyses and stringent thresholds for claiming discoveries success^34^ may decrease false-positive rates. Finally, conflicts of interests should be avoided, or at least transparently reported, in order to avoid spinning of more favorable conclusions due to the involvement of conflicted parties.^35,36^

**Precision**

The fourth quality dimension, *precision*, reflects the extent to which random error is minimized (i.e. sufficiently narrow confidence intervals are achieved to confirm or reject clinical hypothesis), and to what level precision is reported and described in order for readers to be able to judge it.

The development and approval of valid study methods and improvements in study design have been described to improve the precision, and therefore reliability of results.^37^ Then, efforts need to be made to minimize variability of study procedures and measurement error throughout study conduct to guarantee interpretable data and an ethical study conduct.^1^

It is further important that expected treatment effects and event rates in intervention and control groups are realistic, and that estimates are based on empirical evidence. Validated, non-surrogate outcomes should provide clinical insights to claim power.^7,38-40^ Sample sizes should be justified to measure the expected impact, and recruitment should continuously be monitored to ensure successful reach of target sample size.

**Transparency and access to data**

The fifth quality dimension, transparency and access to data, reflects the extent to which study planning, conduct, data collection and presentation of results are transparent to and accessible for the scientific community and the public. It includes the registration of the study in a publicly accessible database, publication of the full study protocol, publication of the study results - independent of their effect size or direction-, and explicitly, encouraging access to the full patient-level data set (data sharing). Furthermore, much waste occurs after publication: from poor access, poor dissemination, and poor uptake of the findings of research.^41^ Publishing in open access journals may reduce this post-publication waste if editorial policies and peer review processes are of high standards.

Reporting, review, publication, dissemination, and post-publication review of research shape its reliability.^35^ There are currently over 300 reporting guidelines to improve and standardize reporting (e.g. as catalogued by the EQUATOR Network, http://www.equator-network.org/) and multiple ideas about how to change dissemination of information.^42^ Yet, studies that obtain positive and novel results are more likely to be published than studies that obtain negative results or report replications of prior results.^26,42-44^ Research should be pre-registered, as promoted by websites such as Open Science Framework (<http://osf.io/>) in order to enhance transparency.^45-48^ Registration has been proposed for many types of research, including observational studies. Reporting of outcomes should be completely consistent with the pre-registered commitments, and avoid adding new ones (see www.COMPare-trials.org).

Further, sharing of data, protocols, materials, and softwares should be promoted as happening in several -omics fields, such as genomics, proteomics, or metabolomics, and may similarly improve the credibility and reproducibility of clinical research studies.^26,49^ The TOP guidelines^67^ promote open practices while an increasing number of journals and funders require open practices (for example, open data), with some offering their researchers free and open access publication.

**Generalizability (External validity)**

The sixth quality dimension, *generalizability – or external validity-*, reflects the extent to which study results are applicable and generalizable to the wider patient population in real life circumstances.

Thus, the characteristics of planned study participants should be representative of patients who would use the intervention after study end. Further, the flow of participants through each stage including the reasons for which patients left the study before its end should be documented and reported, together with the results of pre-specified subgroup analyses of key patient characteristics (e.g. disease severity, age or gender). Treatment effects may be similar in nonparticipants and capturing real-life circumstances is possible by utilizing pragmatic study designs^50^ allowing for greater applicability and consideration of external validity.^51-53^ In 2009, a first tool called the Pragmatic Explanatory Continuum Index Summary (PRECIS) was published to help researchers think more carefully about the impact their design decisions would have on applicability.^54^ In 2015, an improved, validated version of PRECIS was published providing guidance on how to match design decision to how the trial results are intended to be used.^52^

**Description of quality promoters**

**Sustainability and Education**

Examples of good scientific conduct should be used in practice to train early-career researchers, making quality sustainable. In addition, proper training and continuing education of scientists in research methods and statistical literacy are important to train physicians in critical thinking skills instead and evidence-based research instead of simply producing more papers.^55^ Common statistical misperceptions and interpretations could be addressed through improved training.^24,26^ Moreover, methodological best practices are under constant revision and improvement so that senior as well as junior researchers need continuing education, not least because much training of early-career researchers is informal and flows from their supervisors or mentors.^26^ Educational resources should be accessible, easy-to-digest and immediately and effectively applicable to research in order to maximize their use.^26^

**Infrastructure**

In addition to infrastructural support such as space, equipment, or materials, the need for independent methodological support is well established, particularly for clinical trials. Many of them have multidisciplinary steering committees to provide advice and oversee the design and conduct of the trial. Including independent experts in the design, monitoring, analysis or interpretation of research outcomes may not only improve the study, but also mitigate influences such as financial or non-financial conflicts of interests of the investigators.^56,56^ Collaboration across many study sites can- instead of relying on the limited resources of single investigators- facilitate high-powered designs, standardization, and greater potential for testing generalizability across the settings and populations sampled.^26^

**References**

1. Emanuel EJ, Wendler D, Grady C. What makes clinical research ethical? *JAMA* 2000; **283**(20): 2701-11.

2. The Ethics of Consent: Theory and Practice. New York: Oxford University Press; 2009.

3. Miller FG, Wertheimer A. The fair transaction model of informed consent: an alternative to autonomous authorization. *Kennedy Inst Ethics J* 2011; **21**(3): 201-18.

4. Laurence DR. Ethics and Regulation of Clinical Research. *J Med Ethics* 1988; **14**(1): 44-6.

5. World Medical Association declaration of Helsinki. Recommendations guiding physicians in biomedical research involving human subjects. *Jama* 1997; **277**(11): 925-6.

6. Freedman B. Equipoise and the ethics of clinical research. *N Engl J Med* 1987; **317**(3): 141-5.

7. Ioannidis JP. Why Most Clinical Research Is Not Useful. *PLoS Med* 2016; **13**(6): e1002049.

8. Kasenda B, von Elm E, You J, et al. Prevalence, characteristics, and publication of discontinued randomized trials. *JAMA* 2014; **311**(10): 1045-51.

9. Blumle A, Schandelmaier S, Oeller P, Kasenda B, Briel M, von Elm E. Premature Discontinuation of Prospective Clinical Studies Approved by a Research Ethics Committee - A Comparison of Randomised and Non-Randomised Studies. *PLoS ONE* 2016; **11**(10): e0165605.

10. Briel M, Olu KK, von Elm E, et al. A systematic review of discontinued trials suggested that most reasons for recruitment failure were preventable. *J Clin Epidemiol* 2016; **80**: 8-15.

11. Kasenda B, von Elm E, You J, et al. Prevalence, characteristics, and publication of discontinued randomized trials. *Jama* 2014; **311**.

12. Rosenthal R, Kasenda B, Dell-Kuster S, et al. Completion and publication rates of randomized controlled trials in surgery: an empirical study. *Ann Surg* 2015; **262**.

13. Schandelmaier S, Tomonaga Y, Bassler D, et al. Premature Discontinuation of Pediatric Randomized Controlled Trials: A Retrospective Cohort Study. *J Pediatr* 2017; **184**: 209-14 e1.

14. Schandelmaier S, von Elm E, You JJ, et al. Premature Discontinuation of Randomized Trials in Critical and Emergency Care: A Retrospective Cohort Study. *Crit Care Med* 2016; **44**(1): 130-7.

15. Stegert M, Kasenda B, von Elm E, et al. An analysis of protocols and publications suggested that most discontinuations of clinical trials were not based on preplanned interim analyses or stopping rules. *J Clin Epidemiol* 2016; **69**: 152-60.

16. Clarke M, Chalmers I. Discussion sections in reports of controlled trials published in general medical journals: islands in search of continents? *Jama* 1998; **280**(3): 280-2.

17. Clarke M, Hopewell S, Chalmers I. Reports of clinical trials should begin and end with up-to-date systematic reviews of other relevant evidence: a status report. *J R Soc Med* 2007; **100**(4): 187-90.

18. Evangelou E, Siontis KC, Pfeiffer T, Ioannidis JP. Perceived information gain from randomized trials correlates with publication in high-impact factor journals. *J Clin Epidemiol* 2012; **65**(12): 1274-81.

19. Chalmers I, Bracken MB, Djulbegovic B, et al. How to increase value and reduce waste when research priorities are set. *Lancet* 2014; **383**(9912): 156-65.

20. Minelli C, Baio G. Value of Information: A Tool to Improve Research Prioritization and Reduce Waste. *PLoS Medicine* 2015; **12**(9): e1001882.

21. Ioannidis JP. Why most published research findings are false. *PLoS Med* 2005; **2**(8): e124.

22. Siontis KC, Hernandez-Boussard T, Ioannidis JP. Overlapping meta-analyses on the same topic: survey of published studies. *Bmj* 2013; **347**: f4501.

23. Mullins CD, Vandigo J, Zheng Z, Wicks P. Patient-centeredness in the design of clinical trials. *Value Health* 2014; **17**(4): 471-5.

24. Ioannidis JP, Greenland S, Hlatky MA, et al. Increasing value and reducing waste in research design, conduct, and analysis. *Lancet* 2014; **383**(9912): 166-75.

25. Selby JV, Lipstein SH. PCORI at 3 years--progress, lessons, and plans. *N Engl J Med* 2014; **370**(7): 592-5.

26. Munafò MR, Nosek BA, Bishop DVM, et al. A manifesto for reproducible science. *Nature Human Behaviour* 2017; **1**: 0021.

27. Cochrane Risk of Bias Tool.

28. Sterne JA, Hernan MA, Reeves BC, et al. ROBINS-I: a tool for assessing risk of bias in non-randomised studies of interventions. *Bmj* 2016; **355**: i4919.

29. Whiting PF, Rutjes AW, Westwood ME, et al. QUADAS-2: a revised tool for the quality assessment of diagnostic accuracy studies. *Ann Intern Med* 2011; **155**(8): 529-36.

30. Gorst SL, Gargon E, Clarke M, Smith V, Williamson PR. Choosing Important Health Outcomes for Comparative Effectiveness Research: An Updated Review and Identification of Gaps. *PLoS ONE* 2016; **11**(12): e0168403.

31. Williamson P, Clarke M. The COMET (Core Outcome Measures in Effectiveness Trials) Initiative: Its Role in Improving Cochrane Reviews. *Cochrane Database Syst Rev* 2012; (5): ED000041.

32. Williamson PR, Altman DG, Blazeby JM, et al. Developing core outcome sets for clinical trials: issues to consider. *Trials* 2012; **13**: 132.

33. Nuzzo R. Scientific method: statistical errors. *Nature* 2014; **506**(7487): 150-2.

34. Johnson VE. Revised standards for statistical evidence. *Proc Natl Acad Sci U S A* 2013; **110**(48): 19313-7.

35. Ioannidis JP. How to make more published research true. *PLoS Med* 2014; **11**(10): e1001747.

36. Glasziou P, Altman DG, Bossuyt P, et al. Reducing waste from incomplete or unusable reports of biomedical research. *Lancet* 2014; **383**(9913): 267-76.

37. Poste G. Biospecimens, biomarkers, and burgeoning data: the imperative for more rigorous research standards. *Trends Mol Med* 2012; **18**(12): 717-22.

38. Hopewell S, Dutton S, Yu LM, Chan AW, Altman DG. The quality of reports of randomised trials in 2000 and 2006: comparative study of articles indexed in PubMed. *Bmj* 2010; **340**: c723.

39. Fleming TR, DeMets DL. Surrogate end points in clinical trials: are we being misled? *Ann Intern Med* 1996; **125**(7): 605-13.

40. Ferreira-Gonzalez I, Busse JW, Heels-Ansdell D, et al. Problems with use of composite end points in cardiovascular trials: systematic review of randomised controlled trials. *Bmj* 2007; **334**(7597): 786.

41.Glasziou P. The Role of Open Access in Reducing Waste in Medical Research. *PLoS Med* 2014; **11**(5): e1001651.

42. Chan AW, Song F, Vickers A, et al. Increasing value and reducing waste: addressing inaccessible research. *Lancet* 2014; **383**(9913): 257-66.

43. Song F, Parekh S, Hooper L, et al. Dissemination and publication of research findings: an updated review of related biases. *Health Technol Assess* 2010; **14**(8): iii, ix-xi, 1-193.

44. Chalmers I, Glasziou P. Avoidable waste in the production and reporting of research evidence. *Lancet* 2009; **374**(9683): 86-9.

45. Tse T, Williams RJ, Zarin DA. Update on Registration of Clinical Trials in ClinicalTrials.gov. *Chest* 2009; **136**(1): 304-5.

46. Zarin DA, Tse T. Trust but verify: trial registration and determining fidelity to the protocol. *Ann Intern Med* 2013; **159**(1): 65-7.

47. Zarin DA, Tse T, Williams RJ, Califf RM, Ide NC. The ClinicalTrials.gov results database--update and key issues. *N Engl J Med* 2011; **364**(9): 852-60.

48. Zarin DA, Tse T, Williams RJ, Rajakannan T. Update on Trial Registration 11 Years after the ICMJE Policy Was Established. *N Engl J Med* 2017; **376**(4): 383-91.

49. Doshi P, Goodman SN, Ioannidis JP. Raw data from clinical trials: within reach? *Trends Pharmacol Sci* 2013; **34**(12): 645-7.

50. Tunis SR, Stryer DB, Clancy CM. Practical clinical trials: increasing the value of clinical research for decision making in clinical and health policy. *JAMA* 2003; **290**(12): 1624-32.

51. Rothwell PM. External validity of randomised controlled trials: "to whom do the results of this trial apply?". *Lancet* 2005; **365**(9453): 82-93.

52. Loudon K, Treweek S, Sullivan F, Donnan P, Thorpe KE, Zwarenstein M. The PRECIS-2 tool: designing trials that are fit for purpose. *Bmj* 2015; **350**: h2147.

53. Treweek S, Zwarenstein M. Making trials matter: pragmatic and explanatory trials and the problem of applicability. *Trials* 2009; **10**: 37.

53. Thorpe KE, Zwarenstein M, Oxman AD, et al. A pragmatic-explanatory continuum indicator summary (PRECIS): a tool to help trial designers. *J Clin Epidemiol* 2009; **62**(5): 464-75.

54. Collins FS, Tabak LA. Policy: NIH plans to enhance reproducibility. *Nature* 2014; **505**(7485): 612-3.

55. Panagiotou OA, Ioannidis JP. Primary study authors of significant studies are more likely to believe that a strong association exists in a heterogeneous meta-analysis compared with methodologists. *J Clin Epidemiol* 2012; **65**(7): 740-7.

56. Riaz H, Raza S, Khan MS, Riaz IB, Krasuski RA. Impact of Funding Source on Clinical Trial Results Including Cardiovascular Outcome Trials. *Am J Cardiol* 2015; **116**(12): 1944-7.
