# Supplementary material for: Academic response to improving value and reducing waste: A comprehensive framework for INcreasing QUality In patient-oriented academic clinical REsearch (INQUIRE)
Source: PLoS Med. 2018 Jun 7;15(6):e1002580. doi: 10.1371/journal.pmed.1002580 (PMC5991651; doi:10.1371/journal.pmed.1002580)
Supplement: S3 Appendix — (DOCX) [file pmed.1002580.s003.docx]

**S3 Appendix**

**All versions of the framework**

**Delphi round 1**

| **Infrastructure Sustainability** | | | | | | | | | | |
| --- | --- | --- | --- | --- | --- | --- | --- | --- | --- | --- |
| - Established infrastructure: - Qualified (GCP mandatory), experienced, and committed study personnel and investigator (proven through past research) - Involvement of expert statisticians and/or clinical trial units, professional data management - Inter-/Multidisciplinary collaboration and involvement in clinical trial planning and process - Good cooperation & communication between involved staff, sponsor, contractors, and site - Ongoing GCP, protocol, and SOP training for all key clinical staff - QMS & SOPs in place and followed (e.g.regular audits, personnel roles and responsibilities, training, policies & procedures, QA, document management, record retention, and reporting, corrective and preventive action) - Competent and effective IT support - Qualified and experienced financial personnel (for set-up of a robust budget plan and securement of funding) | | | | | | | | - Involvement of doctoral students/junior researchers under supervision of senior researchers in study design, planning, protocol - Hands-on experience of doctoral students/junior researcher/young clinicians in study conduct (e.g. data collection, management, analysis - Involvement in scientific writing & presentations (e.g. publications, conferences, symposia etc.) - Community & provider education and outreach; facilitation of two-way communication with diverse populations and community groups - Knowledge transfer & exchange - Publicly available doctoral/master theses | | |
| **Study phase** | **Quality Dimension** | **Ethics / Patient safety & rights** | | **Relevance*** | | | **Internal validity (minimization of bias)** | **Precision** | **Transparency / Access to data** | **External validity** |
| **Planning & Preparation** | | | - Adherence to regulations/laws (local, national, international) and guidelines (e.g. GCP, GMP) - Thoughtful checking of feasibility (i.e. through pilot study) - Approval by ethics committee including informed consent & regulatory agency (as appropriate) - No selection towards minorities - Independently replicated preclinical data present - clinically meaningful control group (e.g. clinically relevant intervention rather than “no treatment” or “placebo”) - beta-testing of procedures and dryruns of anticipated protocol events | | - add-on value to already existing evidence (i.e. expands or challenges current knowledge, opens additional areas for new research activity) - therapeutic outcome measures/endpoints: clinical (not surrogate), well-defined, pre-specified, valid, reliable, sensitive to important change and measured at appropriate times to enable comprehensive assessment of benefits and harms) - quality of life measured - Use of innovative/original methods - Assessment of cost – benefit of study | - minimization of selection bias (e.g. randomization including allocation concealment) - minimization of performance and detection bias (e.g. - blinding of patients, care-givers, and outcome assessors, endpoint judgements by endpoint committee) - careful planning for unblinding procedures, both intentional & unintentional - minimization of attrition bias (e.g. minimizing losses to follow-up) - careful planning of data collection (eg. considering all relevant confounders) | | - precise estimation of number of eligible patients, consent rate (eg. through pilot study) - Precise estimation of treatment effect and event rate in control group (eg. comprehensive consideration of previous evidence through systematic review and meta-analysis) - accurate sample size | - publication of protocol - registration in publicly accessible database/registry (making objectives and methods transparent early on) - protocol design & description in accordance with SPIRIT - peer-review of protocol (e.g. for funding/grants) - a plan for dealing with “partial success” | - incorporation of patient preferences/rationale in design (min. burden, max. benefit) - wider/ less restrictive eligibility criteria/inclusion & exclusion criteria (for rapid accrual, broader generalization, pragmatic study) - incorporation of patient advocates in the design and recruitment - subjects representative of patients who would use the drug/intervention |
| **Conduct incl. Data Collection, Management, Analysis, Interpretation** | | | - respect for and consideration of patient rights, well-being, dignity & safety throughout conduct o study - informed consent - adequate measurement and reporting of side effects, AEs, SAEs, etc. - protection of subject privacy & confidentiality (during & after trial) | | - collection of cost data (cost-effectiveness) - conclusive inference about clinically meaningful treatment effect possible | - accurate data collection and pre-specified analysis - pre-specified subgroup analyses - risk-based monitoring approach - Formal techniques to monitor compliance - Data analysis using standard, generally accepted software - minimization of confounding and selection bias (eg. multivariable analysis, intention to treat principle) - avoid conflicts of interest | | - precise and reliable outcome measurements - enrollment monitoring and adaptions if needed - systematic data recording & collection - formal techniques to monitor/assess patient compliance | - compliance with protocol, otherwise amendments - compliance with guidelines - detailed methods disclosed to enable reproducibility - conduct of internal audits and truthful reporting - external and independent DSMB (e.g. for interim analyses) | - patient follow-up close to clinical practice - study protocol/procedures well adapted to routine clinical practice |
| **Report & Dissemination** | | | - products/interventions made available to subjects after trial (access to treatment, if applicable) - explicit reporting of approval from an IRB/EC - adherence to all regulatory reporting timelines - declaration of conflict of interest (integrity) - inform subjects about trial results/treatment arm | | - citation indeces/citations in clinical guideline - critical reflection on research findings to guide the directions of future research - reporting of challenges and mistakes to improve future research | - reporting of all patient-relevant outcomes as planned (no selective reporting) | | - reporting of results with confidence intervals on an absolute and relative scale | - maximising dissemination through open access - no bias towards results of study - independent and national/international peer review - avoiding “spin” in report and interpretation of results - record-keeping - adherence to reporting guidelines(eg. CONSORT, STROBE, PRISMA) to facilitate critical appraisal and reproducability - patents? | - impact on guideline recommendations - impact on clinical practice/future decisions - report proportion of patients who declined randomization - clear reporting of inclusion and exclusion criteria and characteristics of included patients - detailed methods disclosed to enable reproducibility |

*Influenced by values and preferences of community

**Delphi round 2**

|  | **Infrastructure** | | | | | |
| --- | --- | --- | --- | --- | --- | --- |
|  | - GCP-Qualified, experienced, and committed on-site study personnel and investigator (proven through past research) - Early-on involvement of expert epidemiologists/methodologists, statisticians professional data managers, and a logistical support unit (e.g. clinical trial units offering study nurses, regulatory personnel, monitors etc.) - Adequate facilities ensuring data security and privacy - Inter-/Multidisciplinary collaboration and involvement in clinical trial planning and conduct - Good cooperation & communication between involved staff, sponsor, contractors, and site - Ongoing GCP-, protocol-, and SOP-training for all key clinical staff - QMS & SOPs in place, followed and audited ) - Competent and effective IT support to facilitate solutions tailored to specific challenges of individual studies - Qualified and experienced financial personnel (for set-up of a robust budget plan and securement of funding) | | | | | |
| **Study stage** | **Ethics (Patient safety & rights)**  **Patient Priorities (values & preferences)** | **Relevance for patients, society, and the research community** | **Minimization of bias (internal validity)** | **Precision (statistical validity)** | **Transparency / Access to data** | **Generalisability (external validity)** |
| **Planning & Preparation** | - Adherence to regulations/laws (local, national, international) and guidelines (e.g. GCP, GMP) - Thoughtful checking of feasibility (e.g. through pilot study or dryruns) - Approval by ethics committee & regulatory agency (as appropriate) - Appropriate consideration of equity (e.g. no selection towards racial minorities) - Appropriate participation options for special patient populations (e.g. rare diseases, pediatrics, geriatrics, etc.) - If applicable, presence of independently replicated, valid and robust preclinical data - Involvement of patient representatives/ advocates, their values and preferences, in the design and planning of the study | - Identification and discussion of uncertainties in existing evidence - Add-on value to already existing evidence taking into consideration burden of disease and anticipated benefit of treatment (i.e. expands or challenges current knowledge, opens additional areas for new research activity, or is a justifiable replication of existing evidence) - Therapeutic outcome measures/endpoints: clinical (judicious use of surrogate endpoints) , patient-relevant, well-defined, pre-specified, valid, reliable, sensitive to important change and measured at appropriate times to enable comprehensive assessment of benefits and harms) - Use of adequate methods (if applicable: innovative / original) - Assessment of economic impact of study - Clinically meaningful control group (e.g. clinically relevant intervention rather than “no treatment”) | - Prespecification of outcomes, analysis, subgroups, etc. - Manage conflicts of interest - Pre-study audit of all sites and investigators involved in study - Plan for minimization of bias according to research question (non-exhaustive):   RCTs :   - selection bias (e.g. randomization including allocation concealment) - Performance and detection bias (e.g. blinding and unblinding of patients, care-givers, and outcome assessors, endpoint judgements by endpoint committee) - Attrition bias (e.g. minimizing losses to follow-up)   Observational studies:   - careful planning of data collection (eg. considering all relevant confounders) - Healthcare access bias - Selection bias (e.g. non-random sampling bias, matching) - Attrition bias (e.g. ensuring complete follow-up)   Diagnostic studies:   - Spectrum bias - Partial work-up bias   Cohort studies:   - Misclassification bias - Detection bias | - Precise estimation of number of eligible patients, consent rate (eg. through pilot study) - Precise estimation of treatment effect and event rates in intervention and control groups (e.g. comprehensive consideration of previous evidence through systematic review and meta-analysis, non-inferiority and equivalence margins, etc.) Clearly justified sample size to measure expected impact - Definition of precise and reliable outcome measurements - Pre-specified minimization of missing data - Planning of recruitment procedures & monitoring | - Accessible trial protocol - Registration in publicly accessible database / registry (making objectives and methods transparent early on) - Protocol design & description in accordance with SPIRIT (e.g. detailed description of all interventions) - National and international peer-review of protocol (e.g. for funding/grants) - Aplan for dealing with “partial success” | - Avoidance of unnecessary restrictions in choice of inclusion/exclusion criteria (for rapid accrual, broader generalization, pragmatic study) - Subjects representative of patients who would use the drug / intervention - Definition of standard of care/current practice which matches real-world practice |
| **Conduct incl. Data Collection, Management, Analysis, Interpretation** | - Respect for and consideration of patient rights, well-being, dignity & safety throughout conduct of study: - Assurance of patient rights through freely given informed consent - Assurance of patient safety through adequate monitoring and reporting of side effects, AEs, SAEs etc. to RECs - Protection of subject privacy & confidentiality (during & after trial) - Assurance of patient participation and cooperation throughout conduct of study | - Collection of cost data (cost-effectiveness) - Conclusive inference about clinically meaningful treatment effect possible | - Accurate data collection and analysis as pre-specified - Clear identification of post-hoc analyses as exploratory - Adequate monitoring approach (e.g. risk based) - Data analysis using standard, generally accepted software - Minimization of confounding and selection bias (e.g. multivariable analysis, intention to treat principle) - Statistical adjustment for prognostic factors in the analysis (observational) | - Use of precise and reliable outcome measurements - Assurance of recruitment as pre-defined through enrollment monitoring (incl. adaptions if needed) - Systematic data recording & collection - Use of formal techniques to monitor/assess protocol compliance - Use of formal techniques to monitor/assess patient compliance | - Dissemination of protocol amendments to appropriate parties - Detailed methods disclosed to enable reproducibility - Conduct of internal audits and truthful reporting - External and independent DMC(e.g. for interim analyses) | - Patient follow-up close to clinical practice - Study protocol/procedures well adapted to routine clinical practice |
| **Report & Dissemination** | - Products/interventions made available to subjects after trial (access to treatment, if applicable) - Explicit reporting of approval from an IRB/EC - Adherence to all regulatory reporting timelines - Adequate reporting of side effects, AEs, SAEs, etc. to consumers and the scientific community - Declaration of conflict of interest (integrity) - Information of patients about trial outcome/treatment arm Involvement of patient representatives in the reporting of the study, i.e. for layterm summaries etc. | - Citation indeces/citations in clinical guideline - Critical reflection on research findings to guide the directions of future research - Reporting of challenges and mistakes to improve future research | - Reporting of all patient-relevant outcomes as pre-specified(no selective reporting) | - Reporting of absolute and relative treatment effects with confidence intervals - Reporting of actual number of recruited patients - Clear specification of analysis set (i.e. how many and which patients were considered in analysis) | - Maximisation of dissemination through open access - No selective reporting of study results - Avoidance of plagiarism & self-plagiarism - Independent and national/international peer review - Avoidance of spin in reporting of results - Record-keeping & archiving - Adherence to reporting guidelines (e.g. CONSORT, STROBE, PRISMA) to facilitate critical appraisal and reproducibility - Available anonymized individual participant data (data sharing) - Publication in journals requiring full protocol and statistical analysis plan - Maximisation of dissemination through use of alternative media other than medical journals - Publicly available doctoral/master theses | - Impact on guideline recommendations - Impact on clinical practice/future decisions - Clear reporting of inclusion and exclusion criteria, proportion of patients who declined randomization, and characteristics of included patients - Disclosure of detailed methods to enable reproducibility |
|  | **Sustainability** | | | | | |
|  | - Involvement of doctoral students/junior researchers/young clinicians under supervision of senior researchers in study design, planning, protocol - Hands-on experience of doctoral students/junior researcher/young clinicians in study conduct (e.g. data collection, management, analysis - Involvement in scientific writing & presentations (e.g. publications, conferences, symposia etc.) - Community & provider education and outreach; facilitation of two-way communication (lay language) with diverse populations and community groups - Knowledge transfer & exchange among clinical research groups - Improvement of awareness about value of clinical research to patients and society as a whole - Continuous adaptation and improvement to changes, developments, issues, and conditions during research continuum (quality by design) - Stable funding for research infrastructures (TBD) | | | | | |

**Delphi round 3**

| **Study Stage I: Concept**  **Milestone: Research question including study type defined and viable** | | |
| --- | --- | --- |
|  | | |
| **Dimension** | **Main question** | **Examples** |
| **Ethics**  **(Patient rights & safety)** | Can the research question be answered in the given setting? | Based on a rough estimate of required sample size, are enough potential study participants/ patients available in the given setting to answer the research question? |
|  |  | Based on a rough budget estimate, is it feasible to answer the research question with a specified study type? |
|  | Does study consider equity appropriately? | Are subjects selected so that : |
|  |  | stigmatized and vulnerable individuals are not targeted for risky research? |
|  |  | socially powerful individuals are not favored for potentially beneficial research? |
| **Relevance /**  **Patient centeredness** | Is significant add-on value to already existing evidence given, taking into consideration burden of disease and anticipated benefit of treatment? | Are uncertainties in existing evidence identified and discussed in a systematic review? |
|  |  | Does research: |
|  |  | Expand or challenge current knowledge? |
|  |  | Open additional areas for new research activity? |
|  |  | Justify replication of existing evidence, if applicable? |
|  | Are patient representatives/ advocates and their values and preferences involved in the development of the research question? |  |
|  | Are outcome measures patient-relevant, well-defined, pre-specified, valid, reliable and measured at appropriate times? | Are outcomes: |
|  |  | patient-relevant (judicious use of surrogate endpoints)? |
|  |  | well-defined (upfront)? |
|  |  | valid (measure what they intend to measure)? |
|  |  | reliable(stable and consistent when repeatedly measured) ? |
|  |  | sensitive to important change? |
|  |  | measured at appropriate times? |
| **Minimization of bias**  **(internal validity)** | Is the selected study type/design appropriate to minimize bias? | Is the study randomized or, if not sensible, appropriately controlled for confounding? |
| **Precision**  **(statistical validity)** | N/A |  |
| **Transparency / Access to data** | Is the research question clearly specified (including applicable PICO elements)? | Is each component of PICO clearly defined i.e. : |
|  |  | Patient population to be recruited in the study |
|  |  | Intervention to be assessed, |
|  |  | Control intervention as comparator, |
|  |  | Outcomes to be measured? |
| **Generalizability**  **(external validity)** | Are planned study participants representative of patients who would use the drug/intervention/diagnostic test in a real-life setting? | Are unnecessary restrictions through inclusion/exclusion criteria avoided (to facilitate rapid accrual, broader generalization, pragmatic study conduct)? |
|  |  | Is the control group adequate given current evidence and clinical practice (e.g. “standard of care” rather than “no treatment”)? |

| **Study Stage II: Planning & Feasibility**  **Milestone: Protocol developed and approved by regulatory bodies** | | |
| --- | --- | --- |
|  | | |
| **Dimension** | **Main question** | **Examples** |
| **Ethics**  **(Patient safety & rights)** | Does study adhere to applicable national and international regulations and laws? | Are study documents (e.g. protocol, patient information etc.) written in accordance with applicable national (and international, if applicable) regulations/laws? |
|  |  | Are informed consent documents written in lay language and easily understandable for study participants? |
|  |  | Has approval been obtained from ethics committee? |
|  |  | Has approval been obtained from regulatory agency (if applicable)? |
|  | Has feasibility been checked thoughtfully based on existing evidence? | Is valid and robust preclinical data present (if applicable)? |
|  |  | Has a pilot study been considered? |
|  |  | Are recruitment assumptions realistic (e.g. empirical data from electronic health records or from pilot study present)? |
|  |  | Have national/ international study registries been checked for studies that could interfere with the planned study? |
|  |  | Do anticipated study costs (preparation, conduct, analysis, dissemination) match with available budget? |
|  |  | Is study cost data related to planning, conduct, analysis, and dissemination planned to be collected (if applicable)? |
|  | Is collection, documentation, and reporting of Adverse Events / Serious Adverse Events according to the applicable regulations planned and specified in the protocol? |  |
| **Relevance /**  **Patient centeredness** | Is knowledge transfer/use (e.g. plans for inclusion of results in clinical guidelines) planned? | Are relevant guideline groups identified and contact established? |
|  |  | Are patient representatives involved in protocol development? |
| **Minimization of bias (internal validity)** | Is statistical analysis pre-specified? | Are outcomes, datasets, subgroups, handling of missing data, etc., pre-specified? |
|  | Is trial monitoring considered and documented in a monitoring plan? |  |
|  | Is data management planned and documented in a data management plan? |  |
|  | Is minimization of bias planned for according to the research question and study design? | Exemplary items according to study type: |
|  | **Randomized Controlled Trials** | *Please also refer to Cochrane Risk of Bias tool for RCTs ^1^ for full list of items.* |
|  |  | Is randomization adequate and concealed? |
|  |  | Are (known) prognostic factors distributed equally (i.e. are groups prognostically balanced at the start of the trial)? |
|  |  | Is blinding of patients and/or care-givers adequate? |
|  |  | Are concomitant interventions documented? |
|  |  | Is blinding of outcome assessors adequate? |
|  |  | Are plans to minimize losses to follow up present? |
|  |  | Are plans to analyze study participants in groups as randomized present? |
|  | **Observational studies (incl. cohort studies)** | *Please also refer to ROBINS-I tool ^2^ for full list of items.* |
|  |  | Is collection of data carefully planned, i.e. are all relevant confounders considered and measured? |
|  |  | Are all study participants selected or recruited from the same or similar populations (incl. the same time period)? |
|  |  | Do the study participants represent the cases originated in the community? (e.g. due to issues with healthcare access) |
|  |  | Are inclusion and exclusion criteria pre-specified and applied uniformly to all study participants? |
|  |  | Are plans to minimize losses to follow-up present? |
|  |  | Is timeframe sufficient so that one can reasonably expect to see an association between exposure and outcome if it existed? |
|  |  | For exposures that can vary in amount or level, does the study examine different levels of the exposure as related to the outcome (e.g. categories, or exposure measured as continuous variable)? |
|  |  | Is exposure measured more than once over time? |
|  | **Diagnostic accuracy studies** | *Please also refer to QUADAS-2 Risk of Bias tool ^3^ for full list of items.* |
|  |  | Is there an independent, blind comparison between index test and an appropriate gold standard of diagnosis? |
|  |  | Is the diagnostic test evaluated in a representative, and ideally full spectrum of study participants/ patients (like those in whom it would be used in practice, spectrum ranging from mild to severe, and early to late cases of target disorder)? |
|  |  | Is a reference standard applied regardless of the index test results (ideally both index test and reference standard should be carried out on all study participants/ patients)? |
|  |  | If no, is it planned to follow up study participants/ patients for an appropriate period of time (dependent on disease in question) to see if they are truly negative? |
| **Precision**  **(statistical validity)** | Are expected treatment effects and event rates in intervention and control groups realistic and estimated based on empirical evidence? | Is number of eligible study participants/ patients precisely estimated? |
|  |  | Is consent rate precisely estimated? |
|  |  | Are treatment effects and/or event rates estimated in both intervention and control groups? |
|  |  | If yes, are they based on evidence such as systematic literature reviews, meta-analysis? |
|  |  | Is rationale for non-inferiority / equivalence design provided (if applicable)? |
|  |  | Is rationale for maximum clinically acceptable difference (equivalence margins) provided (if applicable)? |
|  | Is sample size clearly justified to measure expected impact? | Is sample size realistically estimated and clearly described (incl. assumed treatment effects, references for estimates, power, alpha error, and expected losses to follow-up)? |
|  |  | Is rationale for sample size given if not derived statistically? |
|  | Are recruitment procedures and recruitment monitoring planned? |  |
| **Transparency / Access to data** | Is the protocol in accordance with SPIRIT-guideline? | *Please also refer to the SPIRIT tool ^4^ for full list of items.* |
|  |  | Is protocol peer-reviewed? |
|  |  | Is full trial protocol accessible and published? |
|  |  | Is study registered in publicly accessible database / registry? |
|  |  | Does protocol state a plan on how to deal with study publication in case target sample size could not be achieved/study had to be discontinued prematurely? |
| **Generalizability**  **(external validity)** | Are study procedures well adapted to routine clinical practice? | Is standard of care/current practice clearly defined? |
|  |  | Are realistic interventions applied which are carried out by physicians in everyday practice? |
|  |  | Is patient-follow up close to clinical practice? |

| **Study Stage III: Conduct**  **Milestone: Last patient last visit** | | |
| --- | --- | --- |
|  | | |
| **Dimension** | **Main question** | **Examples** |
| **Ethics**  **(Patient safety & rights)** | Is respect for and consideration of patient rights, well-being, dignity & safety throughout conduct of study guaranteed? | Are study participants respected at all times, i.e.: |
|  |  | Is withdrawal from study at any time explicitly permitted? |
|  |  | Are study participants informed of newly discovered risks? |
|  |  | Are study participants informed about purpose of research, its procedures and potential risks, benefits and alternatives, so that they can make a voluntary decision? |
|  |  | Are side effects / AEs/ SAEs, SUSARs etc. monitored and reported to the ethics committee within required timeframes? |
|  |  | Is study participants’ privacy and confidentiality ensured during (and after) trial, e.g. through appropriate coding? |
| **Relevance /**  **Patient centeredness** | Are there any measures in place to assure study participants’ participation and cooperation throughout conduct of study (e.g. incentives, phone calls, etc.)? |  |
| **Minimization of bias (internal validity)** | Is data systematically collected as pre-specified in protocol? | Is data collected as pre-specified in the protocol? |
|  |  | Are losses to follow-up minimized? |
|  |  | Are protocol deviations documented, and reported to the respective institutions? |
|  |  | Are changes in study procedures amended in the protocol? |
|  | Is attrition bias minimized? | Do the reasons for dropping out have an impact on the assessment of compliance, effectiveness or safety? |
|  |  | Are missing data documented by individual outcomes? |
|  | Is performance bias minimized? | Apart from the allocated treatment, are study groups treated equally (e.g. no additional treatments or tests)? |
|  |  | If applicable, are study participants and clinicians kept "blind" to which treatment was being received? |
|  | Is monitoring conducted according to the pre-specified monitoring plan? |  |
| **Precision**  **(statistical validity)** | Is enrollment of study participants monitored? | Are formal techniques in place to monitor recruitment centrally and at participating sites? |
|  |  | Are measures in place to allow timely reaction in case recruitment deviates from expectations? |
|  | Are any formal techniques to monitor/assess protocol compliance of participants and study staff in place? |  |
| **Transparency / Access to data** | Is trial conduct transparent to all involved parties? | Are protocol amendments disseminated to appropriate parties within reporting timelines? |
|  |  | Are internal or external audits planned, conducted and reported? |
|  |  | Is an external and independent Data Monitoring Committee present, or reason provided, why it is not needed? |
| **Generalizability (external validity)** | Are numbers of participants through different stages of a study documented (patient flow) including reasons for leaving the study before its end? | Is proportion of study participants who declined randomization documented? |
|  |  |  |
|  |  | Are the reasons for participants leaving the study before its scheduled end documented? |

| **Study Stage IV: Analysis & Interpretation**  **Milestone: Study data analyzed and interpreted** | | |
| --- | --- | --- |
|  | | |
| **Dimension** | **Main question** | **Examples** |
| **Ethics**  **(Patient rights & Safety)** | N/A |  |
| **Relevance /**  **Patient centeredness** | Is an inference about clinically meaningful treatment effects possible? |  |
| **Minimization of bias**  **(internal validity)** | Is the data analyzed as pre-specified in the protocol? | Is data analyzed as pre-specified in protocol? |
|  |  | Are post-hoc analyses clearly labelled as such or as exploratory analyses? |
|  |  | Is data analysis performed using standard, generally accepted software? |
|  |  | Are data assumptions checked (e.g. normal distribution) as appropriate for planned statistical tests/modelling? |
|  | Are key confounding variables adjusted for in the analysis (e.g. multivariable analysis)? |  |
|  | Is the intention-to-treat principle followed (i.e. study participants were analyzed in groups as randomized) in case of a superiority hypothesis? |  |
|  | Are both a per-protocol and an analysis following the intention-to-treat principle conducted in case of a non-inferiority hypothesis? |  |
|  | Are results interpreted without “spin”? |  |
| **Precision**  **(statistical validity)** | Is the uncertainty of results through missing outcome data considered in the analysis e.g. through reasonable sensitivity analyses? |  |
| **Transparency / Access to data** | Is the analysis code clearly documented and the analysis process reproducible? |  |
| **Generalizability**  **(external validity)** | N/A |  |

| **Study Stage V: Reporting & Dissemination**  **Milestone: Study archived and published** | | |
| --- | --- | --- |
|  | | |
| **Dimension** | **Main question** | **Examples** |
| **Ethics**  **(Patient safety & rights)** | Was study completion/termination communicated to appropriate parties and documented in registries? | Was study completion/termination reported to ethics committee/regulatory bodies? |
|  |  | Was study completion/termination appropriately documented in national/international registry? |
| **Relevance /**  **Patient centeredness** | Did authors critically reflect on research findings (results as well as challenges or mistakes during study conduct) and the implications for future research? |  |
|  | Is the study easily available to decision/policy/guideline makers? | Has the study been cited in a clinical guideline? |
|  | Were study participants involved in the reporting of the study? | Were study participants informed about outcome of the study? |
|  |  | Had patient representatives been involved in reporting of the study, e.g. in writing of lay term summaries? |
|  | Did study participants get access to products/interventions after trial? |  |
| **Minimization of bias**  **(internal validity)** | Were all outcomes and important trial characteristics reported as pre-specified in the protocol (outcome reporting bias prevented)? | Were all patient-relevant outcomes reported as pre-specified in the protocol? |
|  |  | Were important modifications to the protocol (e.g. premature discontinuation) reported (if applicable)? |
| **Precision**  **(statistical validity)** | Were absolute and relative treatment effects reported accompanied by confidence intervals? |  |
|  | Was the analysis set of participants clearly specified? | Were the actual numbers of recruited, randomized (if applicable), followed-up, and analyzed participants reported for each outcome and for each treatment group (if applicable)? |
| **Transparency / Access to data** | Was dissemination of data and study results maximized? | Was dissemination maximized through open access? |
|  |  | Was anonymized individual participant-level data made available (data sharing)? |
|  |  | Were study results posted in trial registries? |
|  |  | Did publication in journals include full protocol and statistical analysis plan? |
|  |  | Was dissemination maximized through use of alternative media other than medical journals? |
|  |  | Were resulting doctoral/master theses made publicly available (if applicable)? |
|  | Were reporting guidelines followed to facilitate critical appraisal and reproducibility? | *Was reference made to reporting guidelines such as CONSORT (Randomised trials) ^5^, STROBE (Observational studies) ^6^, STARD (Diagnostic studies) ^7^, or PRISMA (Systematic reviews) ^8^ depending on the respective study design.* |
|  |  | Were detailed methods disclosed in publications (to enable reproducibility)? |
|  | Were selective reporting, spin, plagiarism and self-plagiarism avoided and conflicts of interest declared? | Was selective reporting of study results avoided? |
|  |  | Was plagiarism and self-plagiarism avoided? |
|  |  | Were the study results independently peer reviewed? |
|  |  | Was spin avoided in reporting of results? |
|  |  | Were conflicts of interest declared? |
|  | Was knowledge transfer & exchange fostered? | Was knowledge transfer & exchange fostered through e.g.: |
|  |  | Community and provider education and outreach |
|  |  | Facilitation of two-way communication (lay language) with diverse populations and community groups |
|  |  | Knowledge transfer & exchange among clinical research groups |
|  | Were records kept and archived? |  |
| **Generalizability**  **(external validity)** | Did results impact clinical practice? | Did results impact guideline recommendations? |
|  | Were characteristics of included patients clearly reported? | Were inclusion and exclusion criteria clearly reported? |
|  |  | We characteristics of included patients clearly reported? |

| **Sustainability / Education** | |
| --- | --- |
|  | |
| **Main question** | **Examples** |
| Are doctoral students, junior researchers, or young clinicians actively involved in all stages of a clinical study, and reliably supervised/mentored by senior researchers? | Are doctoral students, junior researchers, or young clinicians actively involved in study design, planning, conduct, analysis, interpretation and dissemination of results (e.g. publications, conference presentations)? |
|  | Are doctoral students, junior researchers, or young clinicians actively supervised by senior researchers at all stages of a clinical study? |
|  | Are doctoral students, junior researchers, or young clinicians mentored as to career options in clinical research? |
|  | Are training options and courses in health research methodology available for principal investigators and staff? |
|  | Are doctoral students, junior researchers, or young clinicians mentored to improve awareness about value of clinical research to patients and society as a whole? |
|  | Are processes continuously adapted and improved to changes, developments, issues, and conditions during research continuum (quality by design)? |

| **Infrastructure** |  |
| --- | --- |
|  | |
| **Main question** | **Examples** |
| Is a Quality Management System incl. Standard Operating Procedures (SOPs) in place? | Is all staff continuously trained in applicable SOPs?  Are there measures in place to control, whether the existing Quality Management System is followed? (i.e. internal audits) |
| Is a critical mass of well-trained and experienced principal investigators and study staff present? | Has the principal investigator and/or staff been involved in clinical studies before? |
|  | Is all staff continuously trained in GCP and protocol-related activities? |
|  | Is training (e.g. GCP) of each participating investigator and staff member clearly documented? |
|  | Are roles and responsibilities of each participating investigator and staff member clearly documented? |
|  | Are all involved stakeholders well and adequately informed about study procedures and changes? |
| Are expert epidemiologists/methodologists, statisticians, professional data managers, and/or a logistical support unit involved early-on? | Are epidemiologists/methodological specialists involved in development of protocol? |
|  | Are statisticians involved in development of protocol? |
|  | Are data managers involved in the development of the data management plan and the setup of the data management system? |
|  | Is a logistical support unit involved in study planning and/or conduct, e.g. through regulatory affairs experts, study nurses, or project managers? |
| Are adequate human, material, and equipment resources available for study conduct? | Is dispense, transport, and storage of investigational medicinal product, if applicable, planned? |
|  | Is availability of study-specific materials, hardware, and facilities planned and secured? |
|  | Is a transparent study budget available and approved by experienced personnel, including costs for experts mentioned above? |
|  | Is funding secured through acquisition of competitive money or through collaboration with e.g. industry partners? |
| Are adequate facilities ensuring data security and privacy in place (incl. competent and effective IT support to facilitate solutions tailored to specific challenges of individual studies)? | Is an electronic database incl. audit trail in place? |
|  | Is patient data coded? |
|  | Is IT support present at site? |
| Is inter-/multidisciplinary collaboration and involvement in clinical trial planning and conduct fostered? | Have all relevant stakeholders been involved in protocol development and conduct? (e.g. investigators at other trial sites, etc.) |
|  | Is communication between involved staff, sponsor, contractors, and site fostered? |
| Is it ensured that all studies which are subject to compulsory insurance have insurance at all applicable institutions? |  |

**Delphi round 4**

| **Study Stage I: Concept**  **Milestone: Research question including study type defined and viable** | | |  |
| --- | --- | --- | --- |
|  | | |  |
| **Dimension** | **Specific question** | **Examples** | **Reference for changes** |
| **Protection of patient safety & rights** | Can the research question be addressed in the given setting? | Based on a rough resource assessment, and potentially available study participants, is it feasible to answer the research question? | Comment 7,35 |
|  |  | Based on a rough budget estimate, is it feasible to answer the research question with a specified study type? |  |
|  | Does the study consider equity appropriately? | Are participants selected so that : | Comment 28 |
|  |  | - vulnerable individuals are neither targeted for risky research nor withheld from research relevant to these populations? | Comment 49, 54 |
|  |  | - socially powerful individuals are not favored for potentially beneficial research? |  |
|  | Is the research design adequate for the stage of an investigated technology to ensure patient safety? | Are sufficient data on toxicity/teratogenicity of an intervention available from animal studies or phase I studies? | Comment 43 |
|  | Do the (assumed) short and long term benefits of the study outweigh potential risks associated with the study (consistent with clinical equipoise)? |  | Comments 14,36 |
| **Relevance /**  **Patient centeredness & involvement** | Is significant potential add-on value to existing evidence (systematic review)specified, taking into consideration burden of disease and anticipated benefit of treatment? | Are uncertainties in existing evidence identified and discussed in a systematic review? | Comment 1 |
|  |  | Does research: |  |
|  |  | Expand or challenge current knowledge? |  |
|  |  | Open additional areas for new research activity? |  |
|  |  | Justify replication of existing evidence, if applicable? |  |
|  | Aare patient representatives/ advocates and their needs and values adequately involved in the development of the research question? |  | Comment 6, 49 |
|  | Are outcome measures patient-relevant? |  |  |
|  |  | Are outcomes patient-relevant according to COMET (including quality of life, if applicable, and with judicious use of surrogate endpoints)? | Comment 15, 7 |
|  |  |  |  |
|  |  |  |  |
|  |  |  |  |
|  |  |  |  |
|  |  |  |  |
|  |  |  |  |
| **Minimization of bias**  **(internal validity)** | Is the selected study type/design appropriate to minimize bias? | Is the study randomized or, if not, appropriately controlled for confounding? |  |
|  | Are outcome measures well-defined, pre-specified, valid, reliable and measured at appropriate times? | Are outcomes:  well-defined (upfront)? | Comment 15, 7 |
|  |  | valid (measure what they intend to measure)? |  |
|  |  | reliable(stable and consistent when repeatedly measured)? |  |
|  |  | sensitive to important change? |  |
|  |  | measured at appropriate times? |  |
|  |  | standardized across studies (core outcome sets, if applicable) |  |
| **Precision**  **va** | Has an estimate of the required sample size been made (for feasibility purposes, see “Protection of patient safety & rights”)? |  | Comment 7, 35 |
| **Transparency / Access to data** | Is the research question clearly specified (e.g. in a synopsis) ~~(including applicable PICO elements)~~? | Is each component of P(I/E)(C)O^1^ as applicable to study design clearly defined in a synopsis i.e. : | Comments 13, 16, 18, 20, 28, 30 |
|  |  | - Patient population to be recruited/investigated in the study |  |
|  |  | - Intervention to be assessed, |  |
|  |  | - Exposure to be assessed, |  |
|  |  | - Diagnostic Test to be assessed, |  |
|  |  | - Control intervention as comparator, |  |
|  |  | - Outcomes to be measured? |  |
| **Generalizability**  **(external validity)** | Are planned study participants representative of patients who would use the drug/intervention/diagnostic test in a real-life setting? | Are unnecessary restrictions through inclusion/exclusion criteria avoided (to facilitate rapid accrual, broader generalization, pragmatic study conduct)? |  |
|  |  | Is the control group adequate given current evidence and clinical practice (e.g. “standard of care” rather than “no treatment”)? |  |

^1^P: Population, I: Intervention, E: Exposure, C: Comparator, O: Outcome

**Detailed comments from survey participants, including answers by the authors**

| **Participant ID** | **Participant agrees on main question(s)** | **Comment by survey participant** | **Answer by the authors** |
| --- | --- | --- | --- |
| 1 | No | Well, I sort of agree, however, I suggest to revise / amend the main category and beside patient-centeredness also include patient engagement (as the questions imply this) | We agree that the aspect of patient engagement and involvement is not yet covered sufficiently in the dimension name. We have therefore renamed the dimension to “Relevance / Patient centeredness & involvement”. |
| 2 | Yes | Overall, I agree. I wonder though whether under "relevance" comparators should be mentioned. Studies are most relevant if they compare interventions that are used in real life in a fair manner. E.g. comparable dosing | Please refer to our last item in “examples” in the “Generalizability” dimension. |
| 5 | Yes | I think statistical validity may be structured in more detail. | We refrained from structuring questions around validity in more detail as the concept stage should include rough assessments and estimates which lead to a primary research question and a study synopsis (i.e. a one-page summary). Items required for the actual study protocol are covered in more detail at the “Planning stage”. |
| 6 | Yes | To relevance/patient centeredness: The development of a research question must be based on scientific grounds and the research plan discussed with the patient. "Values and preferences of the patients" can influence the study procedure, but only rarely the development of a research question. Scientific questions must not be influenced by emotional preferences etc. | We have slightly modified the main question “Are patient needs and values considered in the development of the research question?”. All clinical research should ultimately serve patients, i.e. the scientific question should be posed with the “usefulness/benefit for patients” in mind. Therefore, to pose questions which are relevant to patients and the society as a whole, patient needs and values should be identified and considered whenever possible. |
| 13 | No | Minimization of bias: question pertains only to selection of study type / design but not to other methods. Ok if this will be covered in later stages but it's slighlty confusing that last question (planned study participants) is already quite specific re methods. | Planned study participants are part of PICO and therefore a basic component of the conceptualization of a study. Detailed questions related to minimization of bias will be covered according to the specific study design in the planning stage. |
| 14 | Yes | While I agree with the questions, perhaps there can be a little more emphasis on the question of patient burden with regard to the trial versus benefit? i.e. does the patient fully appreciate the short and long term benefits of the trial versus the procedures etc they are asked to go through in the trial. | We agree that this aspect is not covered yet in our ethics dimension. We added a question on risk/burden-benefit to the “concept” as well as to the “planning” stage of our framework. |
| 15 | Yes | Outcome measures: quality of patient live centered, internationally used (ready to compare) p.e. ICCHOM criterias | We agree and have adapted the question on outcome measures as follows:  Are outcomes:   - patient-relevant (including quality of life if applicable, and with judicious use of surrogate endpoints) (Relevance, patient centeredness & involvement) and - standardized across studies (core outcome sets, if applicable) (Minimization of bias) |
| 16 | No | I am not sure that PICO relates to transparency/Access to data. | We clarify that we would expect the main research question, including the PI/ECO components, to be specified and documented during the concept stage, for example in a study synopsis based on which the study protocol/plan may be developed. This documentation therefore applies to the transparency/access to data dimension which includes reporting of crucial aspects at all stages of the study. |
| 18 | No | This is mostly appropriate for intervention studies. In that sense, I believe it is a useful grid. But what about diagnostic studies? epidemiological studies? other types of clinical research. This is a framework for only a fraction of clinical research. | We agree that specifically the question on PICO was too narrowly formulated. We therefore adapted this main question such that other study designs (epidemiological studies, diagnostic studies, etc.) are also covered. All other quality dimensions should apply to all study types. |
| 20 | No | Main problem with transparency /access to data. The grey question explores a different dimension, mostly related to generalizability. | We clarify that we would expect the main research question, including the PI/ECO components, to be specified and documented during the concept stage, for example in a study synopsis based on which the study protocol/plan may be developed. This documentation therefore applies to the transparency/access to data dimension which includes reporting of crucial aspects at all stages of the study. |
| 25 | Yes | Comment: in the Relevance/Outcomes part; examples are too repetitive of the main (grey) quality questions... | The examples in this dimension serve to clarify the content of the main quality questions and therefore contain more detail (in brackets) than the main questions. |
| 26 | Yes | But I would emphasize questions about feasibility. A large part of discontinued studies (and waist) are due to lack of recruitment, too small budget and other factors which might be anticipated with feasibility survey. | We agree that feasibility assessments are a crucial component of study planning. Therefore, we include a question on rough feasibility assessment in the conceptualization phase (first main question) and more detailed questions in the planning stage. |
| 28 | Yes | would strongly consider to replace "subjects" with "participants" "PICO" elements? Please refrain from using acronyms | We have replaced “subjects” with “participants” throughout the framework. All components of “PICO” are now written out in the examples column. |
| 30 | No | I expect different questions for the section on transparency/access to data, the given question about PICO belong to the Design aspects (in minimization of bias) In Generalizability the example about the standard of care vs. no treatment should be part of the PICO (Control) discussion. I think here the things are still mixed and not ordered consistently! | Please refer to our answer to comment 8 and 10 with respect to your first remark.  While we agree that the comparator is mentioned in two different dimensions, we think that there are different levels. For “Minimization of bias”, we ask for a description of the comparator, while in “Generalizability” we ask for a comparator that is relevant and adequate given current evidence, i.e. does the chosen comparator allow for generalizability of the research results. |
| 31 | No | Very difficult to comment within the framework of this online survey, e.g. I cannot copy-paste the questions above ... The first question as I understand from the examples is not related to Ethics but rather Precision. The first in Relevance could also be moved to Ethics. Some parts of the third question in Relevance relate to Internal Validity (and Generalizability) The question in Transparency is the first question for internal validity Transparency may not be an issue at this stage although I could imagine: "Are there any barriers to transparency/access to data" envisaged at this stage?" | A first rough feasibility assessment at the concept stage is strongly linked to an ethical question on whether the study should be conducted at all or not. Initiating a study which is foreseeably not feasible should be considered a waste of resources and compromises participants’ safety and rights. However, we added a question specifically about a rough estimate of sample size to the “Precision” dimension and linked this question to the “Ethics” dimension. For your other comments, please refer to our answers to the comments above.  We further agree that some parts of the third question in “Relevance” on outcome measures relate to internal validity. We therefore split the question in two parts: one related to patient-centeredness, and one related to internal validity. |
| 33 | No | The design of the questionnaire is not adequate. Yes/No options do not permit a balanced response. At least, the category "partially agree" should be introduced. I partially agree on the main questions: 1. the questions are too complicated/detailed 2. the questions are not weighted, i.e. the most important criterion "Novelty/potential gain of knowledge" needs to be no. 1. | We added a general description of the framework in order to explain the content and meaning of each individual dimension in a separate file on “Framework Structure”. Within our “Relevance” dimension, we include novelty and innovation of research, but we also consider replication of studies valuable in case the existing evidence is not sufficient/conclusive. Our first question in the “Relevance” dimension expands on add-on value, replication, or novel areas of research. We explicitly refrain from weighting criteria as this may depend on the study design or the stakeholder applying the framework. All dimensions over all research stages are considered to be important. |
| 35 | Yes | Precision (statistical validity) is already important here (power calculation) Transparency/Access to data needs a data management plan | We added a main question on a rough estimate of the sample size in “Precision” which allows conducting first feasibility assessments. The data management plan will be covered in the planning stage of the study. |
| 36 | No | With respect to ethics add at least positive benefit risk relationship (that comprises research value / relevance on the one Hand and risks / burdens on the other hand). | We added a statement on positive risk/burden-benefit relationship in the concept and the planning stage of a study. |
| 43 | No | The availability of enough research participants and enough research funds should not be considered an ethical aspect. The number of potentially eligible study participants is related more closely to the statistical precision (of future study results). During the concept stage of research, quality would consist of anticipating possible ethical problems (e.g. questionable equipoise, high risk for study participants, etc.). The question whether outcome measures are well-defined is relevant for internal validity rather than for patient-centeredness. Replication of existing evidence is a matter of research relevance, but this aspect also affects the ethical dimension of research, because it may be unethical to replicate research findings that do not requires further replication. The aspect of 'safety' in the ethical dimension is not translated into a main quality question. When developing a study concept, it is an aspect of high-quality research that the research design is adequate for the stage of the technology. For example, a clinical trial will be unethical without animal studies done beforehand. During the stage of developing a research concept, it appears unnecessary or impossible to assess transparency and data Access, since only a one-page summary of a research idea will exist at this time-point. This dimension should be labelled "N/A". | A first feasibility assessment during the concept stage is strongly linked to an ethical question on whether the study should be conducted at all or not. Initiating a study which is foreseeably not feasible should be considered a waste of resources and compromises participants’ safety and rights. However, we added a question specifically about a rough estimate of sample size to the “Precision” dimension and linked this question to the “Ethics” dimension.  We have further added a statement on positive risk/burden-benefit relationship in the concept and the planning stage and added a main question on safety to the “Ethics” dimension.  In addition, we split the outcome question in two separate parts, one for patient-centeredness, and one for internal validity.  We agree that only a one-page summary may exist at the concept stage. We however believe that the research question, i.e. the PI/ECO, should be explicitly and clearly stated in the one-page summary. |
| 45 | No | You have 2 questions on patient selection - equity and generalizability which need to be reconciled (one question?) | We are aware of the fact that subject selection is presented in two different dimensions. We however think that there are multiple (and different) aspects on how patients are selected, i.e. whether they represent a broader patient population (generalizability) or whether they are selected in a fair, equitable way (ethics), and we would like to keep both of these aspects as main questions in order to consider both facets and indicate the importance of this process. |
| 49 | No | Two caveats: Study equity and research with vulnerable populations ... : This issue can be looked at in two ways: a) as done above: Studies are selected so that vulnerable individuals are not the target of risky research. b)vulnerable individuals are excluded from risky research. Up to 20 years ago, research in children was hardly done because they were vulnerable. ...; with the consequence, that clinical care lacked knowledge on how children differed in comparison to adults. Only a change in perspective ("society is withholding research on children") allowed to move forward with research in the particularities of this vulnerable population. @pt. relevance/pt.centredness: Is it mandatory to answer these questions with a yes? It can well be that in earlier phases of clinical research, where e.g. processes are investigated, it may not be necessary / not make sense to involve patients and their advocates, while I agree that this is important in outcome studies So, I am not sure whether I would agree to these items for all kinds of clinical research, and rather say, "it depends" | We agree that it may be misunderstood in the way you describe it under b). We therefore rephrased the item on equity. Regarding the “Relevance” dimension we agree that some study types require less direct patient involvement (e.g. Phase I) than outcome studies. However, as our framework explicitly focuses on research involving patients (and not healthy volunteers) all studies should at least consider patient engagement/involvement (please see also our reply to comment 4). |
| 54 | Yes | Question "Does study consider equity properly" I would suggest "Is the study population justified". The inclusion of vulnerable individuals (I wouldn't use "stigmatized individuals") in the study population should always be justified, not just for "risky research". Never heard of PICO?? Patient Informed Consent Or something? I would just use consent. | We have removed “stigmatized” from the question and rephrased as follows: “… vulnerable individuals are neither targeted for risky research nor withheld from research relevant to these populations” in order to clarify that they should not be predominantly selected for risky research but also not withheld from any research relevant to them (e.g. pediatrics).  We now explain PICO in a foot note. |

| **Study Stage II: Planning & Feasibility**  **Milestone: Protocol developed and approved by regulatory bodies** | | |  |
| --- | --- | --- | --- |
|  | | |  |
| **Dimension** | **Specific question** | **Examples** | **Reference to comments** |
| **Protection of patient safety & rights** | Do the (assumed) short and long term benefits of the study outweigh study burden (due to study visits, intervention, procedures etc.)? |  | Comment 24, 47 and Comment 14 and 36 from the “conceptualization stage” |
|  | Are patients’ safety and rights protected through the study’s adherence to applicable national and international regulations and laws? | Are study documents (e.g. protocol, participant information etc.) written in accordance with applicable national (and international, if applicable) regulations/laws? | Comment 31 in “overall framework structure”, Comment 31 |
|  |  | Are informed consent documents written in lay language and easily understandable for study participants? |  |
|  |  | Has approval been obtained from ethics committee? |  |
|  |  | Has approval been obtained from regulatory agency (if applicable)? |  |
|  | Has feasibility been checked thoughtfully based on existing evidence (systematic review)? | Is valid and robust preclinical data present (if applicable)? |  |
|  |  | Have crucial feasibility aspects (e.g. recruitment) been piloted? | Comment 30 |
|  |  | Are recruitment assumptions realistic in a specified timeframe (e.g. empirical data from electronic health records or from pilot study present)? | Comment 44 |
|  |  | Have national/ international study registries been checked for studies that could interfere with the planned study? |  |
|  |  | Do anticipated study costs (preparation, conduct, analysis, dissemination) match with available budget? |  |
|  |  | Is study cost data related to planning, conduct, analysis, and dissemination planned to be collected (if applicable)? |  |
|  | Is collection, documentation, and reporting of Adverse Events / Serious Adverse Events / Suspected Unexpected Serious Adverse Reaction according to the applicable regulations planned and specified in the protocol? |  | Comment 32 |
|  | Are mechanisms established (for example, through Data Monitoring Committees) which allow early study termination when required and prevent early study termination for inadequate reasons? | Is one or few interim analyses for safety considered? | Comment 43 from “conduct” stage |
|  |  | Is early stopping for benefit with insufficient collection of safety data avoided ^9^? | Comment 43 from “conduct” stage |
| **Relevance /**  **Patient centeredness & involvement** | Has knowledge transfer/use been considered (e.g. plans to take account of results in clinical guidelines) planned? | Are relevant guideline groups identified and contact established? |  |
|  |  | Are patient representatives involved in protocol development? |  |
| **Minimization of bias (internal validity)** | Is statistical analysis pre-specified (using outcomes as defined in concept stage)? | Are outcomes, datasets, subgroups, handling of missing data, etc., pre-specified? |  |
|  | Is study monitoring (adapted to risk of study, if applicable) planned and documented in a monitoring plan?? |  | Comment 19 |
|  | Is data management planned and documented in a data management plan, including by a Data Monitoring Committee? |  |  |
|  | Is minimization of bias been planned for in the study design, taking account of the research question? | Exemplary items according to study type (non-exhaustive): | Personal communication by expert 31 |
|  | **Randomized Controlled Trials** | *Please also refer to Cochrane Risk of Bias tool for RCTs ^1^ for full list of items.* |  |
|  |  | Is randomization adequate and concealed? |  |
|  |  | Are (known) prognostic factors distributed equally (i.e. are groups prognostically balanced at the start of the trial)? |  |
|  |  | Is blinding of participants and/or care-givers adequate? |  |
|  |  | Are concomitant interventions documented? |  |
|  |  | Is blinding of outcome assessors adequate? |  |
|  |  | Are plans to minimize losses to follow up present? |  |
|  |  | Are plans to analyze study participants in groups as randomized present? |  |
|  | **Observational studies (incl. cohort studies)** | *Please also refer to ROBINS-I tool ^2^ for full list of items.* |  |
|  |  | Is collection of data carefully planned, i.e. are all relevant confounders considered and measured? |  |
|  |  | Are all study participants selected or recruited from the same or similar populations (incl. the same time period)? |  |
|  |  | Do the study participants represent the cases originated in the community? (e.g. due to issues with healthcare access) |  |
|  |  | Are inclusion and exclusion criteria pre-specified and applied uniformly to all study participants? |  |
|  |  | Are plans to minimize losses to follow-up present? |  |
|  |  | Is timeframe sufficient so that one can reasonably expect to see an association between exposure and outcome if it existed? |  |
|  |  | For exposures that can vary in amount or level, does the study examine different levels of the exposure as related to the outcome (e.g. categories, or exposure measured as continuous variable)? |  |
|  |  | Is exposure measured more than once over time? |  |
|  | **Diagnostic accuracy studies** | *Please also refer to QUADAS-2 Risk of Bias tool ^3^ for full list of items.* |  |
|  |  | Is there an independent, blind comparison between index test and an appropriate gold standard of diagnosis? |  |
|  |  | Is the diagnostic test evaluated in a representative, and ideally full spectrum of study participants (like those in whom it would be used in practice, spectrum ranging from mild to severe, and early to late cases of target disorder)? |  |
|  |  | Is a reference standard applied regardless of the index test results (ideally both index test and reference standard should be carried out on all study participants)? |  |
|  |  | If no, is it planned to follow up study participants for an appropriate period of time (dependent on disease in question) to see if they are truly negative? |  |
| **Precision** | Are expected treatment effects and event rates in intervention and control groups realistic and estimated based on empirical evidence? | Is sample size realistically estimated and clearly described (incl. assumed treatment effects, references for estimates, power, alpha error, and expected losses to follow-up)? | Comment 15, 43, 49 |
|  |  | Is consent rate precisely estimated? |  |
|  |  | Are treatment effects and/or event rates estimated in both intervention and control groups? |  |
|  |  | If yes, are they based on evidence such as systematic literature reviews, meta-analysis? |  |
|  |  | Is rationale for non-inferiority / equivalence design provided (if applicable)? |  |
|  |  | Is rationale for maximum clinically acceptable difference (equivalence margins) provided (if applicable)? | Comment 15, 43, 49 |
|  |  | Is rationale for sample size given if not derived statistically? |  |
|  | Are recruitment procedures and recruitment monitoring planned to ensure sufficient sample size? |  |  |
| **Transparency / Access to data** | Does the protocol accord with established standards (e.g. SPIRIT or other applicable guidelines depending on study design)? | Is protocol peer-reviewed? | Comments 28, 35 |
|  |  | Is publication and accessibility of full study protocol planned? | Comment 49 |
|  |  | Is study registered in publicly accessible database / registry? |  |
|  |  | Does protocol state a plan on how to deal with study publication in case target sample size could not be achieved/study had to be discontinued prematurely? |  |
|  | Is there a dissemination plan for sharing study information, including the protocol, summary results, and participant level data? |  | Comment 13, Comment 24 |
| **Generalizability**  **(external validity)** | Are study procedures/observations in line with routine practice in the given setting? | Is standard of care/current practice clearly defined? | Comment 9, 20, 28 |
|  |  | Are interventions /observations close to foreseen everyday practice? | Comment 9, 20, 28 |
|  |  | Is participant follow up close to everyday practice? | Comment 9, 20, 28 |

[1] Bassler D, Briel M, Montori VM, Lane M, Glasziou P, Zhou Q, et al. Stopping randomized trials early for benefit and estimation of treatment effects: systematic review and meta-regression analysis. Jama. 2010;303:1180-7.

[2] Higgins JP, Altman DG, Gotzsche PC, Juni P, Moher D, Oxman AD, et al. The Cochrane Collaboration's tool for assessing risk of bias in randomised trials. Bmj. 2011;343:d5928.

[3] Sterne JA, Hernan MA, Reeves BC, Savovic J, Berkman ND, Viswanathan M, et al. ROBINS-I: a tool for assessing risk of bias in non-randomised studies of interventions. Bmj. 2016;355:i4919.

[4] Whiting PF, Rutjes AW, Westwood ME, Mallett S, Deeks JJ, Reitsma JB, et al. QUADAS-2: a revised tool for the quality assessment of diagnostic accuracy studies. Ann Intern Med. 2011;155:529-36.

[5] Eldridge SM, Chan CL, Campbell MJ, Bond CM, Hopewell S, Thabane L, et al. CONSORT 2010 statement: extension to randomised pilot and feasibility trials. Pilot and Feasibility Studies. 2016;2:64.

**Detailed comments from survey participants, including answers by the authors**

| **Participant ID** | **Participant agrees on main question(s)** | **Comment by survey participant** | **Answer by the authors** |
| --- | --- | --- | --- |
| 6 | Yes | The grey part is good, but the examples (white part) go partially too far. Keep it as simple as possible and put only what is really needed ! | We agree that the current format may not be ideal to provide such level of detail. We would however like to keep the two levels within the framework (i.e. broader main questions and detailed items to exemplify content to the reader). In future applications, however, we envision the framework to be more interactive, e.g. including dropdown menus, increasing its usability. |
| 9 | Yes | Generalizability: this would not always be feasible, depending on Phase (e.g. Phase I, II) of the Project. Therefore it might be necessary to extend this item | We agree that this question is rather specific and have therefore rephrased the main question and items. However, we would like to point out that this framework only applies to research with patients, and does therefore not cover Phase I studies. |
| 13 | No | Access to data only indirectly covered by question re accordance with SPIRIT guideline and not covered by questions in Examples column. | We have added a main question on plans regarding dissemination of information and access to patient level data. |
| 15 | Yes | number of eligible study participants, not only, in Switzerland are often too low, but have to be min. > 50, better > 100, means muliticentered study | We agree that studies for example often suffer from recruitment problems leading to small sample sizes and underpowered studies. We have covered the precise estimation of sample size for an individual study in our questions on sample size calculation (“Precision” dimension) |
| 19 |  | Trial monitoring is a must! To consider only would be too weak. | We agree and have rephrased the main question on monitoring to “Is study monitoring (adapted to risk of study, if applicable) planned and documented in a monitoring plan?” |
| 20 | No | I have problems with two questions: Has feasibility been checked... I would prefer if it is moved from ethics to relevance. Generalizability: the question might call for exceptions as not all studies are designed to keep external validity as a precondition. | Whether a study is feasible or not predominantly affects the study participants. In case feasibility has not been thoroughly checked and e.g. leads to discontinuation of the study, this primarily has ethical implications for study participants who took risks and consented to take part in a study which was then discontinued due to poor planning. We therefore prefer to keep feasibility aspects in the “Protection of patient safety & rights” dimension. We agree though that feasibility touches on “relevance” and “precision” too and added therefore a reference to the ethics section in these dimensions.  In line with comment 2, we agree that the main question on generalizability is mainly applicable to interventional studies and have therefore rephrased the question. |
| 24 | No | Under Ethics: suggest including a question about benefit:risk balance. E.g. do the benefits outweigh the risks; are the risks kept as low as possible Under Transparency: is there a dissemination plan to share study information including the protocol, summary results, and participant level data? | We have additionally included a main question on the benefit-risk balance of the trial in both the concept and the planning stage.  We have also added a main question on plans regarding dissemination of information and access to patient level data. |
| 28 | No | - consider allowing as well other than SPIRIT guideline? How about ICH or FDA? - careful with "standard or routine" care in the international context, as the "standards" may highly differ by countries --> maybe the question about the generalisability should be rephrased? | We explicitly mention SPIRIT as a reporting guidance rather than other guidelines focusing on regulatory aspects. Regulatory compliance is covered in the “Protection of patient safety & rights” dimension. We, however, agree that SPIRIT is not the only guidance nor is it binding and therefore now only mention it as an example. We have further rephrased the main question in “Generalizability” as suggested. |
| 30 | No | What do you mean with pilot studies - most studies that can be summarized as "waste" are planned under the cover name "pilot" and sold as "gold" -> think carefully and do not use this term!! There is nothing like a pilot! | We agree that it is problematic if studies consider themselves pilots because they did not reach a pre-specified sample size. However, in our case pilots describe initial test/small studies to assess the feasibility of the actual, larger study (e.g. for realistic recruitment estimates) ^10^. We therefore believe that this type of studies would considerably improve feasibility of large studies if conducted in this context. We rephrased the question in order to put focus on the piloting aspect, and not the study type “pilot studies” themselves. |
| 31 | No | Ethics: see my comment at the beginning. I would not mix ethics and regulation e.g. what is the relationship between safety reporting according to legal requirements and ethics (I doubt that a trial that complies with safety reporting regulations is per se more ethical than a trial which does not comply)? Is feasibility part of ethics? Not more than any other dimension. Internal validity: I would provide this level of detail. It will never accommodate the individual situation. I would phrase it rather general. Transparency: I would not cite a particular guideline unless it is binding. Rather "... in accordance with established standards." | We agree that legal aspects are as important as ethical aspects and have therefore included both aspects within the renamed dimension “Protection of patients’ safety and rights”. Ultimately, both aspects aim at the protection of patients’ safety, rights, and wellbeing. We have further adapted our main question in the planning stage as follows in order to clarify for the reader: “Are patients’ safety and rights protected through the study’s adherence to applicable national and international regulations and laws?” and added the same question to the conduct stage. Whether a study is feasible or not predominantly affects the study participants. In case feasibility has not been thoroughly checked and e.g. leads to discontinuation of the study, we believe this primarily has ethical implications for study participants who took risks and consented to take part in a study which was then discontinued due to poor planning. We therefore prefer to keep feasibility aspects in the “Protection of patient safety & rights” dimension.  We further agree that the current format may not be ideal to provide such level of detail. In future applications, however, we envision the framework to be more interactive, e.g. including dropdown menus, increasing its usability.  We have rephrased the main question in “Transparency” as follows: Is the protocol in accordance with established standards (e.g. the SPIRIT or other applicable guidelines depending on study design)? |
| 32 | No | Dimension Ethics: Please add SUSARs as well :"Is collection, documentation and reporting of Adverse Events/Serious Adverse Events/SUSARs according to..." | We have added SUSARS to the main question. |
| 33 | No | See answer to question 5:  (*The design of the questionaire is not adequate. Yes/No options do not permit a balanced response. At least, the category "partially agree" should be introduced. I partially agree on the main questions: 1. the questions are too complicated/detailed 2. the questions are not weighted, i.e. the most important criterion "Novelty/potential gain of knowledge" needs to be no. 1.”*) | We added a general description of the framework in order to explain the content and meaning of each individual dimension in a separate file on “Framework Structure”. Within our “Relevance” dimension, we include novelty and innovation of research, but we also consider replication of studies valuable in case the existing evidence is not sufficient/conclusive. Our first question in the “Relevance” dimension expands on add-on value, replication, or novel areas of research. We explicitly refrain from weighting criteria as this may depend on the study design or the stakeholder applying the framework. All dimensions over all research stages are considered to be important. |
| 35 | Yes | not only SPIRIT, protocol depends on type of study; study procedures should be adaptable (not adapted) to routine clinical practice | We agree and have rephrased the question mentioning SPIRIT as an example of a guideline among others. |
| 36 | Yes | With respect to ethics please add qualification of investigators (CV, Research experience, GCP) and experience, important for instance for risk minimisation. | We fully agree that qualification of investigators is important, however, this is already covered under “Infrastructure” in our framework. |
| 43 | No | In my view, it is too far-reaching to expect trialists to plan knowledge transfer of future study results into a clinical guideline. In this regard, a more realistic expectation could be that study authors selected their outcome measures in a way that future meta-analyses can make use of study results. Trialists should adhere to core outcomes sets (if existing) and also measure outcomes preferably at the same time points as previous studies did. In the dimension of precision, the first two items are overlapping, because expected treatment effects are relevant for the first item but also affect the justification of sample size in the second question. The grey question on "recruitment procedures and monitoring" has little to do with statistical precision. This question is more relevant in the context of generalizability, because recording non-included patients helps to understand this aspect of research. | As required by the Canadian Institutes of Health Research (CIHR), for instance, all study proposals should consider knowledge transfer already at the planning stage. CIHR requires applicants to submit a plan for how they will translate their findings when the research is completed. We believe that this is a crucial aspect of relevance. However, we agree that it does not necessarily need to be an inclusion in a clinical guideline and have therefore adapted this main question.  We agree that trialists should choose outcome measures in a way that future meta-analyses can make use of the results. We have specified the choice of well-defined, valid outcomes (and core outcome sets) in the “concept” stage.  We agree that the first two questions in the precision dimension were overlapping. We have now integrated the second question into the first one.  Regarding recruitment procedures, we have adapted the main question in order to clarify that recruitment and monitoring of recruitment should be planned in order to increase chances to reach the target sample size. This in fact is an aspect of precision of study results. We cover the reporting of non-included patients and their characteristics in the conduct and analysis stage. |
| 44 | Yes | From the feasibility side...I would also add a point over can the research be completed in an appropriate timeframe. | We have included a “time aspect” in the question on whether recruitment assumptions are realistic. |
| 45 | No | This appears overly complex - could you simplify to just SPIRIT? | SPIRIT is a very valuable guide for protocol development and reporting. However, we feel that we need to include additional aspects which have to be taken into consideration at the planning stage of a study, e.g. the feasibility assessment, compliance with regulations, etc. |
| 47 | No | Under Ethics, some main points are missing. Issues like openness; guarding against unacceptable risks; human subjects protection In general try to avoid the word "trial" and use "study" instead | We have added a main question on benefit-risk balance of the trial both in the concept as well as in the planning stage. We now use “study” instead of “trial” throughout the framework (whenever applicable). |
| 49 | No | Relevance / Patient centeredness: contact guideline groups. Except for the large definitive outcome studies that are set up to solve a key issue in the management of a certain health problem, contacting guideline groups at that stage when the studiy has not yet started is too early. Certain topics come up at several stages, e.g. sample size calculation was already covered in the previous stage ... Is this done by purpose? ARe you requesting that the protocol should already be published at the time you start the study? Many researchers write up the study protocol for publication when the study has been launched. and publication may take 6 months or so ... | We agree that, for example, in an early Phase II study, contacting guideline groups is not indicated. This is why we explicitly provide it as an example item, rather than a main question. We, however, believe that it is important to keep it in the framework to stimulate thoughts in this direction early in the planning stage of a study.  Sample size calculation is covered in the concept stage (e.g. 50 vs. 3000 patients required) for a first rough feasibility assessment. In the planning stage, we would expect a more precise estimate of the sample size.  Regarding the study protocol, it would be ideal to publish it before study start. We, however, understand that this is not feasible due to many factors. We therefore adapted the question as follows: Is publication and accessibility of full study protocol planned? |

| **Study Stage III: Conduct**  **Milestone: Last patient last visit** | | |  |
| --- | --- | --- | --- |
|  | | |  |
| **Dimension** | **Specific question** | **Examples** | **References to comments** |
| **Protection of patient safety & rights** | Is respect for and consideration of patient rights, well-being and dignity guaranteed throughout conduct of study ? | Are study participants respected at all times, i.e.: |  |
|  |  | Is withdrawal from study at any time explicitly permitted? |  |
|  |  |  | Comment 31 |
|  |  | Are study participants informed about purpose of research, its procedures (including study medication, concomitant medication, emergency management, etc.) and potential risks, benefits and alternatives, so that they can make a voluntary decision? | Comment 36 |
|  |  | In case of routinely collected data (including biological material), are study participants informed about the further use of their data for research purposes? | Comment 42 |
|  |  |  | Comment 31 |
|  |  | Is study participants’ privacy and confidentiality ensured during (and after) study, e.g. through appropriate coding? |  |
|  | Is patient safety guaranteed throughout conduct of study? | Are study participants informed of newly discovered risks? | Comment 31 |
|  |  | Are side effects / Adverse Events/ Serious Adverse Events/ Suspected Unexpected Serious Adverse Reactions etc. monitored and reported to the ethics committee within required timeframes? | Comment 31 |
|  | Is the study being conducted according to protocol? |  | Comment 43, 30, 35 |
|  | Is there monitoring of compliance of participants and study staff with the protocol? |  | Comment 43,30, 35 |
|  | Are patients’ safety and rights protected through the study’s adherence to applicable national and international regulations and laws? |  | Comment 31 in “Structure” |
| **Relevance /**  **Patient centeredness & involvement** | Are there any measures in place to assure study participants’ involvement,cooperation, and feedback throughout conduct of study (e.g. incentives, phone calls, etc.)? |  | Comment 9, 12 |
| **Minimization of bias (internal validity)** | Are data systematically collected as pre-specified in the protocol? |  | Comment 45 |
|  |  | Are losses to follow-up minimized? |  |
|  |  | Are protocol deviations documented, and reported to the respective institutions? |  |
|  |  | Are changes in study procedures amended in the protocol? |  |
|  |  | Are reasons for withdrawing from the study/dropping out collected for such patients? |  |
|  |  | Are missing data documented by individual outcomes? |  |
|  |  | Apart from the allocated treatment, are study groups treated equally (e.g. no additional treatments or tests)? |  |
|  |  | If applicable, are study participants and clinicians kept "blind" to which treatment was being received? |  |
|  | Is monitoring being conducted according to the pre-specified monitoring plan? |  |  |
| **Precision** | Is enrollment of study participants monitored? | Are formal techniques in place to monitor recruitment centrally and at participating sites? |  |
|  |  | Are measures in place to allow timely reaction in case recruitment deviates from expectations? |  |
|  | Is variability of study procedures and measurement error minimized, e.g. by using centralized monitoring strategies? |  | Comment 43, 30, 35 |
|  |  |  |  |
| **Transparency / Access to data** | Is study conduct transparent to all involved parties? | Are protocol amendments or any necessary deviations from the original protocol clearly documented and disseminated to appropriate parties within reporting timelines? | Personal communication by expert 31 |
|  |  | Are internal or external audits planned, conducted and reported? |  |
|  |  | Is an external and independent Data Monitoring Committee present, or reason provided, why it is not needed? |  |
| **Generalizability (external validity)** | Are numbers of participants through different stages of a study documented (patient flow) including reasons for leaving the study prematurely (if voluntarily provided by patients)? | Is proportion of study participants who declined randomization documented? |  |
|  |  | Are the reasons for participants leaving the study before its scheduled end documented? |  |

**Detailed comments from survey participants, including answers by the authors**

| **Participant ID** | **Participant agrees on main question(s)** | **Comment by survey participant** | **Answer by the authors** |
| --- | --- | --- | --- |
| 3 | No | Maybe here, maybe later, maybe I have missed it: Interim Analysis or adaptive designs do not seem to be reflected in this Framework (ethical and statistical implications when stopping a trial prematurely) | We agree that this aspect was missing and we have added a statement on measures to prevent premature trial discontinuation for inadequate reasons and a statement on interim analysis/study termination (if required) in the” planning” stage of our framework. |
| 9 | Yes | Relevance: Incentives and phone calls? There might be better examples and measures | We would be happy to include better examples here if the expert has any suggestions. |
| 12 | No | Relevance / patient centeredness: add "feedback": ....participant's feedback, participation and cooperation... | We agree and have added “feedback” to the relevance question. |
| 15 | Yes | minimization bias, there are more than 2 groups as reporting bias, patient group exclusion bias and others | We agree that there are multiple forms of biases, but we have listed the most prominent ones as examples. |
| 20 | Yes | Is trial transparent ? Trasperency might be ensured but when the research is subject to proprietary agreement, access is at risk. So research can be fully transparent but totally inaccessible (e.g. Data owned by drug companies or agencies). | We fully agree that the aspect of access to data is of importance. We have therefore included main questions in the reporting and dissemination stage in order to foster open access data. |
| 26 | Yes | Is the respect of pharmacovigilance data sharing with co-investigators, patients, planed to be checked? | We fully support any kind of data sharing within this framework, also for pharmacovigilance. This is also ensured by our main question on study monitoring. |
| 30 | No | Main question under Precision are important but in the wrong dimension for me they would be part of the minimization of bias | We agree that monitoring protocol compliance is relevant for several dimensions but we feel that it primarily affects patient safety in addition to precision of the data collected. We have therefore moved this question to the “Protection of patient safety and rights” dimension. We further added a main question on whether the variability of study procedures and measurements is minimized in order to maximize precision. |
| 31 | No | Ethics: Maybe separate rights/dignity etc. and safety into two questions? Patient Relevance: question is rather related to internal validity. Internal validity: I would not list each individual potential bias or you would need to add (much) more ... Regarding monitoring: What if the pre-specified monitoring plan was wrong? Simply complying to it because it was pre-specified is not a good idea (but rather a sign of low planning quality but good conduct quality ...). Precision: precision is not only related to the number of participants (event rate, variance, measurement error, ...). Generalizability: I do not think the question is of relevance for generalizability. | We agree that the aspect of patient safety should be considered as an individual main question and have therefore separated the questions on patient rights and safety. We further agree that the bias questions should rather be listed as examples of a larger variety of potential biases than as main questions. Regarding monitoring, we agree that it can be only as good as the monitoring plan. However, this is covered in the planning stage and not in the conduct stage. Regarding precision, we have added a question on whether measurements and procedures are conducted in an accurate and reliable way. |
| 33 | No | See answer to question 5:  (*The design of the questionaire is not adequate. Yes/No options do not permit a balanced response. At least, the category "partially agree" should be introduced. I partially agree on the main questions: 1. the questions are too complicated/detailed 2. the questions are not weighted, i.e. the most important criterion "Novelty/potential gain of knowledge" needs to be no. 1.”*) | We added a general description of the framework in order to explain the content and meaning of each individual dimension in a separate file on “Framework Structure”. Within our “Relevance” dimension, we include novelty and innovation of research, but we also consider replication of studies valuable in case the existing evidence is not sufficient/conclusive. Our first question in the “Relevance” dimension expands on add-on value, replication, or novel areas of research. We explicitly refrain from weighting criteria as this may depend on the study design or the stakeholder applying the framework. All dimensions over all research stages are considered to be important. |
| 35 | Yes | point listed under relevance does not seem to belong here (rather feasibility issue); difficult to distinguish btw minimization of bias and precision; point under "generalizability" could also be listed under bias minimization | As the dimension is also called “…patient involvement”, we believe that the aspect of engaging patients fully fits into this dimension. We agree that the questions in “minimization of bias” and “precision” were difficult to distinguish and have now adapted the main questions and items. We further agree that the main point under “generalizability” is to some extent applicable to “minimization of bias”, however, the focus here is on the reasons for which the patients left the study and whether they are documented. |
| 36 | Yes | With respect to ethics it should be mentioned: allowed concomitant medication (emergency Management), individual discontinuation criteria, Interim analyses | We have adapted the main question as follows: “Are study participants informed about purpose of research, its procedures (including study medication, concomitant medication, emergency management, etc.) and potential risks, benefits and alternatives, so that they can make a voluntary decision?” |
| 42 | No | ethics dimension: Consent should be sought for the use of collected data and biological material for further research (already at the beginning of the study). Participants should be informed about the fate of the data/biol. material once the study ends. Is it shared with other institutions? With which ones? Exported abroad? Sold to teh pharmaceutical industry? etc.... | We have now included an item on further research use of routinely collected data and biological samples within the “Protection of patient safety & rights” dimension. |
| 43 | No | In my experience, the dimension of patient-centeredness cannot be fulfilled when the trial is running. During trial conduct, patients are in a fully passive role. The monitoring of protocol compliance is relevant for several dimensions. In the first place, violation of the protocol affects safety of participants, so this main question should be moved to this dimension. In my view, one main question could be added: "Are mechanisms established which allow early trial termination when required and prevent early trial termination for inadequate reasons?" | We believe that patient collaboration, involvement, and feedback should ideally be fostered throughout the conduct of the study, e.g. through phone calls, incentives, etc . We agree that monitoring protocol compliance is relevant for several dimensions and affects primarily patient safety in addition to the precision of the data collected. We have therefore moved this question to the “Protection of patient safety & rights” dimension. We further agree with your comment regarding trial termination and have included it as a main question in the planning stage of our framework. Ideally, measures/plans for trial discontinuation are established during the planning stage. |
| 45 | No | These questions are mostly for clinical trials, not other clinical research, particularly the bias ones. | We agree that the bias questions were too specific and have therefore kept the main question more general and included the specific bias items as examples. We, however, do not believe that the other main questions are trial-specific but rather apply to all types of clinical research. |
| 49 | Yes | Attrition bias: I do not understand the example: Do reasons for dropping out have an impact of the assessment of compliance, effectiveness and safety ? | We changed the question to the following: “Are reasons for withdrawing from the study/dropping out collected for such patients?” |
| 54 | Yes | Ethics&patient safety during conduct stage: maybe mention the oversight of the PI during the study. | We agree that oversight of the PI may be an important aspect, however, we would switch perspective within the framework. Further, the training of the PI, and the documentation thereof, is covered within the two promoters “Infrastructure” and “Education”. |

| **Study Stage IV: Analysis & Interpretation**  **Milestone: Study data analyzed and interpreted** | | |  |
| --- | --- | --- | --- |
|  | | |  |
| **Dimension** | **Specific question** | **Examples** | **References to comments** |
| **Protection of patient safety & rights** | Does data sharing adhere to appropriate data protection policies? | Is patient-level data anonymized? | Comment 21 in “final comments” |
|  |  | Have other risks for re-identifying participants been minimized? | Comment 21 in “final comments” |
| **Relevance /**  **Patient centeredness** | Are data analyzed to maximize the use of results by different stakeholders? | Are confidence intervals calculated on an absolute scale to gauge the benefit of an intervention for decision makers (e.g. clinicians, patients, policy makers)? | Comment 31 |
| **Minimization of bias**  **(internal validity)** | Are the data analyzed as pre-specified in the protocol/statistical analysis plan? |  | Comment 13 |
|  |  | Are post-hoc analyses clearly labelled as such or as exploratory analyses? | Comment 30 |
|  |  | Is data analysis performed using standard, generally accepted software? |  |
|  |  | Are data assumptions checked (e.g. normal distribution) as appropriate for planned statistical tests/modelling? |  |
|  | Has there been statistical adjustment using key confounding variables in the analysis (e.g. multivariable analysis), if applicable? |  | Personal communication by expert 13 |
|  | Does the analysis follow an adequate strategy to deal with participants in whom treatment or follow-up was not in accordance with study protocol? | Is the intention-to-treat principle followed (i.e. study participants were analyzed in groups as randomized) in case of a superiority hypothesis? | Comments 13, 30, 43, 45 |
|  |  | Are both a per-protocol and an analysis following the intention-to-treat principle conducted in case of a non-inferiority hypothesis? | Comments 13, 30, 43, 45 |
|  |  |  |  |
|  | Have results been interpreted with least possible “spin”? (e.g. without intentionally implying greater or lesser effects than have actually been shown by the data)? |  | Comment 3, 16, 28 |
| **Precision** | Is the uncertainty of results considered in the analysis? | Are confidence intervals or other measures of uncertainty calculated? | Comment 31 |
|  |  | Are reasonable sensitivity analyses for missing data conducted? | Comment 31 |
|  |  | Does interpretation adequately reflect uncertainty? | Comment 31 |
| **Transparency / Access to data** | Is the analysis code clearly documented, and is the analysis process reproducible? |  |  |
|  | Are deviations from the statistical analysis plan or protocol adequately documented and reported? |  | Personal communication by expert 30 and 31 |
| **Generalizability**  **(external validity)** | Does the interpretation put the results adequately into context of clinical practice/public health? |  | Comment 13, 31 |

**Detailed comments from survey participants, including answers by the authors**

| **Participant ID** | **Participant agrees on main question(s)** | **Comment by survey participant** | **Answer by the authors** |
| --- | --- | --- | --- |
| 3 | No | See previous comment with regard to Interim Analysis and/or adaptive designs | We have added a main question on mechanisms to prevent early trial discontinuation for inadequate reasons and trial termination if required in the planning stage. Example items cover interim analysis. |
| 13 | No | (1) Same question in both columns: "Is (the) data analyzed..." (2) Wording for "min of bias" is either specific for observational studies (adjustment for confounding var's) or trials (ITT) but it is not clear which one applies when or whether all questions listed should apply. | We agree that ITT and PP questions are too specific for trials and have listed them as examples, rather than main questions. |
| 14 | Yes | Although I agree with the questions, I think the question "Are results interpreted without "spin"?" could be phrased better! | We have adapted our questions on “spin” (i.e. to minimize rather than avoid it) and have clarified its meaning. |
| 16 | No | I don't believe that results can be interpreted without "spin". To guard against this, data need to be open access - this way other researchers can verify Interpretation and confirm results. How these are then placed within the context of existing research is always associated with "spin". This is why, in writing up, there is a results and a discussion section. | We have adapted our questions on “spin” (i.e. to minimize rather than avoid it) and we cover open access to data within the transparency domain of the reporting stage. |
| 20 | No | I find the quality framework much more structured around statistical issues than other aspects, being unbalanced. As an example take transparency: it only mention the analysis code. There are potentially important items that are overlooked, such as prioritization, outcome (process and patient centered) and comparators selections, or the balance between multiple dimensions (e.g. feasibility and power). In the end the risk is that the framework might support studies that are not that good. | We agree that other items, such as prioritization, outcome, and comparator selection are highly important which is why we cover them in different aspects in our framework. In the analysis stage, we believe that it is predominantly crucial to clearly document the analysis code and make it accessible. However, in the next stage, the reporting stage, we of course encourage many more items to be transparently disseminated. |
| 28 | Yes | what does "without spin" mean? | We have adapted our questions on “spin” (i.e. to minimize rather than avoid it) and have clarified its meaning. |
| 30 | No | I think data should not only be analyzed as in the protocol specified but there must be a document with many more details like an analysis plan. I think IIT should and PP should not be part of the main questions because they are study type specific. | We agree that in addition to the protocol, there ideally is an analysis plan as described in the planning stage. We therefore adapted this main question. We further moved IIT and PP to the examples. |
| 31 | No | Relevance: I do not think this domain is relevant at this stage Bias: Being compliant with pre-specified analysis is one thing whether the pre-specified analysis is meaningful and good is another. So this is a (very) low indicator for quality. I am not sure whether I would use such specific questions. Whether interpretation is adequate relates rather to generalizability and is not internal validity. Precision: Question too specific from my point of view. You do not want the framework to be a quality assessment tool do you??? I would rather ask: is uncertainty appropriately shown and addressed. Generalizability: Does the interpretation put the results adequately into context? | We agree that our previous main question did not fully cover the intended relevance aspect. We have rephrased such that the analysis should be conducted in a way that it makes the results useful and usable for decision makers (e.g. by providing confidence intervals).  We fully agree that being compliant with a pre-specified analysis plan is only helpful if the analysis plan is of good quality. We consider this aspect in the planning stage of our framework. We have rephrased our main question in precision according to your suggestion and added your suggested question on generalizability. |
| 33 | No | Good: relevance is on top of the list. Not so good: A clinical study can deliver novel knowledge other than "clinically meaningful Treatment effects" | We agree that there may be more than just “clinically meaningful treatment effects” and have rephrased the question such that it is of main importance whether the study results are useful for decision makers, i.e. clinicians, policy makers, and patients. |
| 36 | Yes | Valid analyses are also part of ethical judgment. | We agree that valid analyses are part of ethical judgment. The listed dimensions are not mutually exclusive, but we tried to limit the overlap. If the overlap was considered substantial we collapsed dimensions. Despite the overlap we felt that validity aspects deserve own dimensions other than “Protection of patient safety & rights”. |
| 43 | Yes | The two questions on ITT and PP analysis could be merged together: "Does the analysis follow an adequate strategy to deal with patients in whom treatment or follow-up was not in accordance to study protocol?" As an additional aspect, data ownership could be added: "Are data owned by the original investigator team?" | We have added your question as suggested. In addition, we agree that ITT and PP questions are too specific for trials and have listed them as examples, rather than main questions. |
| 45 | No | Some, but again these are clinical trials specific (eg ITT) | We agree that ITT and PP questions are too specific for trials and have listed them as examples, rather than main questions. |

| **Study Stage V: Reporting & Knowledge Translation**  **Milestone: Study archived and published** | | |  |
| --- | --- | --- | --- |
|  | | |  |
| **Dimension** | **Specific question** | **Examples** | **References to comments** |
| **Protection of patient safety & rights** | Is study completion/termination communicated to appropriate parties and documented in registries? | Is study completion/termination reported to ethics committee/regulatory bodies? |  |
|  |  | Is study completion/termination appropriately documented in national/international registry? |  |
|  | Are study participants informed about the outcome/main findings of the study in plain language (including treatment allocation of participant, if applicable)? |  | Comment 5, 13, 15, 28, 42, 47, 54 |
|  | Do study participants get access to products/interventions after study, if applicable? |  | Comment 43 |
| **Relevance /**  **Patient centeredness & involvement** | Do authors critically reflect on research findings (results as well as challenges or mistakes during study conduct) and the implications for future research? |  |  |
|  | Is the study easily available to decision/policy/guideline makers? | Is the study cited in a clinical guideline? |  |
|  | Are study patients/patient representatives involved in reporting the study? |  | Comment 5,13,28, 42, 47 |
|  |  | Are patient representatives involved in reporting of the study, e.g. in writing of lay term summaries? | Comment 35, 43 |
|  |  |  |  |
| **Minimization of bias**  **(internal validity)** | Are all outcomes and important study characteristics reported, as pre-specified in the protocol (outcome reporting bias prevented)? | Are all patient-relevant outcomes reported as pre-specified in the protocol? |  |
|  |  | Are important modifications to the protocol (e.g. premature discontinuation) reported (if applicable)? |  |
| **Precision** | Are absolute and relative treatment effects reported accompanied by confidence intervals? |  |  |
|  | Is the analysis set of participants clearly specified? | Are the actual numbers of recruited, randomized (if applicable), followed-up, and analyzed participants reported for each outcome and for each treatment group (if applicable)? |  |
| **Transparency / Access to data** | Is dissemination of data and study results maximized? | Is dissemination maximized through open access? |  |
|  |  | Is anonymized individual participant-level data made available (data sharing)? |  |
|  |  | Are study results posted in study registries? |  |
|  |  | Does publication in journals include full protocol and statistical analysis plan? |  |
|  |  | Is dissemination maximized through use of alternative media other than medical journals? |  |
|  |  | Are resulting doctoral/master theses made publicly available (if applicable)? |  |
|  | Are reporting guidelines followed, to facilitate critical appraisal and reproducibility? | *Is reference made to reporting guidelines such as CONSORT (Randomised trials) ^5^, STROBE (Observational studies) ^6^, STARD (Diagnostic studies) ^7^, or PRISMA (Systematic reviews) ^8^ depending on the respective study design.* |  |
|  |  | Are detailed methods disclosed in publications (to enable reproducibility)? |  |
|  | Are selective reporting, “spin” and plagiarism avoided and conflicts of interest declared? | Is selective reporting of study results avoided? |  |
|  |  | Is plagiarism and self-plagiarism avoided? |  |
|  |  | Are the study results independently peer reviewed? |  |
|  |  | Is “spin” (i.e. reporting to convince readers that the beneficial effect of the experimental treatment is greater than shown by the results) minimized in reporting of results? | Comment 28 |
|  |  | Were conflicts of interest declared? |  |
|  | Is knowledge transfer & exchange fostered? | Is knowledge transfer & exchange fostered through e.g.: |  |
|  |  | Community and provider education and outreach |  |
|  |  | Facilitation of two-way communication (lay language) with diverse populations and community groups |  |
|  |  | Knowledge transfer & exchange among clinical research groups |  |
|  | Are study records and data sets kept and archived for at least the legally required period of time? |  | Personal communication by expert 13 |
| **Generalizability**  **(external validity)** | Is potential impact on clinical practice / public health outlined in publicly accessible research reports (e.g. journal publication)? |  | Comment 13, 39 |
|  | Are characteristics of included participants clearly reported? | Are inclusion and exclusion criteria clearly reported? |  |
|  |  | Are characteristics of included participants clearly reported? |  |
|  | Are results of pre-specified subgroup analyses, if applicable, reported to help assess the importance of key participant characteristics (e.g. disease severity, age or gender)? |  | Comment 43 |

[1] Altman DG, Schulz KF, Moher D, Egger M, Davidoff F, Elbourne D, et al. The revised CONSORT statement for reporting randomized trials: explanation and elaboration. Ann Intern Med. 2001;134:663-94.

[2] Vandenbroucke JP, von Elm E, Altman DG, Gotzsche PC, Mulrow CD, Pocock SJ, et al. Strengthening the Reporting of Observational Studies in Epidemiology (STROBE): explanation and elaboration. Int J Surg. 2014;12:1500-24.

[3] Cohen JF, Korevaar DA, Altman DG, Bruns DE, Gatsonis CA, Hooft L, et al. STARD 2015 guidelines for reporting diagnostic accuracy studies: explanation and elaboration. BMJ Open. 2016;6:e012799.

[4] Liberati A, Altman DG, Tetzlaff J, Mulrow C, Gotzsche PC, Ioannidis JP, et al. The PRISMA statement for reporting systematic reviews and meta-analyses of studies that evaluate health care interventions: explanation and elaboration. Ann Intern Med. 2009;151:W65-94.

**Detailed comments from survey participants, including answers by the authors**

| **Participant ID** | **Participant agrees on main question(s)** | **Comment by survey participant** | **Answer by the authors** |
| --- | --- | --- | --- |
| 5 | Yes | In Access to data: one may need to address specify the 'open access' issue Also not specified is communication of results to the involved study participants. Maybe also to specify the issue of clinically relevant findings, how to report? | Open access is covered by the examples for main question “Was dissemination of data and study results maximized?” We have now added a main question on the dissemination of study results to patients in plain language. |
| 6 | No | Do you really mean that study participants should help to write a scientific paper ? - If yes then you have to include study participants representatives in the study planning and study procedure. But they have also conflicts of interests, and the independency of research cannot be guaranteed anymore. However, I agree that the legal and ethical guidelines have to be followed. But more ? | We cover main questions on patient involvement from study conceptualization through reporting. As we describe in our reporting question, we believe that patient representatives/advocates should be involved in the reporting of the study, specifically for easily understandable plain language summaries and the reporting of study results to study participants. |
| 13 | No | (1) What does "study archived" mean? Database locked or something else? (2) Whether participants were *involved* in reporting is quite different from example question whether they were informed. (3) "Did results impact clin. practice?" Of course, this is highly desirable but it can only be assessed post hoc and depends not only on the research itself. | Re (1): All study-related documents and important information need to be archived up to 15 years, depending on the regulatory environment (at least 2 years for GCP). Re (2): We agree that these are two separate points. We have added a main question on whether patients were informed about the main study outcomes. To (3): We agree that assessing the impact of a study can only be assessed post hoc and does therefore not fit in the reporting stage of the study. We have rephrased the main question such that the potential impact of the study on practice/public health should at least be outlined and discussed in the final study report and the publication. |
| 14 | Yes | Excellent questions as many of these are forgotten or at least minimized at the publishing stage. | Thank you very much! |
| 15 | Yes | for presentation of statistics, public need is a standardised, explained and clear "fact-box" | We have included a main question on whether patients receive information about the study outcome in plain language, which may for example include a “fact-box” for presentation of statistics. |
| 20 | No | In this section I find 'relevance' better structured and more complete than in previous stages. I find some items such as CoI equally important (or more important) at earlier stages (i.e. Planning). Some questions are presuntive: maximal dissemination or impact on practice. Dissemination and changes require time. Even if planned these activities can only be assessed some time after the research has been finalized. | We agree that assessing the impact of a study can only be assessed post hoc and does therefore not fit in the reporting stage of the study. We have rephrased the main question such that the potential impact of the study on practice/public health should at least be outlined and discussed in the final study report and the publication. |
| 28 | Yes | - again I am unsure what you mean with "without spin"? would there be a more appropriate expression to use? - miss as well the explicit concept of "lay summary or plain language of results" | Spin is a specific way of reporting to convince readers that for example the beneficial effect (efficacy, safety) of the experimental treatment is higher than shown by the results. We have rephrased the question and added this explanation.  We have now added a main question on the dissemination of study results to patients in plain language. |
| 31 | No | Again, somewhat too detailed and specific. Some of the question rather relate to the previous stage. Generalizability: Did results impact clinical practice is not feasible to assess in this framework I assume. | We agree that assessing the impact of a study can only be assessed post hoc and does therefore not fit in the reporting stage of the study. We have rephrased the main question such that the potential impact of the study on practice/public health should at least be outlined and discussed in the final study report and the publication. |
| 33 | No | Again, partially agree. The main consideration at this stage should again focus on the gain of novel knowledge. | After the conduct of the study, it may be difficult to adapt its „novelty content“. We therefore focus at this stage on the transparent reporting of the study results (negative or positive) to the applicable stakeholders. |
| 35 | Yes | availability to policy/guideline makers does not seem to be a relevance issue but rather related to transparency (likewise disemination of data, open access); "access to products/interventions after end of trial" might not always be feasible/ethical | We have added “if applicable” to the question on “access to products/interventions” because we agree that this is not feasible under all circumstances and only applies to certain study types. |
| 39 | No | in fact all fine except for generalizability also the dimensions of public health ethics Need to be considered. Generalizability is here too narowly defined.... | We adapted the question in “Generalizability” as follows: Is potential impact on clinical practice / public health outlined in research report / publication? |
| 42 | No | Ethics dimension: Are study participants informed about the main findings of the study? Results should be reported to study participants in plain language. Results relevant for patients should be reported independently of whether they wished to be informed or not. | Whether participants were informed about the main findings was covered under “examples” but has now been rephrased into a main question. |
| 43 | No | The question whether study participants did get access to study interventions after the trial had little to do with the relevance of the trial. This question is more closely related to ethics. With regard to generalizability, it might be recommended that researchers report subgroup analyses in order to assess the importance of key patient characteristics, such as disease severity, age or gender. In addition, it might be helpful in each study to compare included patients with non-included or non-eligible patients. The expectation is too high when it comes to the Impact of the study. At this stage, when results are published it is unlikely that clinical guidelines of clinical practice changes immediately. Usually, such changes are hard to measure and take several years. | We agree that if a study is conducted without considering access to the potentially beneficial intervention/treatment after its end, it is unethical. We have therefore moved this question to the “Protection of patient safety & rights” dimension.  With regard to generalizability, we have added your suggested main question on sub-group analyses.  We agree that assessing the impact of a study can only be assessed post hoc and does therefore not fit in the reporting stage of the study. We have rephrased the main question such that the potential impact of the study on practice/public health should at least be outlined and discussed in the final study report and the publication. |
| 47 | No | Under ethics: have patients been informed about their allocation in case of a study with blinding? | We have included this question in our “Protection of patient safety & rights” dimension. |
| 54 | Yes | Ethics: does the result affect other currently ongoing or planned trials? Transparency: (how) will the participants be informed about the results? | We agree that patient should be informed about the results of the trial in plain language and have therefore added a main question to the “Protection of patient safety and rights” dimension. We are not sure whether affecting or not affecting other currently ongoing or planned trials has an impact on clinical research quality. What is your rationale for this? |

| **Sustainability / Education** | |  |
| --- | --- | --- |
|  | |  |
| **Specific question** | **Examples** | **Reference to comments** |
| Are doctoral students, junior researchers, clinicians, or patient advocates actively involved in all stages of a clinical study, reliably supervised/mentored by senior researchers, and are their specific contributions acknowledged appropriately? | Are doctoral students, junior researchers, clinicians, or patient advocates actively involved in study design, planning, conduct, analysis, interpretation and dissemination of results (e.g. publications, conference presentations, reports, or lay summaries)? | Comment 3,15,and 31 |
|  | Are doctoral students, junior researchers, or clinicians actively supervised by senior researchers at all stages of a clinical study? |  |
|  | Are doctoral students, junior researchers, or clinicians mentored as to career options in clinical research (early career development)? |  |
|  | Are training options and courses in health research methodology available for principal investigators, staff, and patient advocates? | Comment 3, 15,and 31 |
|  | Are doctoral students, junior researchers, or clinicians mentored to improve awareness about value of clinical research to patients and society as a whole? |  |
|  | Are processes continuously adapted and improved to changes, developments, issues, and conditions during research continuum (quality by design)? |  |

**Detailed comments from survey participants, including answers by the authors**

| **Participant ID** | **Participant agrees on main question(s)** | **Comment by survey participant** | **Answer by the authors** |
| --- | --- | --- | --- |
| 3 | No | Add: ... and their specific contributions acknowledged appropriately. | We added the suggested part. |
| 15 | No | why not also patient advocates? | We agree that this section should also include training opportunities for patient advocates and have rephrased the question. |
| 20 | No | Most trials are done outside the academia. | Even if many trials are sponsored by industry, the conduct of the studies (at least after phase I) is dependent on trial personnel at academic institutions, including Principal investigators, and their staff. We therefore do not fully agree that this personnel should not be trained and educated well, so that all types of studies, independent of the sponsor, are conducted well. |
| 31 | No | Is all (!) personnel involved in clinical research i.e. including the seniors regularly trained? | We agree that all personnel need to be trained, which is included in our “Infrastructure” section as well as covered within the Education dimension under “Are training options and courses in health research methodology available for principal investigators, staff, and patient advocates?” |
| 36 | Yes | With all involved persons that are not employed by the Institution (e.g., doctoral students) an Agreement should be in place with respect to privacy and secrecy. | We agree that this is an important aspect and covered it in the “infrastructure” promoter as follows: “Are adequate facilities ensuring data security and privacy in place (incl. competent and effective IT support to facilitate solutions tailored to specific challenges of individual studies or agreement templates for doctoral students with respect to data privacy and confidentiality)?” |
| 43 | No | According to my experience, the financial sustainability of clinical research is extremely important, and many research institutions are at stake due to overoptimistic research planning. I suggest adding a question on this issue: "Is financial sustainability of the research unit paid attention to?" Possible subquestions could address the existence of financial monitoring, planning of marketing for successful trial interventions, and preparation of subsequent studies. | We agree that this aspect is of importance. We however do not believe it should be included in the “education” section. We have covered questions related to this aspect in the “Planning & Feasibility” stage as well as under “Infrastructure”. |
| 45 | No | Good idea, but not mandatory for all clinical research | We agree that this is not a mandatory aspect of clinical research, but as we construct a framework for the “ideal” quality of clinical research, we still think it should be included. We think that training and teaching good research practice to junior staff is an important step for an institution to successfully improve overall quality of clinical research. |
| 46 | No | I don't disagree with the principle here but I wonder if there might be situations whereby junior researcher involvement is not applicable ? | We agree that this is not a mandatory aspect of clinical research, but as we construct a framework for the “ideal” quality of clinical research, we still think it should be included. We think that training and teaching good research practice to junior staff is an important step for an institution to successfully improve overall quality of clinical research. |

| **Infrastructure** |  |  |
| --- | --- | --- |
|  | |  |
| **Specific question** | **Examples** | **Reference to comments** |
| Is a Quality Management System incl. Standard Operating Procedures (SOPs) in place? | Is all staff continuously trained in applicable SOPs? |  |
|  | Are there measures in place to control, whether the existing Quality Management System is followed? (i.e. internal audits) |  |
| Are well-trained,experienced, and dedicated principal investigators and study staff present? | Has the principal investigator and/or staff been involved in clinical studies before? |  |
|  | Is all staff continuously trained in GCP and protocol-related activities, and particularly the informed consent process? | Comment 20, 42 |
|  | Is training (e.g. GCP) of each participating investigator and staff member clearly documented? |  |
|  | Are roles and responsibilities of each participating investigator and staff member clearly documented? |  |
|  | Are all involved stakeholders well and adequately informed about study procedures and changes? |  |
| Are expert epidemiologists/methodologists, statisticians, professional data managers, and/or a logistical support unit involved early-on? | Are epidemiologists/methodological specialists involved in development of protocol? |  |
|  | Are statisticians involved in development of protocol? |  |
|  | Are data managers involved in the development of the data management plan and the setup of the data management system? |  |
|  | Is a logistical support unit involved in study planning and/or conduct, e.g. through regulatory affairs experts, study nurses, or project managers? |  |
| Are adequate human, material, and equipment resources available for study conduct? | Is dispense, transport, and storage of investigational medicinal product, if applicable, planned? |  |
|  | Is availability of study-specific materials, hardware, and facilities planned and secured? |  |
|  | Is a transparent study budget available and approved by experienced personnel, including costs for experts mentioned above? |  |
|  | Is funding secured through acquisition of competitive money or through collaboration with e.g. industry partners? |  |
| Are adequate facilities ensuring data security and privacy in place (incl. competent and effective IT support to facilitate solutions tailored to specific challenges of individual studies or agreement templates for doctoral students with respect to data privacy and confidentiality)? | Is an electronic database incl. audit trail in place? |  |
|  | Is participant data coded? |  |
|  | Is IT support present at site? |  |
| Is inter-/multidisciplinary collaboration and involvement in study planning and conduct fostered? | Have all relevant stakeholders been involved in protocol development and conduct? (e.g. investigators at other study sites, etc.) |  |
|  | Is communication between involved staff, sponsor, contractors, and site fostered? |  |
| Are all institutions involved in the study covered by compulsory liability insurance? |  | Comment 13 |
| Is an overview of the existing research infrastructure available and accessible to any researchers with a study idea? |  | Comment 14 |

**Detailed comments from survey participants, including answers by the authors**

| **Participant ID** | **Participant agrees on main question(s)** | **Comment by survey participant** | **Answer by the authors** |
| --- | --- | --- | --- |
| 3 | No | Data Archiving and policies for data deletion or consent withdrawal should also be addressed here. Also data sharing policies should be addressed. | We have covered data sharing policies/ethics and data archiving in the reporting stage of the framework. |
| 5 | Yes | methodological specialists: is here bioinformatics included, or need they to be mentioned | Yes, we include bioinformatics as an example of methodological / statistical experts. |
| 13 | No | Rephrase last question to make applicable to just one given study (not in general). | We agree and have adapted the last question as suggested. |
| 14 | Yes | Perhaps a general question about whether you can adequately describe or represent the infrastructure? i.e. show simply who is involved and what they do..? | The infrastructure of individual studies or research groups should be adequately documented as part of an existing quality management system. With respect to guidance/assistance of unexperienced researchers with study ideas we added the following question: “Is an overview of the existing research infrastructure available and accessible to any researchers with a study idea?” |
| 20 | No | This is the most annoying page as it presumes that a study can only be done with all listed resources/professionals. In other words there is a strong spin on richness. However richness does not make a study any better. I work in a place where almost everybody has been trained in GCP. Most people would not be able to design a good study even in years. I see RCTs where few people have some capacities and tech skills and a lot of support from local communities passionate and engaged: they do best RCTs in shorter times. Here formal training (usually in courses promoted by CROs) is receiving more attention than dedication and passion. | We fully agree that dedication (which we now mention in our main question on trained personnel) and passion are important drivers for successful clinical research. However, we also think that infrastructure, even if at less developed level as described in the framework, will help to achieve study continuity, completion, and therefore success. This section of the framework describes the ideal setting, and can also be referred to as a goal or “nice to have”. None of these items should be an obligation or fully mandatory, however, their existence will positively influence the quality of the study, together with many other aspects as described in the framework. |
| 31 | No | QM: I am not sure whether the presence of a QM or (some) SOPs is sufficient. Would it not be necessary to apply some criteria? Critical mass: What is the critical mass? Might be different for different situations. Not sure whether it is a quality dimension. Insurance: Is this part of the infrastructure? I would say no. Rather related to legal/regulatory compliance. What about project-specific quality control and assurance measures? For example, is there a systematic approach for project-specific quality management in place? I assume the first question relates to the institutional QM? | We have removed the term “critical mass” from the main question as we agree that its definition in itself is vague and depends on the observer. We have included insurance under “Infrastructure” as it is often covered either by the sponsoring institution, or in the academic setting by the hospitals. Regarding project-specific quality control, we have included items on monitoring and quality control in the planning and conduct stage of the framework. |
| 33 | Yes | BUT: "Main Quality" of a clinical study is not solely determined by the available infrastructure. NOTE: extent of infrastructure IS NOT EQUAL to research and scientific quality | We fully agree that infrastructure is not the main driver of quality, which is why we include it as a “quality promoter”. Main study quality is determined by the main quality dimensions we have listed, while good infrastructure is supporting the quality of a study positively. |
| 35 | Yes | point 2 and 5 are similar, could be combined; maybe separate into human and other ressources or basic infrastructure and specific infrastructure for a given trial | While point 2 addresses human resources, point 5 addresses infrastructural resources. We believe that all of these points could either be organized at institutional level or at individual trial level, but that they are all important to access at trial level. |
| 42 | No | It should be explicitly stated that suitable and specifically trained staff for the informed consent process is planned and allocated to the study. | We agree and have included as suggested. |
| 43 | Yes | In the question on adequate technical facilities, the focus is set on IT structure and support. In my view, paper-based data recording is still highly effective and thus should not be penalized here. In addition, not only the security and privacy of data but also the speed of data transfer is important (mainly for SAE reporting). | We agree that paper-based recording may still be necessary. Archiving is covered in the reporting stage. This is however not meant here; the IT structure exclusively aims to secure data that is collected electronically. We further agree that the speed for data transfer, such as SAE reporting, within given timeframes is crucial. This is already covered in the conduct stage within the “Protection of patient safety & rights” dimension. |

**References**

1. Higgins JP, Altman DG, Gotzsche PC, et al. The Cochrane Collaboration's tool for assessing risk of bias in randomised trials. *Bmj* 2011; **343**: d5928.

2. Sterne JA, Hernan MA, Reeves BC, et al. ROBINS-I: a tool for assessing risk of bias in non-randomised studies of interventions. *Bmj* 2016; **355**: i4919.

3. Whiting PF, Rutjes AW, Westwood ME, et al. QUADAS-2: a revised tool for the quality assessment of diagnostic accuracy studies. *Ann Intern Med* 2011; **155**(8): 529-36.

4. Chan AW, Tetzlaff JM, Gotzsche PC, et al. SPIRIT 2013 explanation and elaboration: guidance for protocols of clinical trials. *Bmj* 2013; **346**: e7586.

5. Altman DG, Schulz KF, Moher D, et al. The revised CONSORT statement for reporting randomized trials: explanation and elaboration. *Ann Intern Med* 2001; **134**(8): 663-94.

6. Vandenbroucke JP, von Elm E, Altman DG, et al. Strengthening the Reporting of Observational Studies in Epidemiology (STROBE): explanation and elaboration. *Int J Surg* 2014; **12**(12): 1500-24.

7. Cohen JF, Korevaar DA, Altman DG, et al. STARD 2015 guidelines for reporting diagnostic accuracy studies: explanation and elaboration. *BMJ Open* 2016; **6**(11): e012799.

8. Liberati A, Altman DG, Tetzlaff J, et al. The PRISMA statement for reporting systematic reviews and meta-analyses of studies that evaluate health care interventions: explanation and elaboration. *Ann Intern Med* 2009; **151**(4): W65-94.

9. Bassler D, Briel M, Montori VM, et al. Stopping randomized trials early for benefit and estimation of treatment effects: systematic review and meta-regression analysis. *Jama* 2010; **303**(12): 1180-7.

10. Eldridge SM, Chan CL, Campbell MJ, et al. CONSORT 2010 statement: extension to randomised pilot and feasibility trials. *Pilot and Feasibility Studies* 2016; **2**(1): 64.
