# Supplementary material for: Academic response to improving value and reducing waste: A comprehensive framework for INcreasing QUality In patient-oriented academic clinical REsearch (INQUIRE)
Source: PLoS Med. 2018 Jun 7;15(6):e1002580. doi: 10.1371/journal.pmed.1002580 (PMC5991651; doi:10.1371/journal.pmed.1002580)
Supplement: S4 Appendix — (DOCX) [file pmed.1002580.s004.docx]

**S4 Appendix**

**Full framework, including specific quality questions and descriptive examples**

| **Study Stage I: Concept**  **Milestone: Research question including study type defined and viable** | | |
| --- | --- | --- |
|  | | |
| **Dimension** | **Specific question** | **Examples** |
| **Protection of patient safety and rights** | Can the research question be addressed in the given setting? | Based on a rough resource assessment, and potentially available study participants, is it feasible to answer the research question? |
|  |  | Based on a rough budget estimate, is it feasible to answer the research question with a specified study type? |
|  | Does the study consider equity appropriately? | Are participants selected so that   - vulnerable individuals are neither targeted for risky research nor withheld from research relevant to these populations? - socially powerful individuals are not favored for potentially beneficial research? |
|  | Is the research design adequate for the stage of an investigated technology to ensure patient safety? | Are sufficient data on toxicity/teratogenicity of an intervention available from animal studies or phase I studies? |
|  | Do the (assumed) short- and long-term benefits of the study outweigh potential risks associated with the study (consistent with clinical equipoise)? |  |
| **Relevance/patient centeredness and involvement** | Is significant potential add-on value to existing evidence (systematic review) specified, taking into consideration burden of disease and anticipated benefit of treatment? | Are uncertainties in existing evidence identified and discussed in a systematic review? |
|  |  | Does research   - expand or challenge current knowledge? - open additional areas for new research activity? - justify replication of existing evidence, if applicable? |
|  | Are patient representatives/advocates and their needs and values involved in the development of the research question? |  |
|  | Are outcome measures patient-relevant? | Are outcomes patient-relevant according to COMET^1^, including quality of life, if applicable, and with judicious use of surrogate endpoints? |
| **Minimization of bias (internal validity)** | Is the selected study type/design appropriate to minimize bias? | Is the study randomized or, if not, appropriately controlled for confounding? |
|  | Are potential sources of bias anticipated, evaluating the magnitude and the likely direction? |  |
|  | Are outcome measures well-defined, prespecified, valid, reliable, and measured at appropriate times? | Are outcomes   - well-defined (up-front)? - valid (measure what they intend to measure)? - reliable (stable and consistent when repeatedly measured)? - sensitive to important change? - measured at appropriate times? - standardized across studies (core outcome sets, if applicable)? |
| **Precision** | Has an estimate of the required sample size been made (for feasibility purposes, see “Protection of patient safety and rights”)? |  |
| **Transparency/access to data** | Is the research question clearly specified (e.g., in a synopsis)? | Is each component of P(I/E)(C)O^1^ as applicable to study design clearly defined, i.e. :   - patient population to be recruited/investigated in the study, - intervention to be assessed, - exposure to be assessed, - diagnostic test to be assessed, - control intervention as comparator, and - outcomes to be measured? |
| **Generalizability (external validity)** | Are planned study participants representative of patients who would use the drug/intervention/diagnostic test in a real-life setting? | Are unnecessary restrictions through inclusion/exclusion criteria avoided (to facilitate rapid accrual, broader generalization, pragmatic study conduct)? |
|  |  | Is the control group adequate given current evidence and clinical practice (e.g., “standard of care” rather than “no treatment”)? |

| **Study stage II: Planning and feasibility**  **Milestone: Protocol developed and approved by regulatory bodies** | | |
| --- | --- | --- |
|  | | |
| **Dimension** | **Specific question** | **Examples** |
| **Protection of patient safety and rights** | Do the potential short and long term benefits of the study outweigh study burden (due to study visits, intervention, procedures etc.)? |  |
|  | Are patients’ safety and rights protected through the study’s adherence to applicable national and international regulations and laws? | Are study documents (e.g. protocol, participant information etc.) written in accordance with applicable national (and international, if applicable) regulations/laws? |
|  |  | Are informed consent documents written in lay language and easily understandable for study participants? |
|  |  | Has approval been obtained from ethics committee? |
|  |  | Has approval been obtained from regulatory agency (if applicable)? |
|  | Has feasibility been checked thoughtfully based on existing evidence (systematic review)? | Is valid and robust preclinical data present (if applicable)? |
|  |  | Have crucial feasibility aspects (e.g. recruitment) been piloted? |
|  |  | Are recruitment assumptions realistic in a specified timeframe (e.g. empirical data from electronic health records or from pilot study present)? |
|  |  | Have national/ international study registries been checked for studies that could interfere with the planned study? |
|  |  | Do anticipated study costs (preparation, conduct, analysis, dissemination) match with available budget? |
|  |  | Is study cost data related to planning, conduct, analysis, and dissemination planned to be collected (if applicable)? |
|  | Is collection, documentation, and reporting of adverse events/serious adverse events/suspected unexpected serious adverse reactions according to the applicable regulations planned and specified in the protocol? |  |
|  | Are mechanisms established (e.g., through data monitoring committees) that allow early study termination when required and prevent early study termination for inadequate reasons? | Is one or few interim analyses for safety considered? |
|  |  | Is early stopping for benefit with insufficient collection of safety data avoided ^2^? |
| **Relevance /**  **Patient centeredness and involvement** | Has knowledge transfer/use been considered (e.g., plans to take account of results in clinical guidelines)? | Are relevant guideline groups identified and contact established? |
|  |  | Are patient representatives involved in protocol development? |
| **Minimization of bias (internal validity)** | Is statistical analysis pre-specified (using outcomes as defined in concept stage)? | Are outcomes, datasets, subgroups, handling of missing data, etc., pre-specified? |
|  | Is study monitoring (adapted to risk of study, if applicable) planned and documented in a monitoring plan, including by a data monitoring committee? |  |
|  | Is data management planned and documented in a data management plan? |  |
|  | Has minimization of bias been planned for in the study design, taking account of the research question? | Exemplary items according to study type (non-exhaustive): |
|  |  |  |
|  |  | *Randomized Controlled Trials:* |
|  |  | *Please also refer to Cochrane Risk of Bias tool for RCTs ^3^ for full list of items.* |
|  |  | Is randomization adequate and concealed? |
|  |  | Are (known) prognostic factors distributed equally (i.e. are groups prognostically balanced at the start of the trial)? |
|  |  | Is blinding of participants and/or care-givers adequate? |
|  |  | Are concomitant interventions documented? |
|  |  | Is blinding of outcome assessors adequate? |
|  |  | Are plans to minimize losses to follow up present? |
|  |  | Are plans to analyze study participants in groups as randomized present? |
|  |  |  |
|  |  | *Observational studies (incl. cohort studies):* |
|  |  | *Please also refer to ROBINS-I tool ^4^ for full list of items.* |
|  |  | Is collection of data carefully planned, i.e. are all relevant confounders considered and measured? |
|  |  | Are all study participants selected or recruited from the same or similar populations (incl. the same time period)? |
|  |  | Do the study participants represent the cases originated in the community? (e.g. due to issues with healthcare access) |
|  |  | Are inclusion and exclusion criteria pre-specified and applied uniformly to all study participants? |
|  |  | Are plans to minimize losses to follow-up present? |
|  |  | Is timeframe sufficient so that one can reasonably expect to see an association between exposure and outcome if it existed? |
|  |  | For exposures that can vary in amount or level, does the study examine different levels of the exposure as related to the outcome (e.g. categories, or exposure measured as continuous variable)? |
|  |  | Is exposure measured more than once over time? |
|  |  |  |
|  |  | *Diagnostic accuracy studies:* |
|  |  | *Please also refer to QUADAS-2 Risk of Bias tool ^5^ for full list of items.* |
|  |  | Is there an independent, blind comparison between index test and an appropriate gold standard of diagnosis? |
|  |  | Is the diagnostic test evaluated in a representative, and ideally full spectrum of study participants (like those in whom it would be used in practice, spectrum ranging from mild to severe, and early to late cases of target disorder)? |
|  |  | Is a reference standard applied regardless of the index test results (ideally both index test and reference standard should be carried out on all study participants)? |
|  |  | If no, is it planned to follow up study participants for an appropriate period of time (dependent on disease in question) to see if they are truly negative? |
| **Precision** | Are expected treatment effects and event rates in intervention and control groups realistic and estimated based on empirical evidence? | Is sample size realistically estimated and clearly described (incl. assumed treatment effects, references for estimates, power, alpha error, and expected losses to follow-up)? |
|  |  | Is consent rate precisely estimated? |
|  |  | Are treatment effects and/or event rates estimated in both intervention and control groups? |
|  |  | If yes, are they based on evidence such as systematic literature reviews, meta-analysis? |
|  |  | Is rationale for non-inferiority / equivalence design provided (if applicable)? |
|  |  | Is rationale for maximum clinically acceptable difference (equivalence margins) provided (if applicable)? |
|  |  | Is rationale for sample size given if not derived statistically? |
|  | Are recruitment procedures and recruitment monitoring planned to ensure sufficient sample size? |  |
| **Transparency / Access to data** | Does the protocol accord with established standards (e.g. SPIRIT ^6^ or other applicable guidelines depending on study design)? | Is protocol peer-reviewed? |
|  |  | Is publication and accessibility of full study protocol planned? |
|  |  | Does protocol state a plan on how to deal with study publication in case target sample size could not be achieved/study had to be discontinued prematurely? |
|  | Has the study been registered with a publicly accessible study database (e.g., ClinicalTrials.gov)? |  |
|  | Is there a dissemination plan for sharing study information, including the protocol, summary results, and participant-level data? |  |
| **Generalizability**  **(external validity)** | Are study procedures/observations in line with routine practice in the given setting? | Is standard of care/current practice clearly defined? |
|  |  | Are interventions /observations close to foreseen everyday practice? |
|  |  | Is participant follow up close to everyday practice? |

| **Study Stage III: Conduct**  **Milestone: Last patient last visit** | | |
| --- | --- | --- |
|  | | |
| **Dimension** | **Specific question** | **Examples** |
| **Protection of patient safety and rights** | Is respect for and consideration of patient rights, well-being and dignity guaranteed throughout conduct of study? | Are study participants respected at all times, i.e.: |
|  |  | - Is withdrawal from study at any time explicitly permitted? - Are study participants informed about purpose of research, its procedures (including study medication, concomitant medication, emergency management, etc.) and potential risks, benefits and alternatives, and procedures of data sharing*, so that they can make a voluntary decision? - In case of routinely collected data (including biological material), are study participants informed about the further use of their data for research purposes? - Is study participants’ privacy and confidentiality ensured during (and after) study, e.g. through appropriate coding? |
|  | Is patient safety guaranteed throughout conduct of study? | Are study participants informed of newly discovered risks? |
|  |  | Are side effects / Adverse Events/ Serious Adverse Events/ Suspected Unexpected Serious Adverse Reactions etc. monitored and reported to the ethics committee within required timeframes? |
|  | Is the study being conducted according to protocol? |  |
|  | Is there monitoring of compliance of participants and study staff with the protocol? |  |
|  | Are patients’ safety and rights protected through the study’s adherence to applicable national and international regulations and laws? |  |
| **Relevance /**  **Patient centeredness and involvement** | Are there any measures in place to assure study participants’ involvement, cooperation, and feedback throughout conduct of study (e.g. incentives or phone calls)? |  |
| **Minimization of bias (internal validity)** | Are data systematically collected as pre-specified in the protocol? | Are losses to follow-up minimized? |
|  |  |  |
|  |  | Are protocol deviations documented, and reported to the respective institutions? |
|  |  | Are changes in study procedures amended in the protocol? |
|  |  | Are missing data documented by individual outcomes? |
|  |  | Apart from the allocated treatment, are study groups treated equally (e.g. no additional treatments or tests)? |
|  |  | If applicable, are study participants and clinicians kept "blind" to which treatment was being received? |
|  | Is monitoring being conducted according to the prespecified monitoring plan? |  |
| **Precision** | Is enrollment of study participants monitored? | Are formal techniques in place to monitor recruitment centrally and at participating sites? |
|  |  | Are measures in place to allow timely reaction in case recruitment deviates from expectations? |
|  | Is variability of study procedures and measurement error minimized, e.g. by using centralized monitoring strategies? |  |
|  |  |  |
| **Transparency / Access to data** | Is study conduct transparent to all involved parties? | Are protocol amendments or any necessary deviations from the original protocol clearly documented and disseminated to appropriate parties within reporting timelines? |
|  |  | Are internal or external audits planned, conducted and reported? |
|  |  | Is an external and independent Data Monitoring Committee present or reason provided, why it is not needed? |
| **Generalizability (external validity)** | Are numbers of participants through different stages of a study documented (patient flow) including reasons for leaving the study prematurely (if voluntarily provided by patients)? | Is proportion of study participants who declined randomization documented? |
|  |  | Are the reasons for participants leaving the study before its scheduled end documented (if voluntarily provided by patient)? |

*Detail about data sharing included during peer-review process to avoid a conflict between Protection of patient safety and rights (stage IV – Does data sharing adhere to appropriate data protection policies?) and Transparency/access to data (stage II – Is there a dissemination plan for sharing study information, including the protocol, summary results, and participant level data? and stage V - Is dissemination of data and study results maximized?).

| **Study Stage IV: Analysis and Interpretation**  **Milestone: Study data analyzed and interpreted** | | |
| --- | --- | --- |
|  | | |
| **Dimension** | **Specific question** | **Examples** |
| **Protection of patient safety and rights** | Does data sharing adhere to appropriate data protection policies? | Is patient-level data anonymized? |
|  |  | Have other risks for re-identifying participants been minimized? |
| **Relevance /**  **Patient centeredness and involvement** | Are data analyzed to maximize the use of results by different stakeholders? | Are confidence intervals calculated on an absolute scale to gauge the benefit of an intervention for decision makers (e.g. clinicians, patients, policy makers)? |
| **Minimization of bias**  **(internal validity)** | Are the data analyzed as pre-specified in the protocol/statistical analysis plan? | Are post-hoc analyses clearly labelled as such or as exploratory analyses? |
|  |  |  |
|  |  | Is data analysis performed using standard, generally accepted software? |
|  |  | Are data assumptions checked (e.g. normal distribution) as appropriate for planned statistical tests/modelling? |
|  | Has there been statistical adjustment using key confounding variables in the analysis (e.g. multivariable analysis), if applicable? |  |
|  | Does the analysis follow an adequate strategy to deal with participants in whom treatment or follow-up was not in accordance with study protocol? | Is the intention-to-treat principle followed (i.e. all study participants with data analyzed in groups as randomized) in case of a superiority hypothesis? |
|  |  | Are both a per-protocol and an analysis following the intention-to-treat principle conducted in case of a non-inferiority hypothesis? |
|  |  |  |
|  | Have results been interpreted with least possible “spin” (e.g. without intentionally implying greater or lesser effects than have actually been shown by the data)? |  |
| **Precision** | Is the uncertainty of results considered in the analysis? | Are confidence intervals or other measures of uncertainty calculated? |
|  |  | Are reasonable sensitivity analyses for missing data conducted? |
|  |  | Does interpretation adequately reflect uncertainty? |
| **Transparency / Access to data** | Is the analysis code clearly documented and the analysis process reproducible? |  |
|  | Are deviations from the statistical analysis plan or protocol adequately documented and reported? |  |
| **Generalizability**  **(external validity)** | Does the interpretation put the results adequately into context of clinical practice/public health? |  |

| **Study Stage V: Reporting and Knowledge Translation**  **Milestone: Study archived and published** | | |
| --- | --- | --- |
|  | | |
| **Dimension** | **Specific question** | **Examples** |
| **Protection of patient safety and rights** | Is study completion/termination communicated to appropriate parties and documented in registries? | Is study completion/termination reported to ethics committee/regulatory bodies? |
|  |  | Is study completion/termination appropriately documented in national/international registry? |
|  | Are study participants informed about the outcome/main findings of the study in plain language (including treatment allocation of participant, if applicable)? |  |
|  | Do study participants get access to products/interventions after study, if applicable? |  |
| **Relevance /**  **Patient centeredness and involvement** | Do authors critically reflect on research findings (results as well as challenges or mistakes during study conduct) and the implications for future research? |  |
|  | Is the study easily available to decision-/policy-/guideline-makers? | Is the study cited in a clinical guideline? |
|  | Are study patients/patient representatives involved in reporting the study? | Are patient representatives involved in reporting of the study, e.g. in writing of lay term summaries? |
| **Minimization of bias**  **(internal validity)** | Are all outcomes and important study characteristics reported, as prespecified in the protocol (outcome reporting bias prevented)? | Are all patient-relevant outcomes reported as pre-specified in the protocol? |
|  |  | Are important modifications to the protocol (e.g. premature discontinuation) reported (if applicable)? |
| **Precision** | Are absolute and relative treatment effects reported accompanied by confidence intervals? |  |
|  | Is the analysis set of participants clearly specified? | Are the actual numbers of recruited, randomized (if applicable), followed-up, and analyzed participants reported for each outcome and for each treatment group (if applicable)? |
| **Transparency / Access to data** | Is dissemination of data and study results maximized? | Is dissemination maximized through open access? |
|  |  | Is anonymized individual participant-level data made available (data sharing)? |
|  |  | Are study results posted in study registries? |
|  |  | Does publication in journals include full protocol and statistical analysis plan? |
|  |  | Is dissemination maximized through use of alternative media other than medical journals? |
|  |  | Are resulting doctoral/master theses made publicly available (if applicable)? |
|  | Are reporting guidelines followed, to facilitate critical appraisal and reproducibility? | Is reference made to reporting guidelines such as CONSORT (Randomized trials)^7^, STROBE (Observational studies ^8^, STARD (Diagnostic studies ^9^, or PRISMA (Systematic reviews)^10^ depending on the respective study design? |
|  |  | Are detailed methods disclosed in publications (to enable reproducibility)? |
|  | Are selective reporting, “spin,” and plagiarism avoided, and conflicts of interest declared? | Is selective reporting of study results avoided? |
|  |  | Is plagiarism and self-plagiarism avoided? |
|  |  | Are study results independently peer reviewed? |
|  |  | Is “spin” (i.e. reporting to convince readers that the beneficial effect of the experimental treatment is greater than shown by the results) minimized in reporting of results? |
|  |  | Were conflicts of interest declared? |
|  | Is knowledge transfer and exchange fostered? | Is knowledge transfer and exchange fostered through e.g.: |
|  |  | -Community and provider education and outreach |
|  |  | -Facilitation of two-way communication (lay language) with diverse populations and community groups |
|  |  | -Knowledge transfer and exchange among clinical research groups |
|  | Are study records and data sets kept and archived at least for the legally required period of time? |  |
| **Generalizability**  **(external validity)** | Is potential impact on clinical practice / public health outlined in publicly accessible research reports (e.g. journal publication)? |  |
|  | Are characteristics of included participants clearly reported? | Are inclusion and exclusion criteria clearly reported? |
|  |  | Are characteristics of included participants clearly reported? |
|  | Are the results of prespecified subgroup analyses, if applicable, reported to help assess the importance of key participant characteristics (e.g., disease severity, age, or gender)? |  |

| **Quality promoter: Sustainability / Education** | |
| --- | --- |
|  | |
| **Specific question** | **Examples** |
| Are doctoral students, junior researchers, clinicians, or patient advocates actively involved in all stages of a clinical study and reliably supervised/mentored by senior researchers, and are their specific contributions acknowledged appropriately? | Are doctoral students, junior researchers, clinicians, or patient advocates actively involved in study design, planning, conduct, analysis, interpretation and dissemination of results (e.g. publications, conference presentations, reports, or lay summaries)? |
|  | Are doctoral students, junior researchers, or clinicians actively supervised by senior researchers at all stages of a clinical study? |
|  | Are doctoral students, junior researchers, or clinicians mentored as to career options in clinical research (early career development)? |
|  | Are training options and courses in health research methodology available for principal investigators, staff, and patient advocates? |
|  | Are doctoral students, junior researchers, or clinicians mentored to improve awareness about value of clinical research to patients and society as a whole? |
|  | Are processes continuously adapted and improved to changes, developments, issues, and conditions during research continuum (quality by design)? |

| **Quality promoter: Infrastructure** | |
| --- | --- |
|  | |
| **Specific question** | **Examples** |
| Is a quality management system including standard operating procedures in place? | Is all staff continuously trained in applicable standard operating procedures? |
|  | Are there measures in place to control whether the existing Quality Management System is followed? (i.e. internal audits) |
| Are well-trained, experienced, and dedicated principal investigators and study staff present? | Has the principal investigator and/or staff been involved in clinical studies before? |
|  | Is all staff continuously trained in GCP and protocol-related activities, and particularly the informed consent process? |
|  | Is training (e.g. GCP) of each participating investigator and staff member clearly documented? |
|  | Are roles and responsibilities of each participating investigator and staff member clearly documented? |
|  | Are all involved stakeholders well and adequately informed about study procedures and changes? |
| Are expert epidemiologists/methodologists, statisticians, professional data managers, and/or a logistical support unit involved early on? | Are epidemiologists/methodological specialists involved in development of protocol? |
|  | Are statisticians involved in development of protocol? |
|  | Are data managers involved in the development of the data management plan and the setup of the data management system? |
|  | Is a logistical support unit involved in study planning and/or conduct, e.g. through regulatory affairs experts, study nurses, or project managers? |
| Are adequate human, material, and equipment resources available for study conduct? | Is dispense, transport, and storage of investigational medicinal product, if applicable, planned? |
|  | Is availability of study-specific materials, hardware, and facilities planned and secured? |
|  | Is a transparent study budget available and approved by experienced personnel, including costs for experts mentioned above? |
|  | Is funding secured through acquisition of competitive money or through collaboration with e.g. industry partners? |
| Are adequate facilities ensuring data security and privacy in place (including competent and effective IT support to facilitate solutions tailored to specific challenges of individual studies or agreement templates for doctoral students with respect to data privacy and confidentiality)? | Is an electronic database incl. audit trail in place? |
|  | Are participant data coded? |
|  | Is IT support present at site? |
| Is inter-/multidisciplinary collaboration and involvement in study planning and conduct fostered? | Have all relevant stakeholders been involved in protocol development and conduct? (e.g. investigators at other study sites, etc.) |
|  | Is communication between involved staff, sponsor, contractors, and site fostered? |
| Are all institutions involved in the study covered by compulsory liability insurance? |  |
| Is an overview of the existing research infrastructure available and accessible to any researchers with a study idea? |  |

**References**

1. Gargon E. The COMET (Core Outcome Measures in Effectiveness Trials) Initiative. *Maturitas* 2016; **91**(Supplement C): 91-2.

2. Bassler D, Briel M, Montori VM, et al. Stopping randomized trials early for benefit and estimation of treatment effects: systematic review and meta-regression analysis. *Jama* 2010; **303**(12): 1180-7.

3. Higgins JP, Altman DG, Gotzsche PC, et al. The Cochrane Collaboration's tool for assessing risk of bias in randomised trials. *Bmj* 2011; **343**: d5928.

4. Sterne JA, Hernan MA, Reeves BC, et al. ROBINS-I: a tool for assessing risk of bias in non-randomised studies of interventions. *Bmj* 2016; **355**: i4919.

5. Whiting PF, Rutjes AW, Westwood ME, et al. QUADAS-2: a revised tool for the quality assessment of diagnostic accuracy studies. *Ann Intern Med* 2011; **155**(8): 529-36.

6. Chan AW, Tetzlaff JM, Gotzsche PC, et al. SPIRIT 2013 explanation and elaboration: guidance for protocols of clinical trials. *Bmj* 2013; **346**: e7586.

7. Altman DG, Schulz KF, Moher D, et al. The revised CONSORT statement for reporting randomized trials: explanation and elaboration. *Ann Intern Med* 2001; **134**(8): 663-94.

8. Vandenbroucke JP, von Elm E, Altman DG, et al. Strengthening the Reporting of Observational Studies in Epidemiology (STROBE): explanation and elaboration. *Int J Surg* 2014; **12**(12): 1500-24.

9. Cohen JF, Korevaar DA, Altman DG, et al. STARD 2015 guidelines for reporting diagnostic accuracy studies: explanation and elaboration. *BMJ Open* 2016; **6**(11): e012799.

10. Liberati A, Altman DG, Tetzlaff J, et al. The PRISMA statement for reporting systematic reviews and meta-analyses of studies that evaluate health care interventions: explanation and elaboration. *Ann Intern Med* 2009; **151**(4): W65-94.
