# Supplementary material for: Academic response to improving value and reducing waste: A comprehensive framework for INcreasing QUality In patient-oriented academic clinical REsearch (INQUIRE)
Source: PLoS Med. 2018 Jun 7;15(6):e1002580. doi: 10.1371/journal.pmed.1002580 (PMC5991651; doi:10.1371/journal.pmed.1002580)
Supplement: S5 Appendix — (DOCX) [file pmed.1002580.s005.docx]

**S5 Appendix**

**Potential applications of the INQUIRE framework at different levels and stakeholder groups**

| **Box A. Increasing value of research at local level – examples from a Swiss academic hospital** |
| --- |
| Based on the development of the INQUIRE framework, the advisory board of the Department of Clinical Research at the University Hospital Basel is responding to the 2014 Lancet Series’ recommendations by implementing INQUIRE as follows:   - The Department uses INQUIRE to set its research agenda. A review panel applies the specific questions of the INQUIRE framework’s concept stage to assess the excellence potential of RCTs applying for scarce departmental methods stipends. - In the selected studies, the Department requires investigators to submit yearly follow-up reports on study safety, recruitment patterns, and continuation/discontinuation submitted to its advisory board and the local ethics committee. - In accordance to INQUIRE’s criteria, the Department monitors timely study completion and publication/knowledge translation of study results. - Practical challenges faced by study investigators identified during the stipend period or common Departmental consulting services serve as the basis to initiate studies to investigate innovative solutions. These studies aim to generate empirical evidence on single aspects of the framework and whether they eventually improve value or not. - The Department’s methodological support unit is using the specific questions of particular study stages as quality guidance in their consulting activities. For each research stage of a particular study, the support units guide the investigators in applying the framework as a checklist in the conceptualization, planning, conduct, analysis or dissemination of their study. - Finally, individual research groups have implemented INQUIRE and study coordinators are currently applying the framework as a guiding quality checklist for the conduct of both cohort studies and RCTs. |

| **Box B. Possible levels of framework application** | |
| --- | --- |
| **Level of application** | **Description** |
| Stakeholder group | Stakeholder groups differ in interests and needs and may operationalize framework differently, adapted to their setting, whilst still keeping the comprehensiveness of all dimensions represented in the framework (see Box 2 for detailed examples) |
| Indication area | Indication and treatment areas differ in scientific, ethical, organizational, and cultural aspects and therefore require different study designs to answer the scientific question. The framework should be applied to indication areas by making use of the flexibility of weighing the individual domains, and specific questions |
| Study design | Depending on the study design chosen, different weights across dimensions may have to be applied in order to obtain a meaningful assessment of quality, e.g. for studies in which randomization is unethical and which therefore score lower in internal validity. The examples should support users in applying the framework content to their study design. |
| Research stage | The framework is meant to cover all research phases, from planning to dissemination. However, certain future applications (e.g. templates supporting study planning) may focus on one of the three temporal stages described in the framework. |

| Box C.Possible framework applications by stakeholder group | |
| --- | --- |
| Stakeholder | Examples for application |
| Patient organizations / individual patients interested in research participation | - Development of educational material on “good clinical research” - Development of tool/supporting material assisting in the decision of what clinical study to participate in (“how do I know it is a good clinical trial?”), similar to shared-decision aids - Development of “quality labels” for clinical studies issued by patient organizations, i.e. in large international consortia |
| (Inter-)national funding agencies | - Longitudinal assessment of individual studies, research projects, e.g. in the context of excellency programs - Extension of current criteria for assessment of research proposals, funding decisions |
| Academic institutions, medical faculties, clinical trial units | - Development of tools for (longitudinal) assessment of research department, research unit, research groups - Development of tools for (longitudinal) assessment of individual studies, research projects, e.g. for cost-efficient allocation of resources - Provision of report templates - Tools supporting consultancy of trialists at clinical trial units, over entire study lifecycle |
| Regulatory agencies, health technology assessment bodies, payers (i.e. health insurances) | - Refinement of own quality assessment criteria - Development of longitudinal assessment of individual studies, research projects, e.g. for registration purposes |
| Ethics committees | - Refinement of own quality assessment criteria - Development of tools for longitudinal assessment of individual studies and their outcomes |
| Governmental bodies | - Longitudinal assessment of national research program - Guidance on selection process for excellency programs |
| Drug development/Pharmaceutical industry | - Holistic approach to quality assessment, of both internal decision-making as well as operational aspects at sites |
| All stakeholders | - Overarching concept for the development/adaptation of context-specific checklists, or scores for the quality of clinical research - Development of “quality labels” based on theoretical foundation increasing trust in clinical research quality - Creation of publicly accessible “quality registries” - Adaptation and application of framework to study designs other than the traditional study types, e.g. Real-World Evidence |
